# Design, Synthesis, and Biological Evaluation of Novel 1,3,4-Thiadiazole Derivatives as Potential Antitumor Agents against Chronic Myelogenous Leukemia: Striking Effect of Nitrothiazole Moiety

**DOI:** 10.3390/molecules23010059

**Published:** 2017-12-27

**Authors:** Mehlika Dilek Altıntop, Halil Ibrahim Ciftci, Mohamed O. Radwan, Belgin Sever, Zafer Asım Kaplancıklı, Taha F. S. Ali, Ryoko Koga, Mikako Fujita, Masami Otsuka, Ahmet Özdemir

**Affiliations:** 1 Department of Pharmaceutical Chemistry, Faculty of Pharmacy, Anadolu University, Eskişehir 26470, Turkey; belginsever@anadolu.edu.tr (B.S.); zakaplan@anadolu.edu.tr (Z.A.K.); ahmeto@anadolu.edu.tr (A.Ö.); 2 Department of Bioorganic Medicinal Chemistry, School of Pharmacy, Kumamoto University, Kumamoto 862-0973, Japan; hiciftci@kumamoto-u.ac.jp (H.I.C.); mohamedosman251@gmail.com (M.O.R.); tahafarouk1@yahoo.com (T.F.S.A.); kk1205@kumamoto-u.ac.jp (R.K.); 3 Stanford PULSE Institute, SLAC National Accelerator Laboratory, Menlo Park, CA 94025, USA; 4 Department of Chemistry of Natural Compounds, National Research Center, Dokki, 12622 Cairo, Egypt; 5 Department of Medicinal Chemistry, Faculty of Pharmacy, Minia University, 61519 Minia, Egypt; 6 Research Institute for Drug Discovery, School of Pharmacy, Kumamoto University, Kumamoto 862-0973, Japan; mfujita@kumamoto-u.ac.jp

**Keywords:** thiadiazole, thiazole, benzothiazole, Bcr-Abl, kinase inhibitor, leukemia

## Abstract

In an attempt to develop potent antitumor agents, new 1,3,4-thiadiazole derivatives were synthesized and evaluated for their cytotoxic effects on multiple human cancer cell lines, including the K562 chronic myelogenous leukemia cell line that expresses the Bcr-Abl tyrosine kinase. *N*-(5-Nitrothiazol-2-yl)-2-((5-((4-(trifluoromethyl)phenyl)amino)-1,3,4-thiadiazol-2-yl)thio)acetamide (**2**) inhibited the Abl protein kinase with an IC_50_ value of 7.4 µM and showed selective activity against the Bcr-Abl positive K562 cell line. Furthermore, a Bcr-Abl-compound **2** molecular modelling simulation highlighted the anchoring role of the nitrothiazole moiety in bonding and hydrophobic interaction with the key amino acid residues. These results provide promising starting points for further development of novel kinase inhibitors.

## 1. Introduction

Cancer is the fastest growing disease throughout the world. In developed countries, cancer has become the leading cause of death, whereas in developing countries, it is the second leading cause of death after cardiovascular disorders. By 2030, the annual number of new cancer diagnoses is estimated to be 21 million worldwide, with 17 million patients dying of cancer every year and 75 million people living with cancer diagnoses. Due to the increasing global burden of cancer, extensive efforts have been devoted to the discovery of new potent and selective anticancer agents which destroy tumor cells or at least limit their proliferation [1,2,3,4,5].

Protein kinases have emerged as one of the most frequently targeted families of proteins in anticancer drug discovery owing to their role in the regulation of cellular pathways, particularly those involved in signal transduction through the catalysis of phosphorylation reactions. Kinase inhibitors have played an ascendant role in the treatment of cancer and other diseases [6,7,8,9,10,11]. In particular, imatinib is the first approved tyrosine kinase inhibitor that binds to the kinase domain of Bcr-Abl observed in 95% of chronic myelogenous leukemia (CML) patients [12]. After the milestone approval of imatinib, more than 25 anticancer drugs that target kinases have been approved, and several promising candidates are in various stages of clinical evaluation [6,7,8,9,10,11,12].

Thiadiazole, the bioisostere of pyrimidine and oxadiazole, has been studied extensively for more than one hundred years due to its outstanding pharmacological applications. The sulfur atom of the thiadiazole ring imparts improved liposolubility, and the mesoionic nature of 1,3,4-thiadiazoles also allows these compounds to cross cellular membranes and interact with biological targets with distinct affinities. 1,3,4-Thiadiazoles display a broad spectrum of biological activities, including anticancer, antibacterial, antifungal, antiviral, antiepileptic, antidiabetic, analgesic, and anti-inflammatory activities [13,14,15,16,17,18,19]. In particular, recent studies have pointed out the significance of 1,3,4-thiadiazole as a promising scaffold for antitumor drug discovery and development against different cancer cell lines through the inhibition of diverse molecular targets, including histone deacetylase (HDAC), c-Src/Abl tyrosine kinase, focal adhesion kinase (FAK), and tubulin polymerization [13,14,15,16,17,18,19,20,21,22,23,24,25,26,27,28,29]. On the other hand, the diverse therapeutic applications of thiazoles and benzothiazoles have encouraged medicinal chemists to synthesize a large number of thiazole/benzothiazole-based therapeutic agents [30,31,32,33,34,35,36,37,38,39]. In particular, the clinical efficacy of tiazofurin, bleomycins (BLMs), and dasatinib has pointed out the pivotal role of the thiazole scaffold in the field of current cancer research [30,31,32,33]. Recent patents have indicated that thiazole derivatives show potent antitumor activity against different cancer cell lines through the inhibition of kinases, pro-matrix metalloproteinase activation, signal transducer and activator of transcription 3 (STAT3), the Bcl-2 family, and HDACs [32]. In recent years, benzothiazole has also emerged as a privileged scaffold for anticancer drug discovery. Therefore, antitumor effects of benzothiazole derivatives on different cancer cells have been investigated and these studies have led to the discovery of clinical candidates such as Phortress [33,34,35,36,37,38,39].

**Figure 1:**
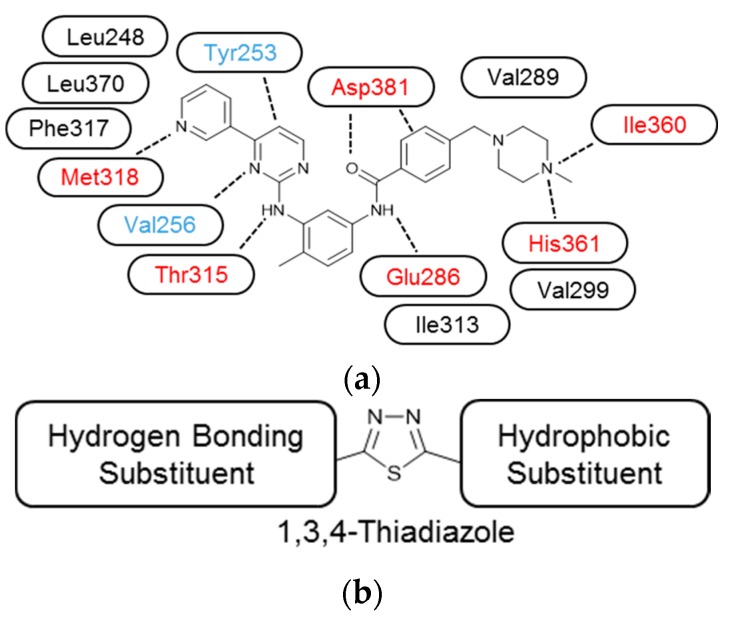
(**a**) Schematic illustration of interaction between imatinib and the kinase domain. Reported interacting amino acids [12] are colored red. Additional interacting amino acids, suggested by MOE calculation, are colored blue. The hydrophobic residues within van der Waals contact reported in [12] are colored black; (**b**) Design of 1,3,4-thiadiazole-based inhibitors.

Prompted by the aforementioned findings, herein we explored the possibility of 1,3,4-thiadiazole as a pivotal scaffold for tyrosine kinase inhibitors. Figure 1a illustrates the imatinib-Bcr-Abl kinase domain interaction; the Met318, Thr315, Asp381, Glu286, His361, and Ile360 hydrogen bonding or CH-π interaction reported [12]; and the Tyr253 and Val256 interactions calculated (see Supplementary Material) together with hydrophobic residues within the van der Waals radii of Leu248, Leu370, Phe317, Val289, Val299, and Ile313 [12]. We reasoned that the introduction of a hydrogen bonding substituent and a hydrophobic substituent into the thiadiazole scaffold would provide promising inhibitors of Bcr-Abl tyrosine kinase (Figure 1b), as will be demonstrated in the next section.

## 2. Results

In the present study, we designed compounds **1**–**10** by means of the introduction of a 4-(trifluoromethyl)phenylamino group as a hydrophobic moiety and *N*-(thiazol-2-yl)-2-mercaptoacetamide as a hydrogen-bonding moiety into 1,3,4-thiadiazole.

**Scheme 1:**
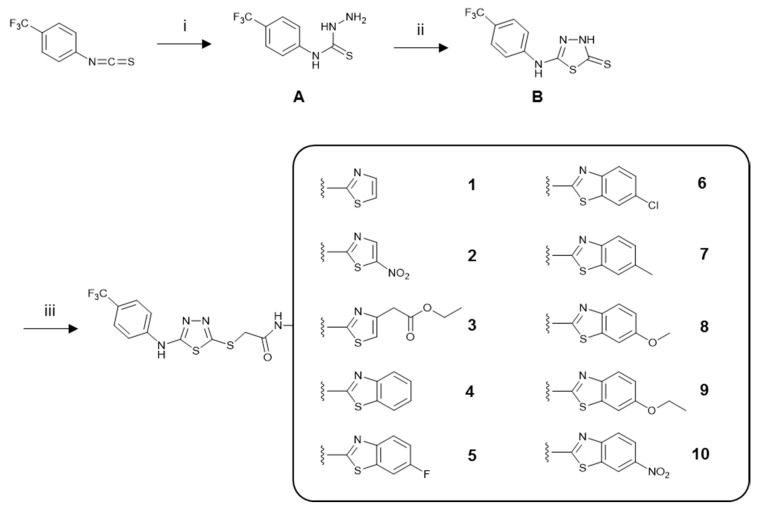
The synthetic route for the preparation of compounds **1**–**10**. Reagents and conditions: (i) NH_2_NH_2_·H_2_O, ethanol, room temperature (r.t.), 4 h; (ii) (1) CS_2_/KOH, ethanol, reflux, 10 h; (2) HCl, pH 4–5; (iii) RNHCOCH_2_Cl, K_2_CO_3_, acetone, r.t., 8 h.

The synthesis of the hitherto unreported compounds (**1**–**10**) was performed as outlined in Scheme 1. Initially, 4-(4-(trifluoromethyl)phenyl)thiosemicarbazide (**A**) was obtained by the reaction of 4-(trifluoromethyl)phenyl isothiocyanate with hydrazine hydrate. Then, 5-(4-(Trifluoromethyl)phenyl)amino-1,3,4-thiadiazole-2(3*H*)-thione (**B**) was synthesized via the ring closure reaction of 4-(4-(trifluoromethyl)phenyl)thiosemicarbazide (**A**) with carbon disulfide in the presence of potassium hydroxide. Finally, the reaction of compound **B** with *N*-(aryl)-2-chloroacetamides in the presence of potassium carbonate afforded new 1,3,4-thiadiazole derivatives (**1**–**10**). The structures of compounds **1**–**10** were confirmed by IR, 1H NMR, 13C NMR, and mass spectral data.

**Figure 2:**
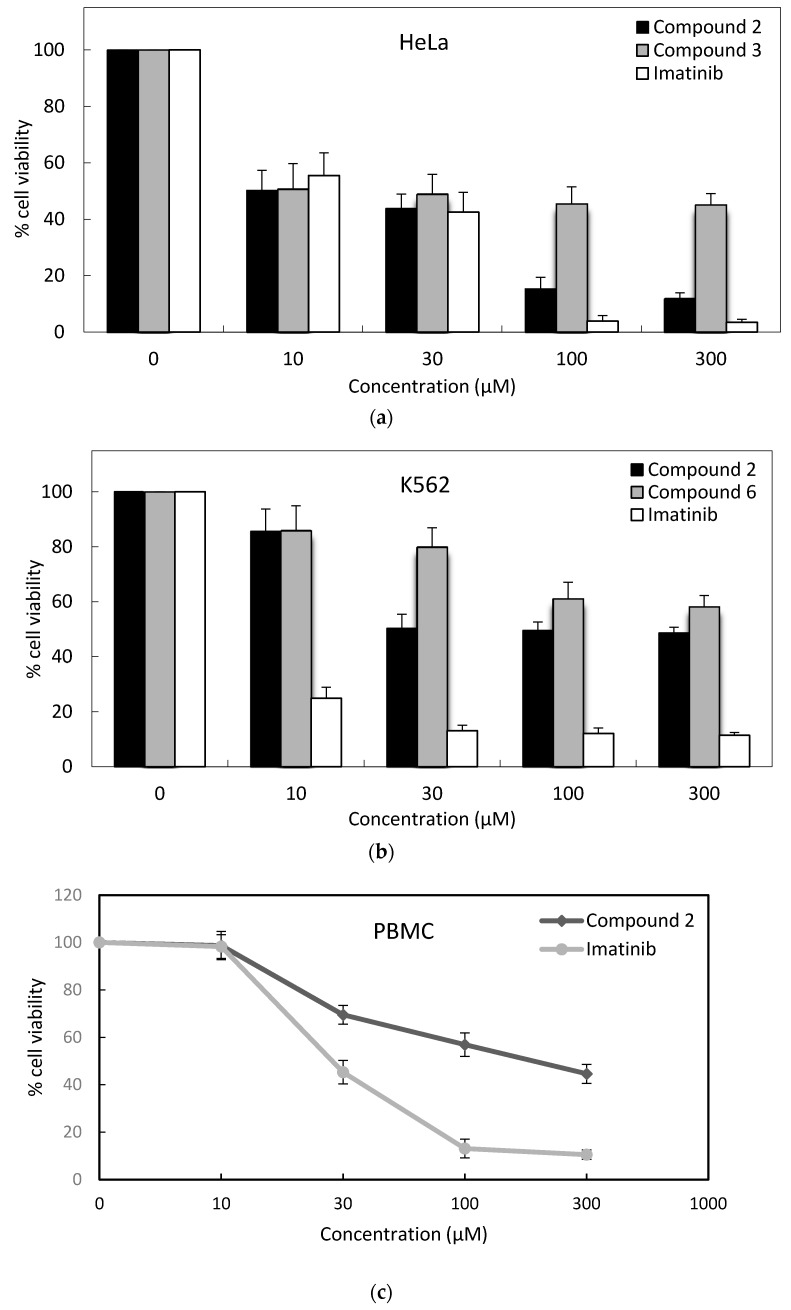
Anticancer effects of tested compounds and imatinib on (**a**) HeLa cells; (**b**) K562 cells; and (**c**) PBMC after 48 h of treatment.

MTT assay was carried out to determine the cytotoxic effects of compounds **1**–**10** on K562 CML and other leukemia cell lines (Jurkat and MT-2) and the HeLa human cervical carcinoma cell line after 48 h of incubation. Imatinib was chosen as a positive control. The results summarized in Table 1 are expressed as concentration-dependent activity in all cases. In general, compounds **5** and **6** exhibited notable antitumor activity against cancer cells except the K562 CML cell line. Compound **6** was found to be the most effective cytotoxic agent on the MT-2 cell line with an IC_50_ value of 38.3 µM. Compounds **2**–**8** were found to possess IC_50_ values lower than 35 µM against the HeLa cell line. In particular, the most active agent was found to be compound **2** with an IC_50_ value of 12.4 µM, followed by compound **3** with an IC_50_ value of 14.1 µM (Figure 2a). Conversely, compounds **1**, **9**, and **10** showed no significant activity against the HeLa cell line. Interestingly, compound **2** showed anticancer activity against the K562 cell line with an IC_50_ value of 33 µM, whereas other compounds were inactive even at 300 µM (Figure 2b). It was striking that compound **2** was the sole active compound in this series against the imatinib-sensitive K562 cell line. Compound **2** exhibited ~5 times lower cytotoxicity on peripheral blood mononuclear cells (PBMC) (IC_50_ = 141.3 µM) than imatinib (IC_50_ = 28.3 µM) as shown in Figure 2c. These results suggest that compound **2** exhibits a significant degree of selective cytotoxicity towards the K562 cell line.

**Figure 3:**
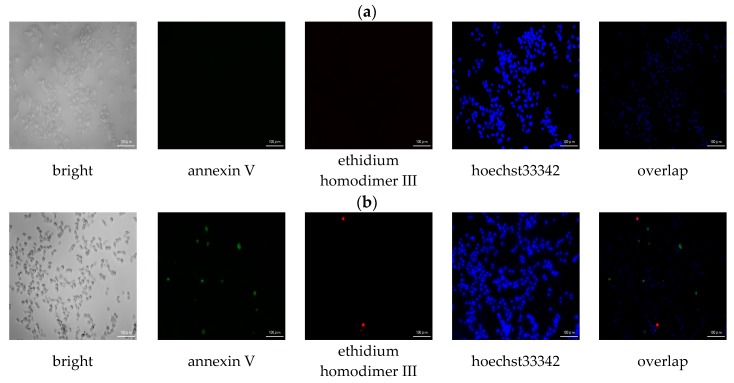

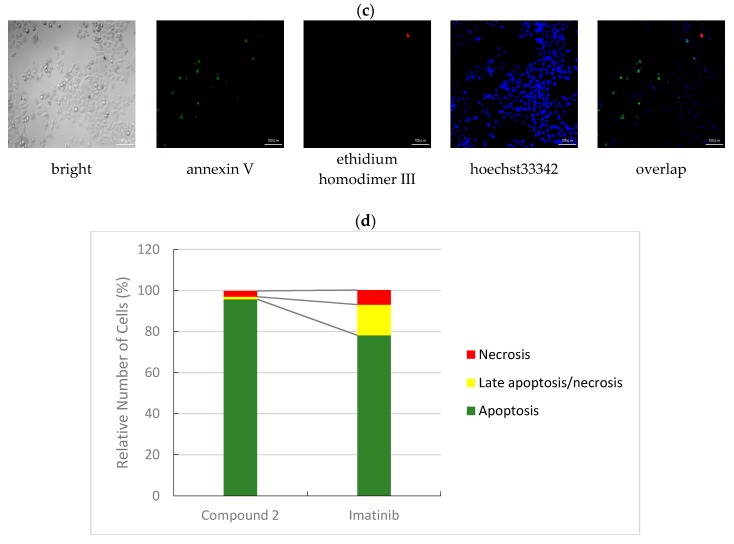
Cellular changes of HeLa cells following exposure to IC_50_ concentrations of control. (**a**) imatinib; (**b**) compound **2**; (**c**) for 3 h; (**d**) a total of approximately 100 stained cells randomly selected in each experiment of (**b**,**c**) were categorized into three types: “apoptosis”, “late apoptosis or necrosis”, and “necrosis”.

Anticancer screening results indicated that compound **2** was the most active anticancer agent, and it was chosen for the evaluation of apoptosis and necrosis in HeLa cells, which was carried out with the annexin V/ethidium homodimer III staining method. Compound **2** and imatinib at the IC_50_ concentrations were incubated with HeLa cells and then were stained and observed by a fluorescence microscope. If the cells are colored red with ethidium homodimer III, and not stained green, the cells are judged to be in necrosis. On the contrary, the completely opposite results indicate apoptosis. The apoptotic and necrotic effects of compound **2** were compared with imatinib at 24 h and 48 h, and the results showed that compound **2** and imatinib both induced late apoptosis or necrosis (data not shown). The apoptotic/necrotic effects of compound **2** and imatinib were also tested on HeLa cells at an earlier time (3 h) at IC_50_ concentrations, and the results indicated that compound **2** showed 96% apoptotic activity at 3 h as illustrated in Figure 3. In contrast, HeLa cells treated with imatinib at 3 h showed 78% apoptotic activity. The results revealed that compound **2** induced apoptosis more strongly than imatinib.

**Figure 4:**
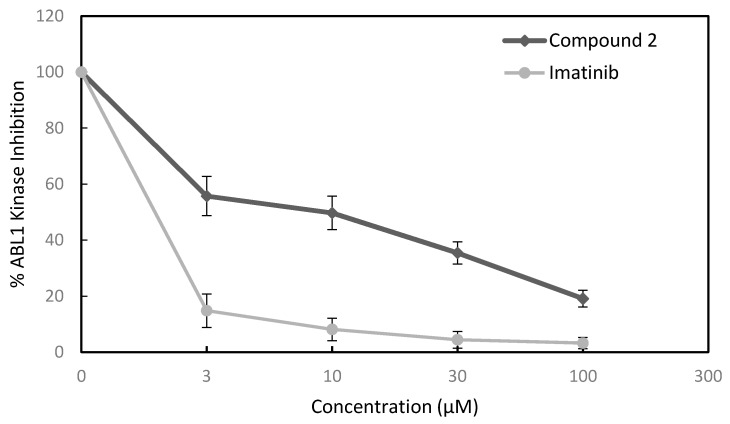
ABL1 kinase inhibition of compound **2** and imatinib at varying concentrations (3 µM, 10 µM, 30 µM, and 100 µM).

**Figure 5:**
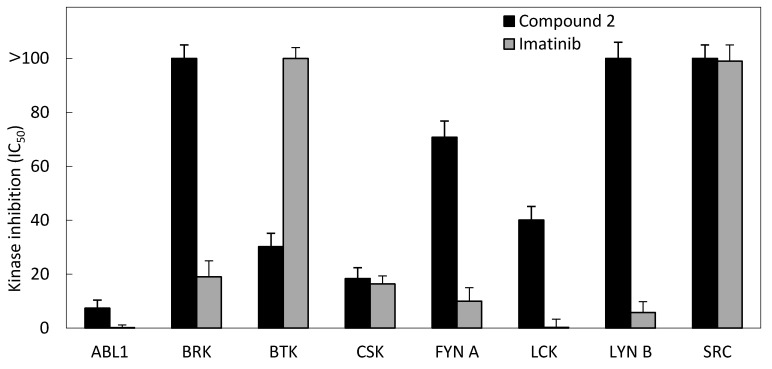
Inhibition of protein kinases by compound **2** and imatinib.

In order to explore the inhibitory potency of compound **2** against kinase enzymes, eight kinases (ABL1, BRK, BTK, CSK, FYN A, LCK, LYN B, and SRC) were selected from a large panel of kinases. Multipoint dose-response experiments using a selected kinase family were tested for inhibition by compound **2**. Compound **2** displayed the most potent inhibitory activity against the ABL1 kinase enzyme with an IC_50_ value of 7.4 µM (Figure 4), and significantly inhibited BTK, CSK, FYN A, and LCK with IC_50_ values in the micromolar range. On the other hand, compound **2** was inactive against BRK, LYN B, and SRC as shown in Table 2. MT-2 has high expression levels of LYN [40], and thus it may be one reason that compound **2** was less effective than imatinib against the MT-2 cell line. These results indicate that compound **2** targets not only ABL, but also inhibits multiple kinases, such as CSK, BTK, and LCK, in the panel of kinases. It can be concluded that compound **2** has a different kinase inhibitor profile than imatinib (Figure 5).

In order to optimize our promising antiproliferative lead compound **2** and investigate the binding modes of the synthesized compounds with the Bcr-Abl tyrosine kinase, a molecular docking simulation study was conducted. The co-crystal structure of imatinib with the Bcr-Abl tyrosine kinase was selected as the docking model (PDB ID code: 1IEP [12]).

**Figure 6:**
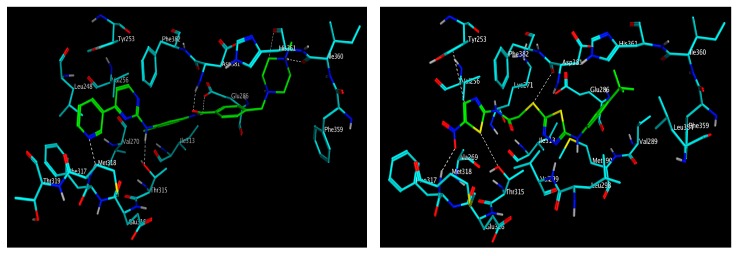
Comparison of binding mode of imatinib (**left**) and compound **2** (**right**) into the ATP binding site of Abl kinase (1IEP). Docking scores are −11.3 and −9.2 kcal/mol, respectively. The key amino acid residues are depicted as cyan sticks. Hydrogen bonds and the CH-π bond are shown as white dashes.

Accordingly, after the validation of the applied computational protocol, compounds **1**, **2**, and **10** were docked into (1IEP) [12,41]. The resultant binding mode of compound **2** reveals the role of the distal nitro group in the formation of a crucial hydrogen bond with Met318. It seems that the presence of the nitro group induces a reorientation of the thiazole moiety to allow a significant hydrogen bond formation with Thr315. One more critical hydrogen bond is established between the sulfur bridge atom and Asp381 residue. The trifluoromethylphenyl group is embedded in the hydrophobic region of the pocket including Ile293, Leu298, and Leu354. Moreover, the CH-π bond with Tyr253 can help stabilize and lock the Abl kinase into its inactive conformation. Based on the docking study, it can be speculated that the high IC_50_ value of compound **2** compared to imatinib can be attributed to the missed interactions with Ile360 and His361. Additionally, the polar thiazole ring of compound **2** has lower affinity than the pyridine moiety of imatinib towards the surrounding hydrophobic residues Phe317, Tyr253, Leu248, and Leu370 (Figure 6).

**Figure 7:**
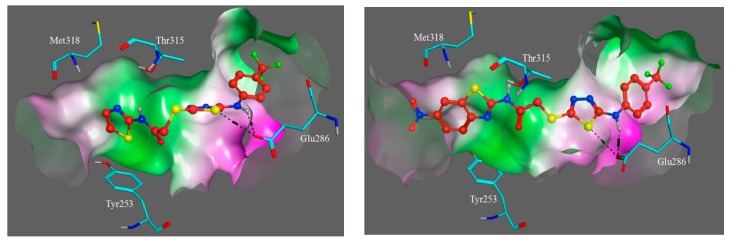
Binding conformations of compound **1** (**left**) and compound **10** (**right**) into the ATP binding site of Abl kinase (1IEP). The key amino acid residues are depicted as cyan sticks. Hydrogen bonds are shown as black dashes. For clarity, the receptor Connolly surface is represented as follows (pink: hydrophilic; green: hydrophobic; white: neutral).

Unlike compound **2**, the docked conformations of compounds **1** and **10** did not form the pivotal interactions with the key amino acid residues, such as Met318 and Tyr253. Both compounds formed only two hydrogen bonds with the carboxylate group of Glu286 and van der Waals interactions between the trifluoromethylphenyl group and the hydrophobic region of the pocket (Figure 7). In conclusion, the absence of or a shift in the distal nitro group abolishes the Abl-kinase inhibitory activity.

## 3. Materials and Methods

### 3.1. Chemistry

All reagents were purchased from commercial suppliers and were used without further purification. Melting points (m.p.) were determined on an Electrothermal 9100 melting point apparatus (Weiss-Gallenkamp, Loughborough, UK) and are uncorrected. IR spectra were recorded on an IRPrestige-21 Fourier Transform Infrared spectrophotometer (Shimadzu, Tokyo, Japan). ^1^H NMR and ^13^C NMR spectra were recorded on a Varian Mercury-400 FT-NMR spectrometer (Agilent, Palo Alto, CA, USA). Mass spectra were recorded on a Shimadzu LCMS-IT-TOF system (Shimadzu, Kyoto, Japan). Thin Layer Chromatography (TLC) was performed on TLC Silica gel 60 F254 aluminium sheets (Merck, Darmstadt, Germany) to check the purity of the compounds.

#### 3.1.1. General Procedure for the Synthesis of the Compounds

##### 4-(4-(Trifluoromethyl)phenyl)thiosemicarbazide (**A**)

A mixture of 4-(trifluoromethyl)phenyl isothiocyanate (0.1 mol) and hydrazine hydrate (0.2 mol) in ethanol (30 mL) was stirred at room temperature for 4 h and then filtered. The residue was crystallized from ethanol [42].

##### 5-(4-(Trifluoromethyl)phenyl)amino-1,3,4-thiadiazole-2(3*H*)-thione (**B**)

4-(4-Trifluoromethylphenyl)thiosemicarbazide (**A**) was dissolved in a solution of potassium hydroxide in ethanol. Carbon disulfide was then added while stirring, and the reaction mixture was heated under reflux for 10 h. The solution was cooled and acidified to pH 4–5 with a hydrochloric acid solution and crystallized from ethanol [43].

##### *N*-(Aryl)-2-chloroacetamides

Chloroacetyl chloride (0.1 mol) was added dropwise with stirring to a mixture of arylamine (0.1 mol) and triethylamine in toluene at 0–5 °C. The solvent was evaporated under reduced pressure. The residue was washed with water to remove triethylamine hydrochloride and crystallized from ethanol [44].

##### *N*-(Aryl)-2-[(5-((4-(trifluoromethyl)phenyl)amino)-1,3,4-thiadiazol-2-yl)thio]acetamide Derivatives (**1**–**10**)

A mixture of *N*-(aryl)-2-chloroacetamide (2 mmol) and 5-(4-trifluoromethylphenyl)amino-1,3,4-thiadiazole-2(3*H*)-thione (**B**) (2 mmol) in acetone was stirred at room temperature for 8 h in the presence of potassium carbonate. The reaction mixture was filtered. The residue was washed with water and crystallized from ethanol [43].

*N-(Thiazol-2-yl)-2-((5-((4-(trifluoromethyl)phenyl)amino)-1,3,4-thiadiazol-2-yl)thio)acetamide* (**1**). Yield: 78%. m.p.: 271.4–272.5 °C. IR ν_max_ (cm^−1^): 3257.77, 3194.12 (N-H stretching), 3076.46 (Aromatic C-H stretching), 2920.23, 2821.86, 2740.85 (Aliphatic C-H stretching), 1674.21 (C=O stretching), 1618.28, 1593.20, 1570.06, 1519.91, 1450.47 (N-H bending, C=N and C=C stretching), 1413.82, 1384.89 (C-H bending), 1330.88, 1267.23, 1238.30, 1178.51, 1159.22, 1107.14, 1068.56, 1047.35, 1008.77 (C-N stretching and aromatic C-H in plane bending), 968.27, 873.75, 831.32, 813.96, 773.46, 717.52, 684.73, 661.58, 626.87 (Aromatic C-H out of plane bending and C-S stretching). ^1^H NMR (400 MHz, DMSO-*d*_6_) δ (ppm): 4.25 (s, 2H), 7.25 (d, *J* = 3.6 Hz, 1H), 7.50 (d, *J* = 3.2 Hz, 1H), 7.68 (d, *J* = 8.8 Hz, 2H), 7.76 (d, *J* = 8.8 Hz, 2H), 10.79 (brs, 1H), 12.43 (brs, 1H). ^13^C NMR (100 MHz, DMSO-*d*_6_): 36.79 (CH_2_), 114.29 (CH), 117.65 (2CH), 122.18 (d, *J* = 31.4 Hz, C), 126.30 (C), 126.89 (d, *J* = 3.9 Hz, 2CH), 138.24 (CH), 143.94 (C), 153.40 (C), 157.75 (C), 164.36 (C), 165.93 (C). HRMS (ESI) (*m*/*z*): [M + H]^+^ calcd. for C_14_H_10_F_3_N_5_OS_3_: 418.0072, found: 418.0077.

*N-(5-Nitrothiazol-2-yl)-2-((5-((4-(trifluoromethyl)phenyl)amino)-1,3,4-thiadiazol-2-yl)thio)acetamide* (**2**). Yield: 88%. m.p.: 275.0–276.5 °C. IR ν_max_ (cm^−1^): 3354.21, 3197.98 (N-H stretching), 3142.04 (Aromatic C-H stretching), 2933.73, 2864.29 (Aliphatic C-H stretching), 1681.93 (C=O stretching), 1614.42, 1558.48, 1508.33, 1469.76 (N-H bending, C=N and C=C stretching), 1411.89, 1381.03, 1352.10 (C-H bending), 1321.24, 1298.09, 1269.16, 1238.30, 1180.44, 1159.22, 1114.86, 1093.64, 1066.64 (C-N stretching and aromatic C-H in plane bending), 968.27, 840.96, 812.03, 779.24, 767.67, 738.74, 723.31, 682.80, 663.51 (Aromatic C-H out of plane bending and C-S stretching). ^1^H NMR (400 MHz, DMSO-*d*_6_) δ (ppm): 4.10 (s, 2H), 7.68 (d, *J* = 8.8 Hz, 2H), 7.77 (d, *J* = 8.8 Hz, 2H), 8.45 (s, 1H), 10.74 (s, 1H), 13.11 (brs, 1H). ^13^C NMR (100 MHz, DMSO-*d*_6_): 41.50 (CH_2_), 117.07 (2CH), 121.51 (d, *J* = 31.4 Hz, C), 125.87 (C), 126.39 (d, *J* = 3.9 Hz, 2CH), 136.65 (C), 143.64 (C), 145.80 (CH), 155.41 (C), 163.95 (2C), 174.18 (C). HRMS (ESI) (*m*/*z*): [M + H]^+^ calcd. for C_14_H_9_F_3_N_6_O_3_S_3_: 462.9923, found: 462.9926.

*Ethyl 2-(2-(2-((5-((4-(trifluoromethyl)phenyl)amino)-1,3,4-thiadiazol-2-yl)thio)acetamido)thiazol-4-yl)acetate* (**3**). Yield: 87%. m.p.: 222.3–224.1 °C. IR ν_max_ (cm^−1^): 3246.20, 3196.05 (N-H stretching), 3140.11 (Aromatic C-H stretching), 2922.16, 2848.86, 2719.63 (Aliphatic C-H stretching), 1716.65 (C=O stretching), 1685.79 (C=O stretching), 1618.28, 1560.41, 1521.84, 1498.69 (N-H bending, C=N and C=C stretching), 1413.82, 1367.53 (C-H bending), 1317.38, 1259.52, 1207.44, 1161.15, 1114.86, 1066.64, 1058.92, 1028.06 (C-N, C-O stretching and aromatic C-H in plane bending), 958.62, 937.40, 877.61, 840.96, 792.74, 744.52, 729.09, 682.80, 632.65 (Aromatic C-H out of plane bending and C-S stretching). ^1^H NMR (400 MHz, DMSO-*d*_6_) δ (ppm): 1.18 (t, *J* = 6.8 Hz, 7.2 Hz, 3H), 3.69 (s, 2H), 4.08 (q, *J* = 6.8 Hz, 2H), 4.21 (s, 2H), 7.01 (s, 1H), 7.68 (d, *J* = 9.2 Hz, 2H), 7.76 (d, *J* = 8.8 Hz, 2H), 10.79 (brs, 1H), 12.48 (brs, 1H). ^13^C NMR (100 MHz, DMSO-*d*_6_): 14.07 (CH_3_), 36.61 (CH_2_), 36.85 (CH_2_), 60.31 (CH_2_), 110.72 (CH), 117.19 (2CH), 121.70 (d, *J* = 32.1 Hz, C), 125.92 (C), 126.42 (d, *J* = 3.2 Hz, 2CH), 143.50 (C), 153.39 (C), 157.49 (C), 164.35 (C), 166.02 (C), 169.98 (2C). HRMS (ESI) (*m*/*z*): [M + H]^+^ calcd. for C_18_H_16_F_3_N_5_O_3_S_3_: 504.0440, found: 504.0445.

*N-(Benzothiazol-2-yl)-2-((5-((4-(trifluoromethyl)phenyl)amino)-1,3,4-thiadiazol-2-yl)thio)acetamide* (**4**). Yield: 85%. m.p.: 293.8–294.5 °C. IR ν_max_ (cm^−1^): 3232.70, 3182.55 (N-H stretching), 3138.18, 3066.82, 3037.89 (Aromatic C-H stretching), 2953.02, 2916.37, 2831.50, 2729.27 (Aliphatic C-H stretching), 1681.93 (C=O stretching), 1602.85, 1566.20, 1444.68 (N-H bending, C=N and C=C stretching), 1417.68, 1382.96 (C-H bending), 1323.17, 1267.23, 1232.51, 1163.08, 1109.07, 1068.56, 1047.35, 1012.63 (C-N stretching and aromatic C-H in plane bending), 983.70, 873.75, 846.75, 833.25, 802.39, 756.10, 727.16, 678.94, 657.73 (Aromatic C-H out of plane bending and C-S stretching). ^1^H NMR (400 MHz, DMSO-*d*_6_) δ (ppm): 4.32 (s, 2H), 7.32 (t, *J* = 7.6 Hz, 1H), 7.45 (t, *J* = 7.6 Hz, 1H), 7.67 (d, *J* = 8.8 Hz, 2H), 7.74–7.78 (m, 3H), 7.98 (d, *J* = 7.6 Hz, 1H), 10.79 (brs, 1H), 12.68 (brs, 1H). ^13^C NMR (100 MHz, DMSO-*d*_6_): 37.03 (CH_2_), 117.17 (2CH), 120.63 (CH), 121.71 (d, *J* = 32.1 Hz, C), 121.75 (CH), 123.68 (CH), 125.81 (CH), 126.18 (C), 126.41 (d, *J* = 3.9 Hz, 2CH), 131.44 (C), 143.46 (C), 148.48 (C), 153.30 (C), 157.77 (C), 164.38 (C), 167.10 (C). HRMS (ESI) (*m*/*z*): [M + H]^+^ calcd. for C_18_H_12_F_3_N_5_OS_3_: 468.0229, found: 468.0213.

*N-(6-Fluorobenzothiazol-2-yl)-2-((5-((4-(trifluoromethyl)phenyl)amino)-1,3,4-thiadiazol-2-yl)thio)acetamide* (**5**). Yield: 87%. m.p.: 309.9–310.8 °C. IR ν_max_ (cm^−1^): 3238.48, 3186.40 (N-H stretching), 3082.25 (Aromatic C-H stretching), 2951.09, 2916.37, 2854.65, 2792.93, 2723.49 (Aliphatic C-H stretching), 1678.07 (C=O stretching), 1608.63, 1589.34, 1566.20, 1450.47 (N-H bending, C=N and C=C stretching), 1419.61, 1382.96 (C-H bending), 1327.03, 1249.87, 1168.86, 1112.93, 1068.56, 1051.20, 1012.63 (C-N stretching and aromatic C-H in plane bending), 983.70, 918.12, 873.75, 835.18, 817.82, 804.32, 748.38, 707.88, 673.16, 659.66 (Aromatic C-H out of plane bending and C-S stretching). ^1^H NMR (400 MHz, DMSO-*d*_6_) δ (ppm): 4.31 (s, 2H), 7.30 (td, *J* = 2.8 Hz, 1H), 7.67 (d, *J* = 8.4 Hz, 2H), 7.74–7.79 (m, 3H), 7.90 (dd, *J* = 2.4, 8.8 Hz, 1H), 10.79 (s, 1H), 12.70 (brs, 1H). ^13^C NMR (100 MHz, DMSO-*d*_6_): 36.99 (CH_2_), 108.21 (d, *J* = 26.9 Hz, CH), 114.31 (d, *J* = 24.4 Hz, CH), 117.17 (2CH), 121.72 (d, *J* = 32.1 Hz, C), 121.77 (d, *J* = 9.6 Hz, CH), 125.82 (C), 126.41 (d, *J* = 3.8 Hz, 2CH), 132.70 (d, *J* = 10.9 Hz, C), 143.47 (C), 145.22 (C), 153.27 (C), 157.50 (C), 159.88 (C), 164.39 (C), 167.22 (C). HRMS (ESI) (*m*/*z*): [M + H]^+^ calcd. for C_18_H_11_F_4_N_5_OS_3_: 486.0135, found: 486.0131.

*N-(6-Chlorobenzothiazol-2-yl)-2-((5-((4-(trifluoromethyl)phenyl)amino)-1,3,4-thiadiazol-2-yl)thio)acetamide* (**6**). Yield: 90%. m.p.: 317.3–318.1 °C. IR ν_max_ (cm^−1^): 3232.70, 3180.62 (N-H stretching), 3136.25, 3072.60, 3035.96 (Aromatic C-H stretching), 2951.09, 2914.44, 2825.72, 2713.84 (Aliphatic C-H stretching), 1678.07 (C=O stretching), 1602.85, 1566.20, 1448.54 (N-H bending, C=N and C=C stretching), 1417.68, 1382.96 (C-H bending), 1325.10, 1265.30, 1236.37, 1165.00, 1111.00, 1099.43, 1068.56, 1051.20, 1012.63 (C-N stretching and aromatic C-H in plane bending), 981.77, 879.54, 846.75, 817.82, 806.25, 765.74, 746.45, 692.44, 659.66 (Aromatic C-H out of plane bending and C-S stretching). ^1^H NMR (400 MHz, DMSO-*d*_6_) δ (ppm): 4.32 (s, 2H), 7.47 (d, *J* = 8.8 Hz, 1H), 7.66–7.77 (m, 5H), 8.14 (s, 1H), 10.80 (s, 1H), 12.79 (brs, 1H). ^13^C NMR (100 MHz, DMSO-*d*_6_): 37.01 (CH_2_), 117.17 (2CH), 121.48 (CH), 121.56 (C), 121.85 (CH), 126.41 (d, *J* = 3.8 Hz, 2CH), 126.54 (C), 127.72 (CH), 129.02 (C), 133.16 (C), 143.46 (C), 147.39 (C), 153.26 (C), 158.70 (C), 164.39 (C), 167.36 (C). HRMS (ESI) (*m*/*z*): [M + H]^+^ calcd. for C_18_H_11_ClF_3_N_5_OS_3_: 501.9839, found: 501.9864.

*N-(6-Methylbenzothiazol-2-yl)-2-((5-((4-(trifluoromethyl)phenyl)amino)-1,3,4-thiadiazol-2-yl)thio)acetamide* (**7**). Yield: 81%. m.p.: 294.3–295.1 °C. IR ν_max_ (cm^−1^): 3232.70, 3184.48 (N-H stretching), 3138.18, 3068.75, 3034.03 (Aromatic C-H stretching), 2954.95, 2916.37, 2829.57, 2789.07, 2729.27 (Aliphatic C-H stretching), 1681.93 (C=O stretching), 1606.70, 1587.42, 1566.20, 1450.47 (N-H bending, C=N and C=C stretching), 1417.68, 1384.89 (C-H bending), 1325.10, 1269.16, 1234.44, 1165.00, 1111.00, 1070.49, 1047.35, 1012.63 (C-N stretching and aromatic C-H in plane bending), 983.70, 815.89, 748.38, 675.09, 657.73 (Aromatic C-H out of plane bending and C-S stretching). ^1^H NMR (400 MHz, DMSO-*d*_6_) δ (ppm): 2.41 (s, 3H), 4.30 (s, 2H), 7.26 (d, *J* = 8.4 Hz, 1H), 7.64–7.76 (m, 6H), 10.79 (s, 1H), 12.59 (brs, 1H). ^13^C NMR (100 MHz, DMSO-*d*_6_): 20.98 (CH_3_), 37.04 (CH_2_), 117.18 (2CH), 120.29 (CH), 121.35 (CH), 121.72 (d, *J* = 32.1 Hz, C), 125.83 (C), 126.43 (d, *J* = 3.9 Hz, 2CH), 127.53 (CH), 131.59 (C), 133.18 (C), 143.48 (C), 146.45 (C), 153.35 (C), 156.92 (C), 164.39 (C), 166.96 (C). HRMS (ESI) (*m*/*z*): [M + H]^+^ calcd. for C_19_H_14_F_3_N_5_OS_3_: 482.0385, found: 482.0366.

*N-(6-Methoxybenzothiazol-2-yl)-2-((5-((4-(trifluoromethyl)phenyl)amino)-1,3,4-thiadiazol-2-yl)thio)acetamide* (**8**). Yield: 83%. m.p.: 278.7–280.1 °C. IR ν_max_ (cm^−1^): 3230.77, 3182.55 (N-H stretching), 3072.60 (Aromatic C-H stretching), 2953.02, 2914.44, 2835.36, 2733.13 (Aliphatic C-H stretching), 1681.93 (C=O stretching), 1608.63, 1591.27, 1564.27, 1462.04 (N-H bending, C=N and C=C stretching), 1417.68, 1384.89 (C-H bending), 1327.03, 1265.30, 1170.79, 1163.08, 1109.07, 1068.56, 1033.85, 1012.63 (C-N, C-O stretching and aromatic C-H in plane bending), 983.70, 873.75, 837.11, 819.75, 796.60, 748.38, 707.88, 659.66 (Aromatic C-H out of plane bending and C-S stretching). ^1^H NMR (400 MHz, DMSO-*d*_6_) δ (ppm): 3.81 (s, 3H), 4.30 (s, 2H), 7.04 (d, *J* = 8.4 Hz, 1H), 7.57–7.77 (m, 6H), 10.80 (s, 1H), 12.55 (s, 1H). ^13^C NMR (100 MHz, DMSO-*d*_6_): 37.45 (CH_2_), 56.08 (CH_3_), 105.20 (CH), 115.48 (CH), 117.64 (2CH), 121.73 (CH), 122.18 (d, *J* = 32.0 Hz, C), 126.29 (C), 126.88 (d, *J* = 3.8 Hz, 2CH), 133.24 (C), 143.04 (C), 143.94 (C), 153.81 (C), 156.14 (C), 156.69 (C), 164.85 (C), 167.22 (C). HRMS (ESI) (*m*/*z*): [M + H]^+^ calcd. for C_19_H_14_F_3_N_5_O_2_S_3_: 498.0334, found: 498.0328.

*N-(6-Ethoxybenzothiazol-2-yl)-2-((5-((4-(trifluoromethyl)phenyl)amino)-1,3,4-thiadiazol-2-yl)thio)acetamide* (**9**). Yield: 82%. m.p.: 270.5–271.9 °C. IR ν_max_ (cm^−1^): 3180.62 (N-H stretching), 3070.68 (Aromatic C-H stretching), 2914.44, 2835.36, 2738.92 (Aliphatic C-H stretching), 1683.86 (C=O stretching), 1606.70, 1591.27, 1560.41, 1456.26 (N-H bending, C=N and C=C stretching), 1413.82, 1386.82 (C-H bending), 1323.17, 1259.52, 1211.30, 1170.79, 1109.07, 1066.64, 1041.56, 1012.63 (C-N, C-O stretching and aromatic C-H in plane bending), 983.70, 943.19, 869.90, 840.96, 817.82, 792.74, 750.31, 673.16, 659.66 (aromatic C-H out of plane bending and C-S stretching). ^1^H NMR (400 MHz, DMSO-*d*_6_) δ (ppm): 1.35 (t, *J* = 6.8 Hz, 7.2 Hz, 3H), 4.07 (q, *J* = 7.2 Hz, 2H), 4.29 (s, 2H), 7.03 (dd, *J* = 2.4, 8.8 Hz, 1H), 7.56 (d, *J* = 2.4 Hz, 1H), 7.64–7.68 (m, 3H), 7.76 (d, *J* = 8.8 Hz, 2H), 10.79 (s, 1H), 12.55 (s, 1H). ^13^C NMR (100 MHz, DMSO-*d*_6_): 14.65 (CH_3_), 36.97 (CH_2_), 63.58 (CH_2_), 105.36 (CH), 115.36 (CH), 117.16 (2CH), 121.26 (CH), 121.70 (d, *J* = 32.1 Hz, C), 125.81 (C), 126.41 (d, *J* = 3.9 Hz, 2CH), 132.76 (C), 142.49 (C), 143.45 (C), 153.31 (C), 155.44 (C), 155.59 (C), 164.37 (C), 166.71 (C). HRMS (ESI) (*m*/*z*): [M + H]^+^ calcd. for C_20_H_16_F_3_N_5_O_2_S_3_: 512.0491, found: 512.0494.

*N-(6-Nitrobenzothiazol-2-yl)-2-((5-((4-(trifluoromethyl)phenyl)amino)-1,3,4-thiadiazol-2-yl)thio)acetamide* (**10**). Yield: 92%. m.p.: 311.8–312.5 °C. IR ν_max_ (cm^−1^): 3184.48 (N-H stretching), 3068.75, 3034.03 (Aromatic C-H stretching), 2949.16, 2912.51, 2794.85 (Aliphatic C-H stretching), 1683.86 (C=O stretching), 1608.63, 1583.56, 1564.27, 1519.91, 1448.54 (N-H bending, C=N and C=C stretching), 1415.75, 1384.89 (C-H bending), 1325.10, 1286.52, 1238.30, 1172.72, 1109.07, 1070.49, 1045.42, 1012.63 (C-N, C-O stretching and aromatic C-H in plane bending), 981.77, 904.61, 873.75, 846.75, 825.53, 752.24, 719.45, 688.59, 659.66 (Aromatic C-H out of plane bending and C-S stretching). ^1^H NMR (400 MHz, DMSO-*d*_6_) δ (ppm): 4.36 (s, 2H), 7.67 (d, *J* = 8.8 Hz, 2H), 7.75 (d, *J* = 8.4 Hz, 2H), 7.92 (d, *J* = 9.2 Hz, 1H), 8.29 (d, *J* = 8.8 Hz, 1H), 9.06 (s, 1H), 10.80 (s, 1H), 13.11 (brs, 1H). ^13^C NMR (100 MHz, DMSO-*d*_6_): 37.04 (CH_2_), 117.17 (2CH), 119.09 (CH), 120.71 (CH), 121.71 (d, *J* = 32.0 Hz, C), 121.80 (CH), 125.80 (C), 126.41 (d, *J* = 3.9 Hz, 2CH), 132.20 (C), 143.05 (C), 143.44 (C), 153.17 (C), 153.43 (C), 163.44 (C), 164.40 (C), 167.98 (C). HRMS (ESI) (*m*/*z*): [M + H]^+^ calcd. for C_18_H_11_F_3_N_6_O_3_S_3_: 513.0080, found: 513.0056.

### 3.2. Biochemistry

#### 3.2.1. Cell Cultures

HeLa human cervical carcinoma cells were incubated in Dulbecco’s modified Eagle’s medium (DMEM) (Wako Pure Chemical Industries, Osaka, Japan) supplemented with 10% fetal bovine serum (FBS) (Equitech-Bio, Kerrville, TX, USA). MT-2, Jurkat, and K562 human leukemic cells were incubated in RPMI 1640 (Wako Pure Chemical Industries) supplemented with 10% FBS. Peripheral blood mononuclear cells (PBMC) were incubated in RPMI 1640, and supplemented with 10% fetal bovine serum (FBS) (Biosera, Kansas City, MO, USA) [45]. All media were supplemented with 89 μg/mL streptomycin (Meiji Seika Pharma, Tokyo, Japan), and cells were incubated at 37 °C in a humidified atmosphere of 95% air and 5% CO_2_. Growing cells were plated at 2 × 10^4^ cells/mL into 24-well microtiter tissue culture plates (Iwaki brand Asahi Glass Co., Chiba, Japan) and incubated for 48 h before the addition of the drugs (the optimal cell number for cytotoxicity assays was determined in preliminary experiments). Stock solutions (1 mM, 3 mM, 10 mM, and 30 mM) of the compounds and imatinib (Wako Pure Chemical Industries) were prepared in dimethyl sulfoxide (DMSO; Wako Pure Chemical Industries) and then added to fresh culture medium. The concentration of DMSO in the final culture medium was 1%.

#### 3.2.2. MTT Assay

The level of cellular reduction of 3-(4,5-dimethylthiazol-2-yl)-2,5-diphenyltetrazolium bromide (MTT) (Dojindo Molecular Technologies, Kumamoto, Japan) was quantified as previously described in the literature with small modifications [46]. The tested compounds were incubated with cells in various concentrations for 48 h. The cells were then stained with MTT solution and incubated further for 4 h at 37 °C. The solution was removed, and the formazan crystals were solubilized by an addition of 100 μL DMSO. The absorbance of the converted dye in the living cells was measured at a wavelength of 550 nm using a microplate spectrophotometer Infinitive M1000 (Tecan, Groding, Austria). Cell viability was calculated as a percentage of the viable control cells. All experiments were performed in triplicate, and IC_50_ values were defined as the drug concentrations which reduced absorbance to 50% of control values.

#### 3.2.3. Apoptotic/Necrotic/Healthy Cells Detection Assay

HeLa cells were incubated with compound **2** and imatinib at IC_50_ concentration for 3 h, 24 h, and 48 h. Then, detection of apoptotic/necrotic/healthy cells was performed according to the manufacturer’s instructions (PromoKine, Heidelberg, Germany). After the cells were washed twice with 1× binding buffer, a staining solution containing 50 μL of 1 × binding buffer, 2 μL of FITC-Annexin V solution, 2 μL of ethidium homodimer III solution, and 2 μL of Hoechst 33,342 solution was added and the cells were incubated for 15 min at room temperature (RT), protected from light. The cells were washed with 1 × binding buffer, analyzed by the all-in-one fluorescence microscope Biorevo Fluorescence BZ-9000 (Keyence, Osaka, Japan), and counted as described previously in [47].

#### 3.2.4. Kinase Inhibition Assay

Kinase selectivity profiling system (TK-2) assays were performed according to the manufacturer’s instructions (Promega Corporation, Madison, WI, USA). Multipoint dose-response experiments were performed using eight kinases, namely: ABL1, BRK, BTK, CSK, FYN A, LCK, LYN B, and SRC. Briefly, the kinase and substrate strips were diluted with 95 µL of 2.5× Kinase Buffer and 15 µL of 100 µM ATP solution, respectively. The kinase reaction was performed using 1 µL of compound solution at varying concentrations (15 µM, 30 µM, 150 µM, and 500 µM), 2 µL of kinase working stock, and 2 µL of ATP/substrate working stock. After 1 h of incubation at room temperature, kinase activity was quantified using the ADP-Glo Kinase Assay (Promega Corporation). Kinase inhibition was quantified using an Infinitive M1000 luminescence microplate spectrophotometer (Tecan, Groding, Austria). The concentration of test compounds required to decrease the kinase activity by 50% was determined using ImageJ software and identified as the IC_50_.

### 3.3. Molecular Modelling

The software MOE 2015.10 (Chemical Computing Group, Montreal, Canada) was employed for all molecular docking and visualization procedures. 1IEP was retrieved from the RCSB Brookhaven Protein Data Bank. Before the docking simulations, ligands and the target protein were prepared with the standard protocol of the MOE 2015.10 software, including the addition of hydrogens, the assignment of bond order, and the assessment of the correct protonation state. All docking calculations were performed using default settings implemented in MOE 2015.10 [48,49,50].

## 4. Conclusions

In the current work, new 1,3,4-thiadiazole compounds were synthesized and evaluated for their cytotoxic effects on the K562 CML cell line and other leukemia cell lines (Jurkat and MT-2) and the HeLa human cervical carcinoma cell line. Compound **2** was the most potent anticancer agent and exhibited tumor selectivity. Compound **2** specifically inhibited Abl kinase, and showed preferential antiproliferative activity against K562 cell line expressing the oncogenic kinase Bcr-Abl. Furthermore, this compound exhibited higher BTK kinase inhibitory activity than imatinib. These results suggest that compound **2** has a different kinase inhibitor profile than imatinib.

**Figure 8:**
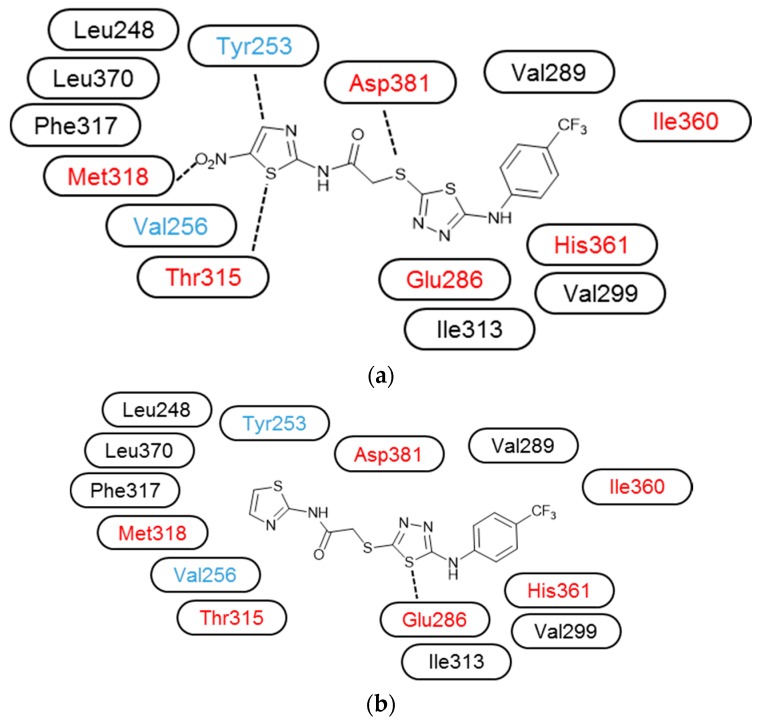

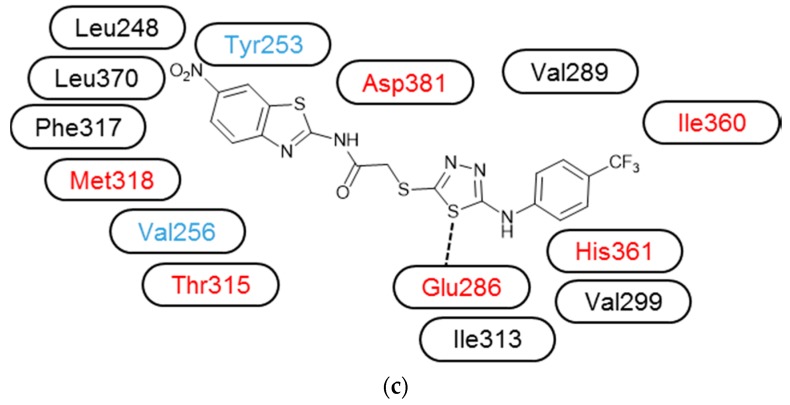
Illustrated binding mode between Bcr-Abl and compound **2** (**a**); compound **1** (**b**); compound **10** (**c**) based on the docking study.

Figure 8 shows the Bcr-Abl binding mode of compounds **2**, **1**, and **10**. As shown in Figure 8a, compound **2** forms hydrogen bonding between the nitrothiazole group and Met318 in addition to Tyr253-thiazole, and Thr315–thiazole interactions and the trifluoromethylphenyl group are surrounded by hydrophobic Val289, Val299, and Ile313 residues. This outcome indicates that our original “hydrogen bonding–thiadiazole–hydrophobic interaction” concept (Figure 1b) is workable. In contrast, compound **1** without the nitro group does not have interaction with Met318, Tyr253, or Thr315 residues and is inactive against the K562 cell line, suggesting the anchoring role of the nitro group. Compound **10** has an intervening benzene ring between the nitro substituent and the thiazole ring, misplacing the nitro group away from the Met318.

According to the obtained biological results and the molecular docking study, the introduction of a nitro substituent into the thiazole scaffold represents an important lead for the discovery of new protein kinase inhibitors, especially because of the emerging resistance to existing drugs. Further research can be carried out on the development of novel effective inhibitors by the optimization of compound **2** to enhance its potency against kinase enzymes and cancer cell lines.

## Figures and Tables

**Table 1:** The cytotoxic effects of compounds **1**–**10** and imatinib after 48 h of incubation.

Compound	IC_50_ (μM) *				
	K562	MT-2	Jurkat	HeLa	PBMC
**1**	>300	>300	>300	135.1 ± 12	-
**2**	33.0 ± 4	166.8 ± 8	17.9 ± 4	12.4 ± 2	141.3 ± 16
**3**	>300	>300	129.4 ± 6	14.1 ± 5	-
**4**	>300	267.3 ± 14	>300	32.9 ± 2	-
**5**	>300	78.5 ± 12	97.9 ± 10	17.3 ± 8	-
**6**	>300	38.3 ± 2	54.4 ± 8	28.7 ± 10	-
**7**	>300	197.9 ± 11	>300	30.2 ± 4	-
**8**	>300	>300	>300	30.9 ± 4	-
**9**	>300	>300	96.1 ± 7	69.4 ± 7	-
**10**	>300	>300	>300	75.3 ± 9	-
Imatinib	5.0 ± 2	9.7 ± 3	6.7 ± 2	15.2 ± 6	28.3 ± 6

* The reported values represent the mean ± SD for each compound based on three independent experiments.

**Table 2:** The protein kinase inhibition of compound **2** and imatinib.

Kinase	IC_50_ (μM)	
	Compound 2	Imatinib
ABL1	7.4	0.2
BRK	>100	19
BTK	30.2	>100
CSK	18.4	16.4
FYN A	70.8	10
LCK	40.1	0.3
LYN B	>100	5.8
SRC	>100	99

## Supplementary Materials

Supplementary materials are available online.

## References

[1] Popat K., McQueen K., Feeley T.W. (2013). The Global Burden of Cancer. Best Pract. Res. Clin. Anaesthesiol..

[2] Teicher B.A. (2006). Cancer Drug Resistance.

[3] Nussbaumer S., Bonnabry P., Veuthey J.L., Fleury-Souverain S. (2011). Analysis of Anticancer Drugs: A Review. Talanta.

[4] Rebucci M., Michiels C. (2013). Molecular Aspects of Cancer Cell Resistance to Chemotherapy. Biochem. Pharmacol..

[5] Holohan C., Van Schaeybroeck S., Longley D.B., Johnston P.G. (2013). Cancer Drug Resistance: An Evolving paradigm. Nat. Rev. Cancer.

[6] Mazola Y., Rodríguez R., Mazola Y. (2008). Protein Kinases as Targets for Drug Design. Biotecnol. Apl..

[7] Krishnamurty R., Maly D.J. (2010). Biochemical Mechanisms of Resistance to Small-Molecule Protein Kinase Inhibitors. ACS Chem. Biol..

[8] Cohen P., Alessi D.R. (2013). Kinase Drug Discovery—What’s Next in the Field?. ACS Chem. Biol..

[9] Gross S., Rahal R., Stransky N., Lengauer C., Hoeflich K.P. (2015). Targeting Cancer with Kinase Inhibitors. J. Clin. Investig..

[10] Wu P., Nielsen T.E., Clausen M.H. (2015). FDA-Approved Small-Molecule Kinase Inhibitors. Trends Pharmacol. Sci..

[11] Wu P., Nielsen T.E., Clausen M.H. (2016). Small-Molecule Kinase Inhibitors: An Analysis of FDA-Approved Drugs. Drug Discov. Today.

[12] Nagar B., Bornmann W.G., Pellicena P., Schindler T., Veach D.R., Miller W.T., Clarkson B., Kuriyan J. (2002). Crystal Structures of the Kinase Domain of c-Abl in Complex with the Small Molecule Inhibitors PD173955 and Imatinib (STI-571). Cancer Res..

[13] Jain A.K., Sharma S., Vaidya A., Ravichandran V., Agrawal R.K. (2013). 1,3,4-Thiadiazole and Its Derivatives: A Review on Recent Progress in Biological Activities. Chem. Biol. Drug Des..

[14] Li Y., Geng J., Liu Y., Yu S., Zhao G. (2013). Thiadiazole-A Promising Structure in Medicinal Chemistry. ChemMedChem.

[15] Hu Y., Li C.Y., Wang X.M., Yang Y.H., Zhu H.L. (2014). 1,3,4-Thiadiazole: Synthesis, Reactions, and Applications in Medicinal, Agricultural, and Materials Chemistry. Chem. Rev..

[16] Matysiak J. (2015). Biological and Pharmacological Activities of 1,3,4-Thiadiazole Based Compounds. Mini-Rev. Med. Chem..

[17] Haider S., Alam M.S., Hamid H. (2015). 1,3,4-Thiadiazoles: A Potent Multi Targeted Pharmacological Scaffold. Eur. J. Med. Chem..

[18] Dawood K.M., Farghaly T.A. (2017). Thiadiazole Inhibitors: A Patent Review. Expert Opin. Ther. Pat..

[19] Aliabadi A. (2016). 1,3,4-Thiadiazole Based Anticancer Agents. Anti-Cancer Agents Med. Chem..

[20] Sun J., Yang Y.S., Li W., Zhang Y.B., Wang X.L., Tang J.F., Zhu H.L. (2011). Synthesis, Biological Evaluation and Molecular Docking Studies of 1,3,4-Thiadiazole Derivatives Containing 1,4-Benzodioxan as Potential Antitumor Agents. Bioorg. Med. Chem. Lett..

[21] Juszczak M., Matysiak J., Szeliga M., Pożarowski P., Niewiadomy A., Albrecht J., Rzeski W. (2012). 2-Amino-1,3,4-thiadiazole Derivative (FABT) Inhibits the Extracellular Signal-Regulated Kinase Pathway and Induces Cell Cycle Arrest in Human Non-Small Lung Carcinoma Cells. Bioorg. Med. Chem. Lett..

[22] Zhang K., Wang P., Xuan L.N., Fu X.Y., Jing F., Li S., Liu Y.M., Chen B.Q. (2014). Synthesis and Antitumor Activities of Novel Hybrid Molecules Containing 1,3,4-Oxadiazole and 1,3,4-Thiadiazole Bearing *Schiff* Base Moiety. Bioorg. Med. Chem. Lett..

[23] Yadagiri B., Gurrala S., Bantu R., Nagarapu L., Polepalli S., Srujana G., Jain N. (2015). Synthesis and Evaluation of Benzosuberone Embedded with 1,3,4-Oxadiazole, 1,3,4-Thiadiazole and 1,2,4-Triazole Moieties as New Potential Anti Proliferative Agents. Bioorg. Med. Chem. Lett..

[24] Kumar D., Kumar N.M., Noel B., Shah K. (2012). A Series of 2-Arylamino-5-(indolyl)-1,3,4-thiadiazoles as Potent Cytotoxic Agents. Eur. J. Med. Chem..

[25] Guan P., Sun F., Hou X., Wang F., Yi F., Xu W., Fang H. (2012). Design, Synthesis and Preliminary Bioactivity Studies of 1,3,4-Thiadiazole Hydroxamic Acid Derivatives as Novel Histone Deacetylase Inhibitors. Bioorg. Med. Chem..

[26] Li Y.J., Qin Y.J., Makawana J.A., Wang Y.T., Zhang Y.Q., Zhang Y.L., Yang M.R., Jiang A.Q., Zhu H.L. (2014). Synthesis, Biological Evaluation and Molecular Modeling of 1,3,4-Thiadiazol-2-amide Derivatives as Novel Antitubulin Agents. Bioorg. Med. Chem..

[27] Guan P., Wang L., Hou X., Wan Y., Xu W., Tang W., Fang H. (2014). Improved Antiproliferative Activity of 1,3,4-Thiadiazole-Containing Histone Deacetylase (HDAC) Inhibitors by Introduction of the Heteroaromatic Surface Recognition Motif. Bioorg. Med. Chem..

[28] Radi M., Crespan E., Botta G., Falchi F., Maga G., Manetti F., Corradi V., Mancini M., Santucci M.A., Schenone S. (2008). Discovery and SAR of 1,3,4-Thiadiazole Derivatives as Potent Abl Tyrosine Kinase Inhibitors and Cytodifferentiating Agents. Bioorg. Med. Chem. Lett..

[29] Hosseinzadeh L., Khorand A., Aliabadi A. (2013). Discovery of 2-Phenyl-*N*-(5-(trifluoromethyl)-1,3,4-thiadiazol-2-yl)acetamide Derivatives as Apoptosis Inducers via the Caspase Pathway with Potential Anticancer Activity. Arch. Pharm. Chem. Life Sci..

[30] Ayati A., Emami S., Asadipour A., Shafiee A., Foroumadi A. (2015). Recent Applications of 1,3-Thiazole Core Structure in the Identification of New Lead Compounds and Drug Discovery. Eur. J. Med. Chem..

[31] Das D., Sikdar P., Bairagi M. (2016). Recent Developments of 2-Aminothiazoles in Medicinal Chemistry. Eur. J. Med. Chem..

[32] Morigi R., Locatelli A., Leoni A., Rambaldi M. (2015). Recent Patents on Thiazole Derivatives Endowed with Antitumor Activity. Recent Pat. Anti-Cancer Drug Discov..

[33] Rouf A., Tanyeli C. (2015). Bioactive Thiazole and Benzothiazole Derivatives. Eur. J. Med. Chem..

[34] Noolvi M.N., Patel H.M., Kaur M. (2012). Benzothiazoles: Search for Anticancer Agents. Eur. J. Med. Chem..

[35] Keri R.S., Patil M.R., Patil S.A., Budagumpi S. (2015). A Comprehensive Review in Current Developments of Benzothiazole-Based Molecules in Medicinal Chemistry. Eur. J. Med. Chem..

[36] Sharma P.C., Sinhmar A., Sharma A., Rajak H., Pathak D.P. (2013). Medicinal Significance of Benzothiazole Scaffold: An Insight View. J. Enzyme Inhib. Med. Chem..

[37] Singh M., Singh S.K. (2014). Benzothiazoles: How Relevant in Cancer Drug Design Strategy?. Anticancer Agents Med. Chem..

[38] Bradshaw T.D., Westwell A.D. (2004). The Development of the Antitumour Benzothiazole Prodrug, Phortress, as a Clinical Candidate. Curr. Med. Chem..

[39] Turan-Zitouni G., Özkay Y., Özdemir A., Kaplancıklı Z.A., Altıntop M.D. (2011). Synthesis of Some Benzothiazole Based Piperazinedithiocarbamate Derivatives and Evaluation of Their Anticancer Activities. Lett. Drug Des. Discov..

[40] Fusaki N., Iwamatsu A., Iwashima M., Fujisawa J. (1997). Interaction between Sam68 and Src Family Tyrosine Kinases, Fyn and Lck, in T Cell Receptor Signaling. J. Biol. Chem..

[41] Bursulaya B.D., Totrov M., Abagyan R., Brooks C.L. (2003). Comparative Study of Several Algorithms for Flexible Ligand Docking. J. Comput. Aided Mol. Des..

[42] Kaplancıklı Z.A., Altıntop M.D., Sever B., Cantürk Z., Özdemir A. (2016). Synthesis and In Vitro Evaluation of New Thiosemicarbazone Derivatives as Potential Antimicrobial Agents. J. Chem..

[43] Altıntop M.D., Can Ö.D., Demir Özkay Ü., Kaplancıklı Z.A. (2016). Synthesis and Evaluation of New 1,3,4-Thiadiazole Derivatives as Antinociceptive Agents. Molecules.

[44] Altıntop M.D., Kaplancıklı Z.A., Turan-Zitouni G., Özdemir A., Demirci F., İşcan G., Revial G. (2011). Synthesis of Some Novel Triazole Derivatives and Investigation of Their Antimicrobial Activities. Synth. Commun..

[45] Karabacak M., Altıntop M.D., Çiftçi H.İ., Koga R., Otsuka M., Fujita M., Özdemir A. (2015). Synthesis and Evaluation of New Pyrazoline Derivatives as Potential Anticancer Agents. Molecules.

[46] Ali T.F.S., Iwamura K., Çiftçi H.İ., Koga R., Matsumoto M., Oba Y., Kurosaki H., Fujita M., Okamoto Y., Umezawa K. (2015). Novel Metal Chelating Molecules with Anticancer Activity. Striking Effect of the Imidazole Substitution of the Histidine–Pyridine–Histidine System. Bioorg. Med. Chem..

[47] Tateishi H., Monde K., Anraku K., Koga R., Hayashi Y., Ciftci H.I., DeMirci H., Higashi T., Motoyama K., Arima H. (2017). A Clue to Unprecedented Strategy to HIV Eradication: “Lock-in and Apoptosis”. Sci. Rep..

[48] Radwan M.O., Sonoda S., Ejima T., Tanaka A., Koga R., Okamoto Y., Fujita M., Otsuka M. (2016). Zinc-Mediated Binding of A Low-Molecular-Weight Stabilizer of the Host Anti-Viral Factor Apolipoprotein B mRNA-Editing Enzyme, Catalytic Polypeptide-Like 3G. Bioorg. Med. Chem..

[49] Bayrak N., Yildirim H., Tuyun A.F., Kara E.M., Celik B.O., Gupta G.K., Ciftci H.I., Fujita M., Otsuka M., Nasiri H.R. (2017). Synthesis, Computational Study, and Evaluation of *In Vitro* Antimicrobial, Antibiofilm, and Anticancer Activities of New Sulfanyl Aminonaphthoquinone Derivatives. Lett. Drug Des. Discov..

[50] Tanaka A., Radwan M.O., Hamasaki A., Ejima A., Obata E., Koga R., Tateishi H., Okamoto Y., Fujita M., Nakao M. (2017). A novel inhibitor of farnesyltransferase with a zinc site recognition moiety and a farnesyl group. Bioorg. Med. Chem. Lett..

