# Supplementary material for: Design, Synthesis, and Biological Evaluation of Novel 1,3,4-Thiadiazole Derivatives as Potential Antitumor Agents against Chronic Myelogenous Leukemia: Striking Effect of Nitrothiazole Moiety
Source: Molecules. 2017 Dec 27;23(1):59. doi: 10.3390/molecules23010059 (PMC6017545; doi:10.3390/molecules23010059)
Supplement: Supplementary file 1 [file molecules-23-00059-s001.pdf]

Design, Synthesis and Biological Evaluation of Novel 1,3,4-Thiadiazole Derivatives as Potential Antitumor Agents against Chronic Myelogenous Leukemia. Striking Effect of Nitrothiazole Moiety

Mehlika Dilek Altıntop [a,*,†], Halil Ibrahim Ciftci [b,c,†], Mohamed O. Radwan [b,d], Belgin Sever [a], Zafer Asım Kaplancıklı [a], Taha F.S. Ali [b], Ryoko Koga [b], Mikako Fujita [e], Masami Otsuka [b,], and Ahmet Özdemir [a]

[a] *Department of Pharmaceutical Chemistry, Faculty of Pharmacy, Anadolu University, Eskişehir 26470, Turkey*

[b] *Department of Bioorganic Medicinal Chemistry, School of Pharmacy, Kumamoto University, Kumamoto, 862-0973, Japan*

[c] *Stanford PULSE Institute, SLAC National Accelerator Laboratory, Menlo Park, CA, USA*

[d] *Department of Chemistry of Natural Compounds, National Research Center, Dokki 12622, Cairo, Egypt*

[e] *Research Institute for Drug Discovery, School of Pharmacy, Kumamoto University, Kumamoto, 862-0973, Japan*

† These authors contributed equally to this work.

* Corresponding authors.

E-mail address: mdaltintop@anadolu.edu.tr (M.D. Altıntop).

Supporting Information

In order to optimize our promising antiproliferative lead compound **2** and explore the binding modes of the synthesized compounds with Bcr-Abl tyrosine kinase, a molecular docking simulation study was conducted. The co-crystal structure of imatinib with Bcr-Abl tyrosine kinase was selected as the docking model (PDB ID code: 1IEP [1]).

The docking study was started by optimization of the used computational protocol to obtain the most accurate results. For this purpose, we redocked the co-crystallized imatinib into 1IEP [1] ATP binding site. RMSD (root mean square deviation) cutoff of 2 Å is often used as an indicator of the accurate bound structure prediction [2]. Therefore, the docked results were compared with the crystal structure of the bound ligand–protein complex. RMSD of the best docked imatinib conformation was 0.3 Å that looks almost superimposed on the native crystallized pose. This result proved the high accuracy and ability of the employed protocol to reproduce the experimental pose (Fig. S1). Moreover, the obtained binding energy score was quite low being -11.64 kcal/mol.

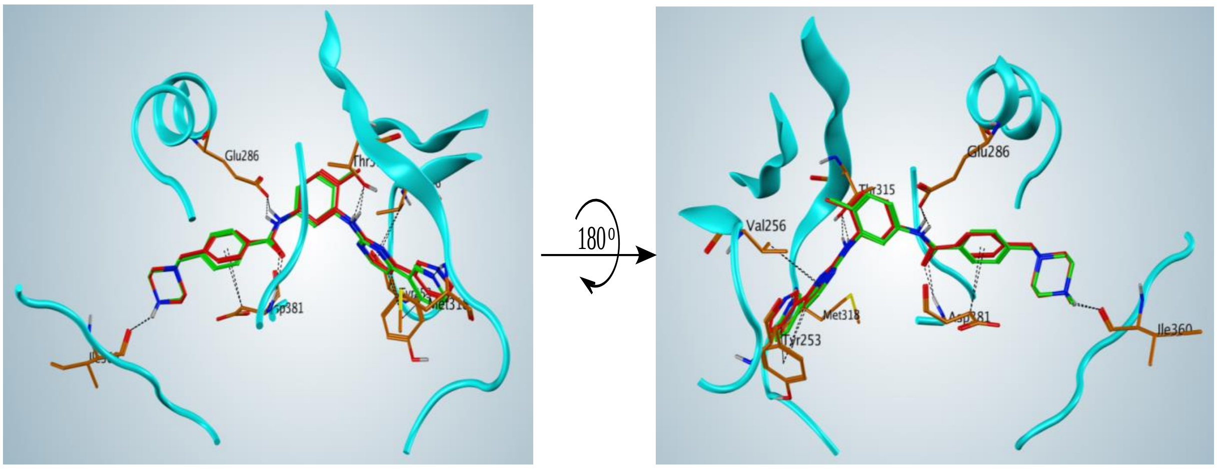

Fig. S1. Superimposed docked (green sticks) and co-crystalized (red sticks) poses of imatinib showing reproducibility of experimental result achieved by the applied MOE docking protocol. The key amino acid residues are shown in brown color and ribbons are in cyan color. The settled hydrogen bonds are shown as black dotted lines. RMSD is 0.3 Å and docking score is -11.64 kcal/mol.

References

1. Nagar, B.; Bornmann, W.G.; Pellicena, P.; Schindler, T.; Veach, D.R.; Miller, W.T.; Clarkson, B.; Kuriyan, J. Crystal structures of the kinase domain of c-Abl in complex with the small molecule inhibitors PD173955 and imatinib (STI-571). *Cancer Res.* **2002**, *62*, 4236–4243.

2. Bursulaya, B.D.; Totrov, M.; Abagyan, R.; Brooks, C.L. Comparative study of several algorithms for flexible ligand docking. *J. Comput. Aided Mol. Des.* **2003**, *17*, 755–763.

Supporting Information

IR Spectrum of Compound **1**

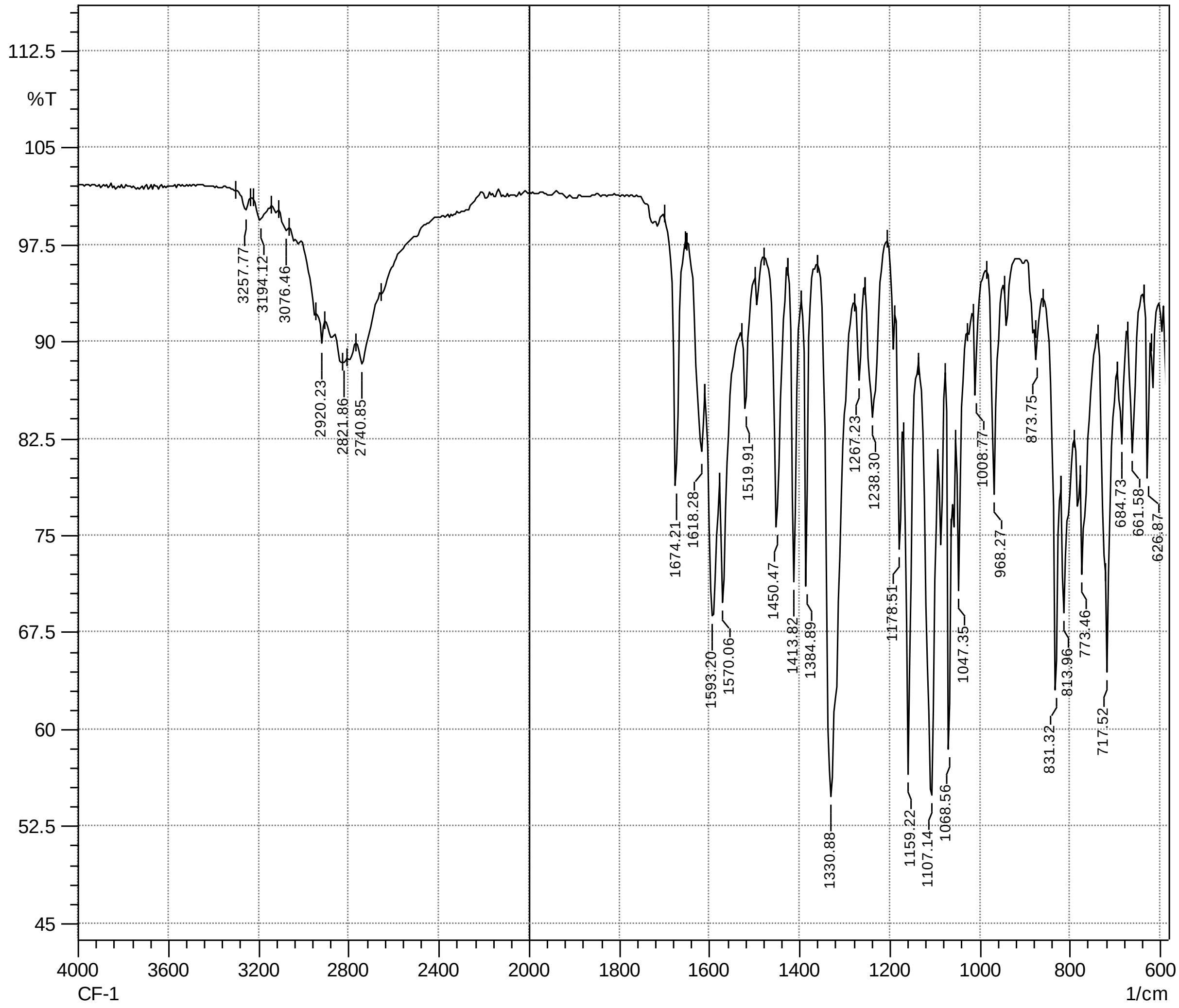

^{1}H NMR Spectrum of Compound **1**

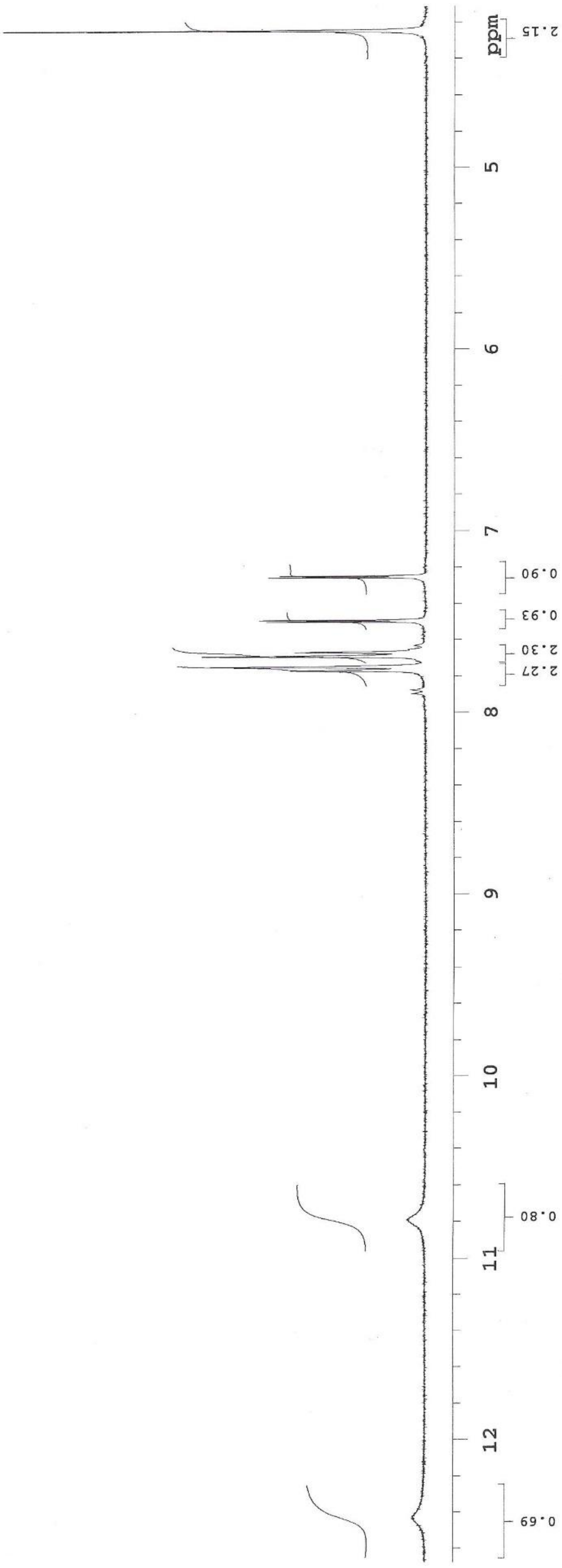

^{13}C NMR Spectrum of Compound **1**

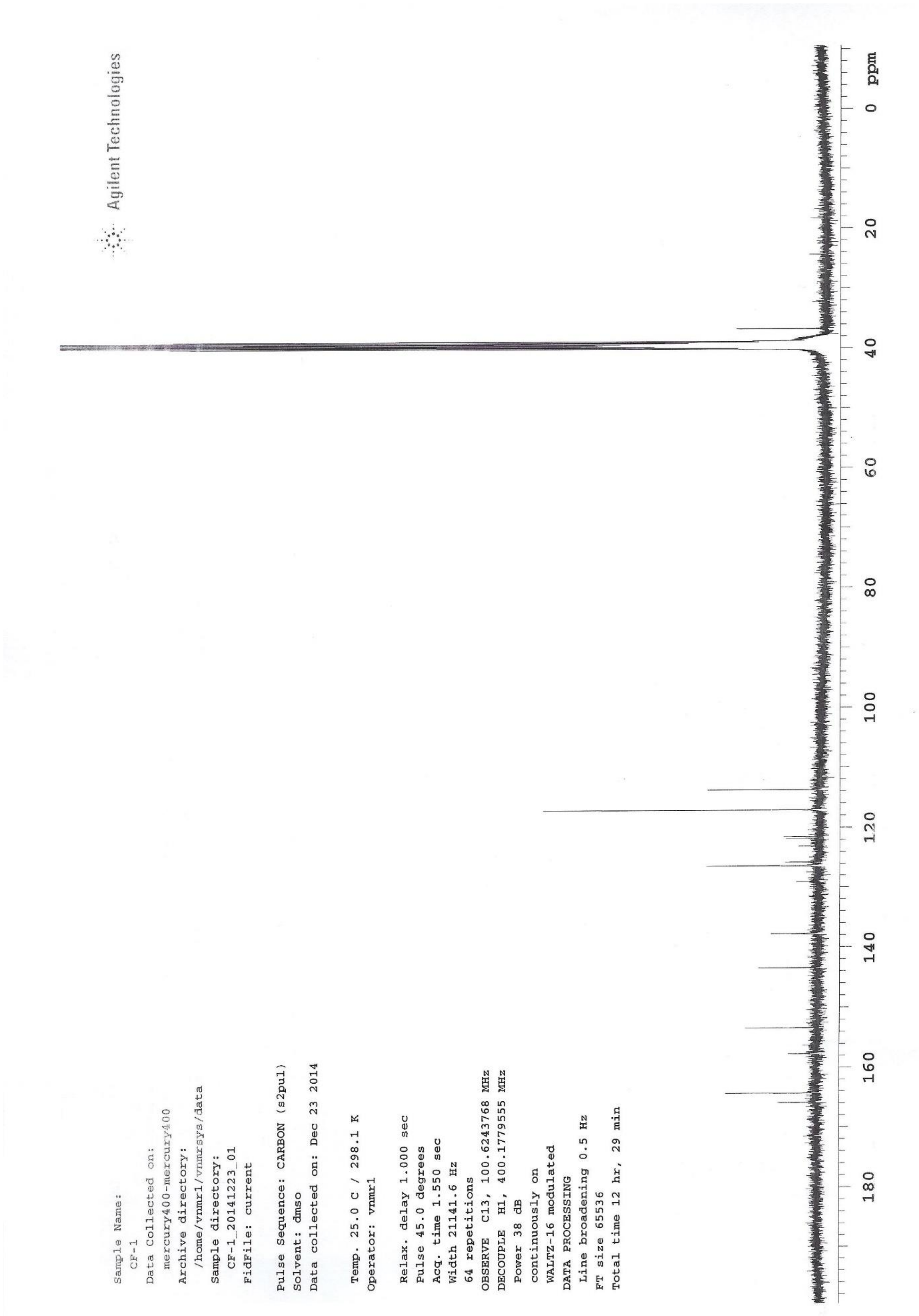

Mass Spectrum of Compound **1**

Formula Predictor Report - CF-1_12.lcd

Data File: C:\LabSolutions\Data\Analiz\mdaltintop\CF-1_12.lcd

Elmt	Val.	Min	Max	Elmt	Val.	Min	Max	Elmt	Val.	Min	Max	Elmt	Val.	Min	Max	Use Adduct
H	1	8	30	O	2	0	3	Cl	1	0	0	I	3	0	0	H
C	4	13	26	F	1	0	4	Br	1	0	0					
N	3	3	5	S	2	1	3	Ru	2	0	0					

Error Margin (ppm): 10
HC Ratio: unlimited
Max Isotopes: 3
MSn Iso RI (%): 10.00

DBE Range: 0.0 - 12.0
Apply N Rule: no
Isotope RI (%): 1.00
MSn Logic Mode: AND

Electron Ions: both
Use MSn Info: no
Isotope Res: 10000
Max Results: 500

Event#: 1 MS(E+) Ret. Time : 6.573 Scan# : 987

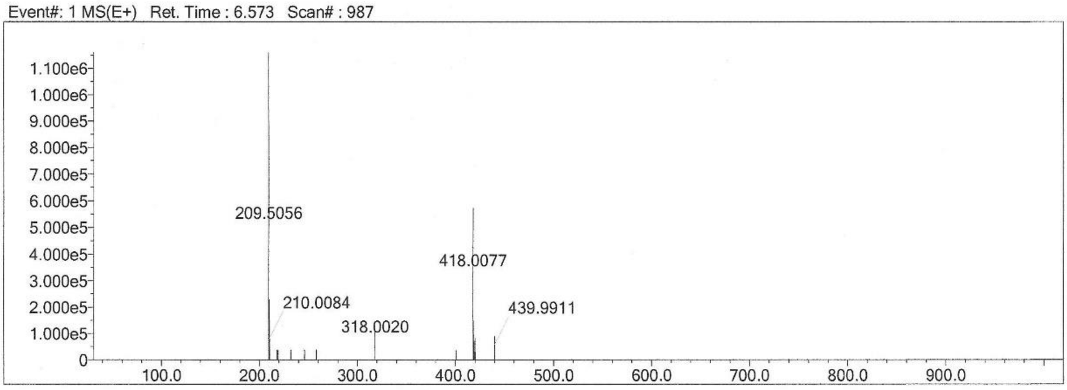

Measured region for 418.0077 m/z

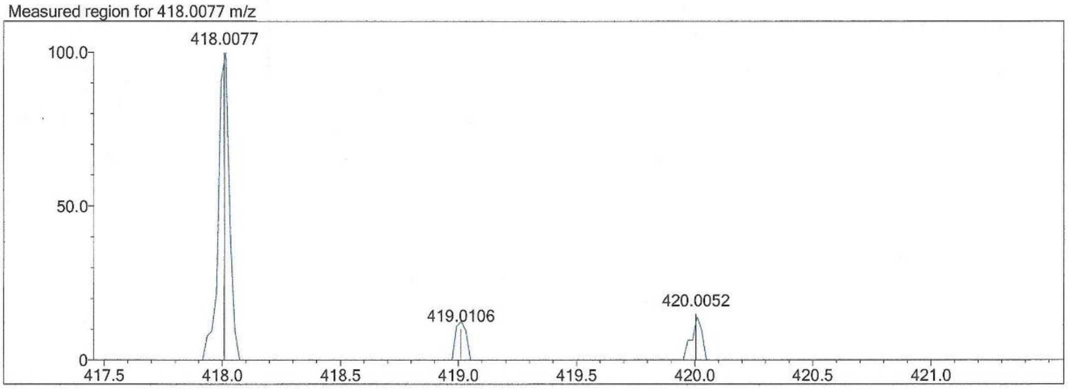

C14 H10 N5 O F3 S3 [M+H]+ : Predicted region for 418.0072 m/z

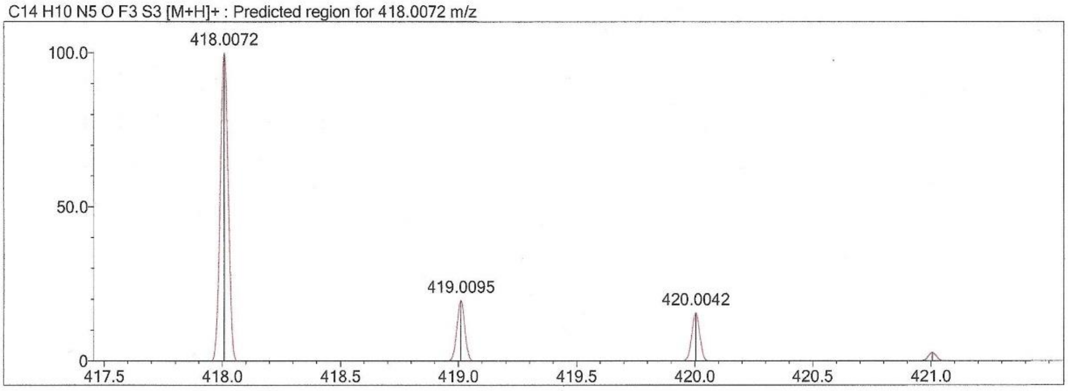

Rank	Score	Formula (M)	Ion	Meas. m/z	Pred. m/z	Df. (mDa)	Df. (ppm)	Iso	DBE
1	44.88	C14 H10 N5 O F3 S3	[M+H]+	418.0077	418.0072	0.5	1.20	45.10	11.0

IR Spectrum of Compound **2**

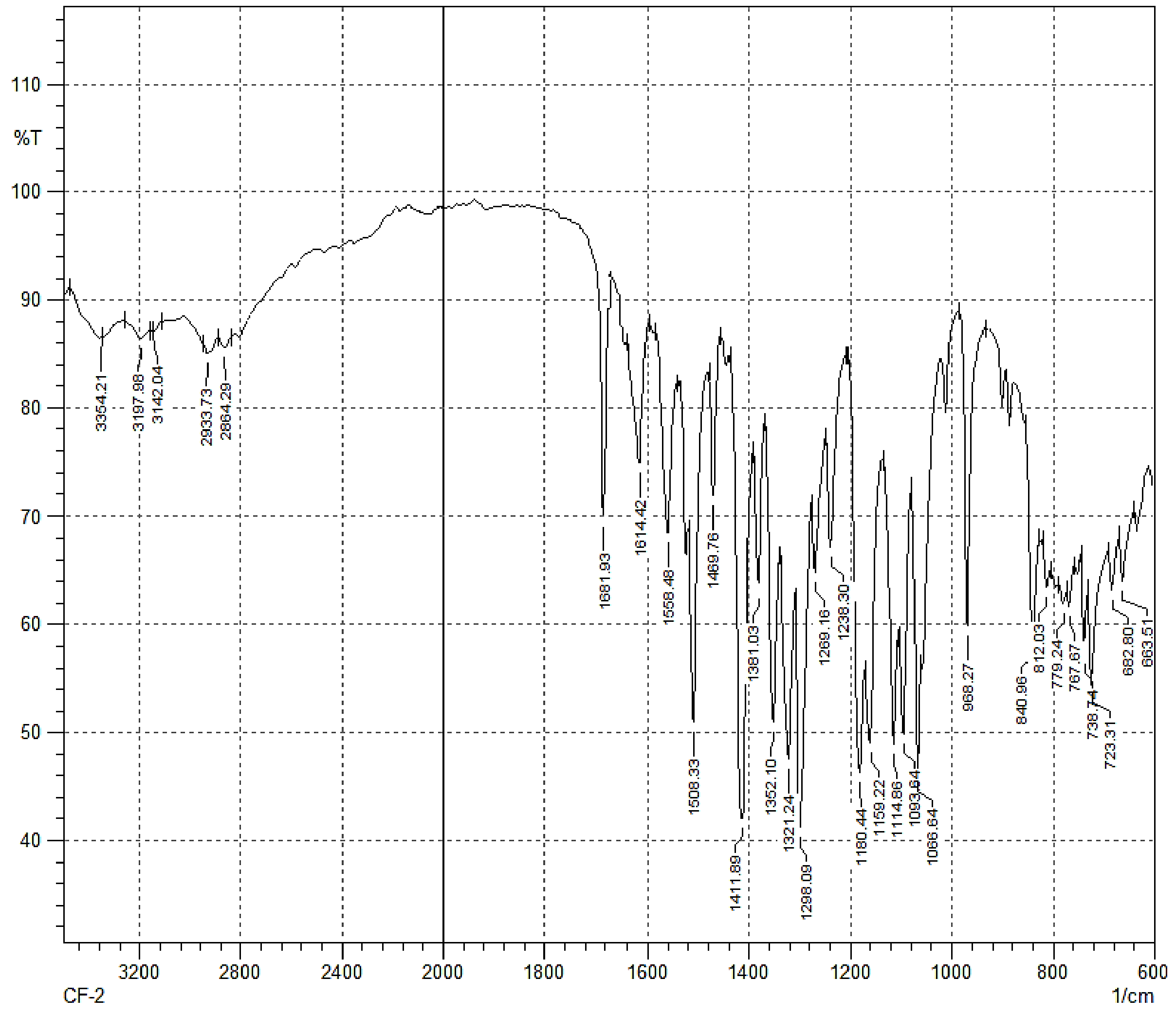

^{1}H NMR Spectrum of Compound **2**

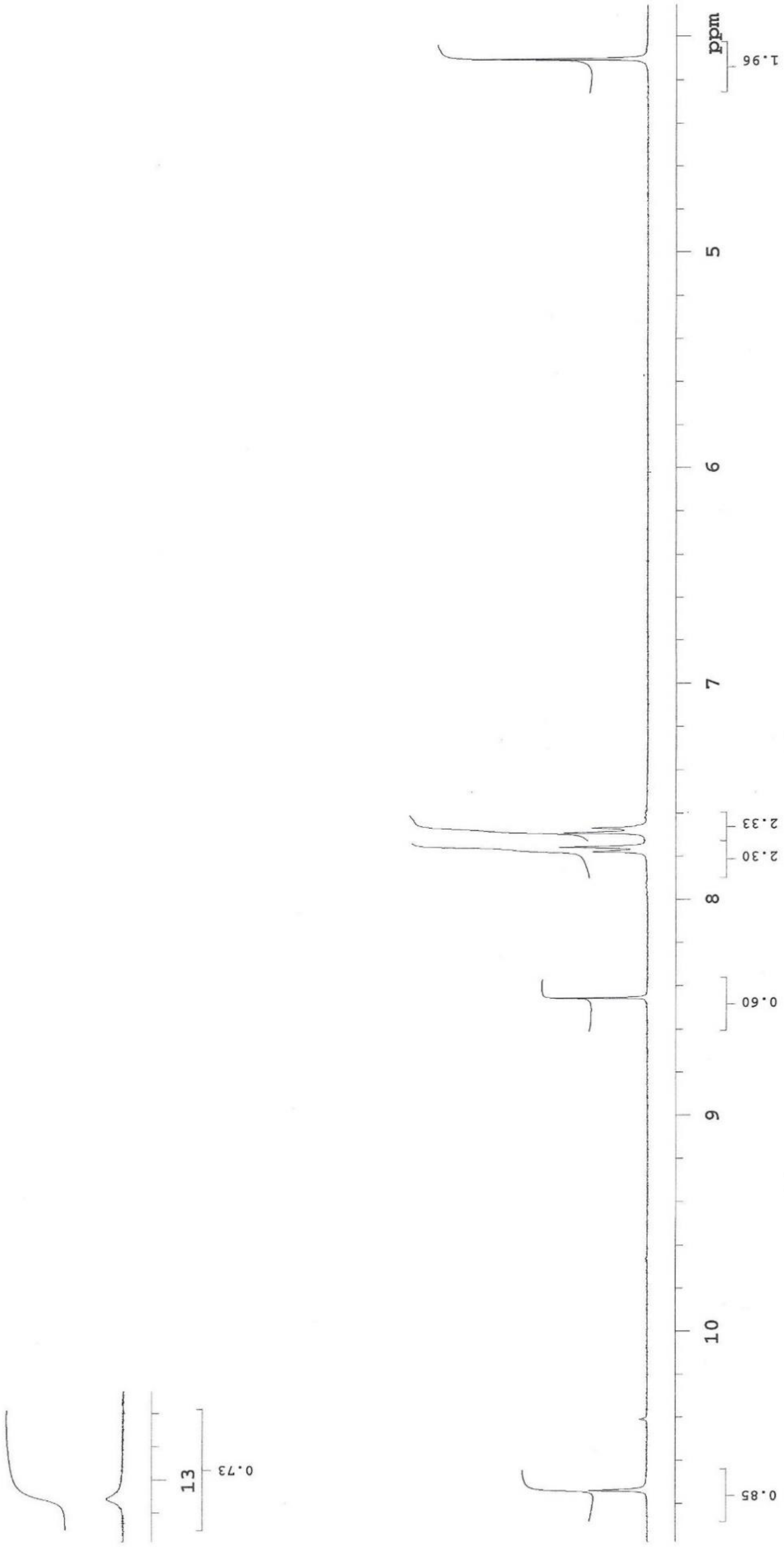

^{13}C NMR Spectrum of Compound **2**

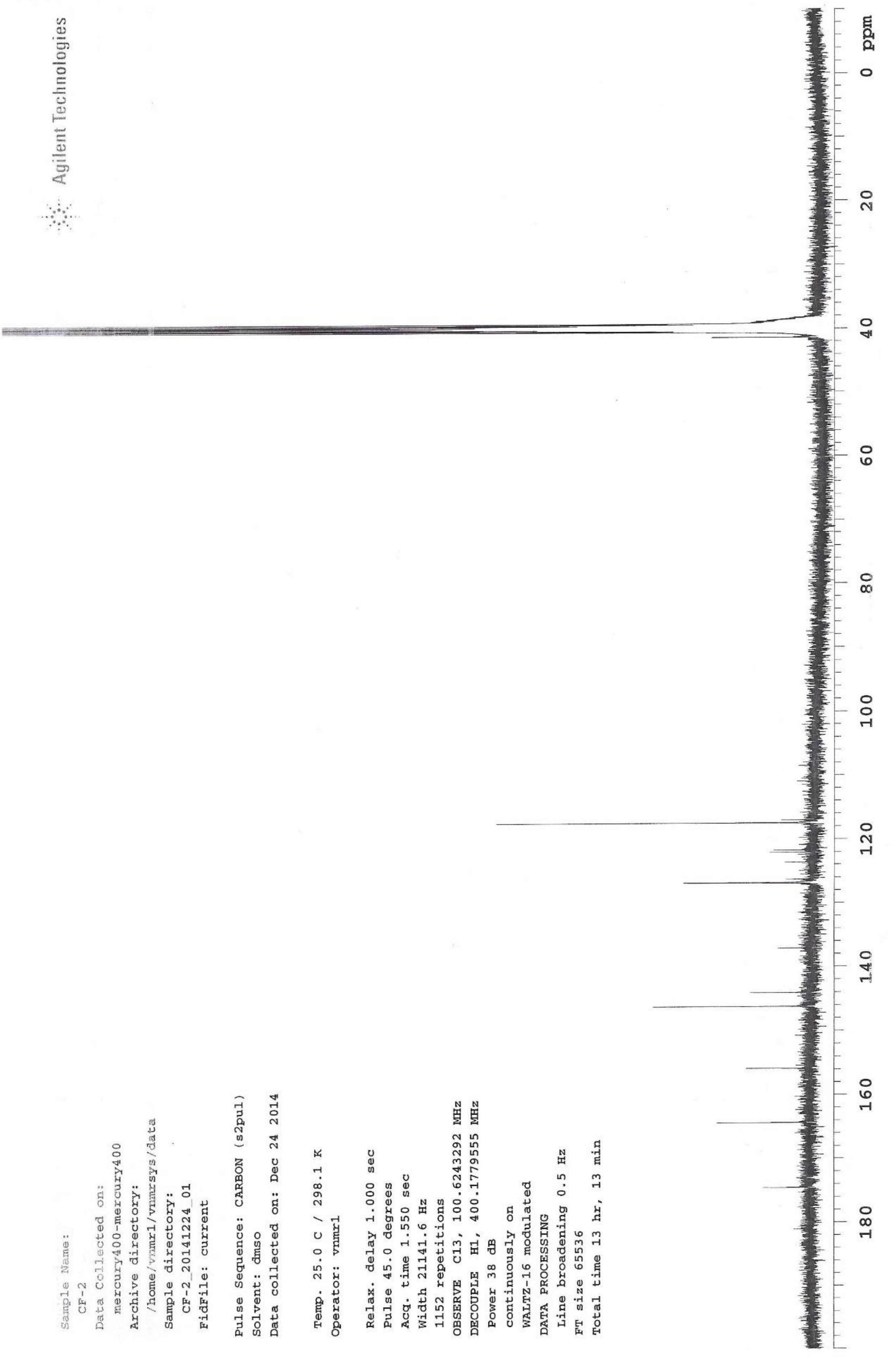

Mass Spectrum of Compound **2**

Formula Predictor Report - CF-2_13.lcd

Data File: C:\LabSolutions\Data\Analiz\mdaltintop\CF-2_13.lcd

Elmt	Val.	Min	Max	Elmt	Val.	Min	Max	Elmt	Val.	Min	Max	Elmt	Val.	Min	Max	Use Adduct
H	1	8	30	O	2	0	3	Cl	1	0	0	I	3	0	0	H
C	4	13	26	F	1	0	4	Br	1	0	0					
N	3	3	6	S	2	1	3	Ru	2	0	0					

Error Margin (ppm):	10	DBE Range:	0.0 - 12.0	Electron Ions:	both
HC Ratio:	unlimited	Apply N Rule:	no	Use MSn Info:	no
Max Isotopes:	3	Isotope RI (%):	1.00	Isotope Res:	10000
MSn Iso RI (%):	10.00	MSn Logic Mode:	AND	Max Results:	500

Event#: 1 MS(E+) Ret. Time : 6.947 Scan# : 1043

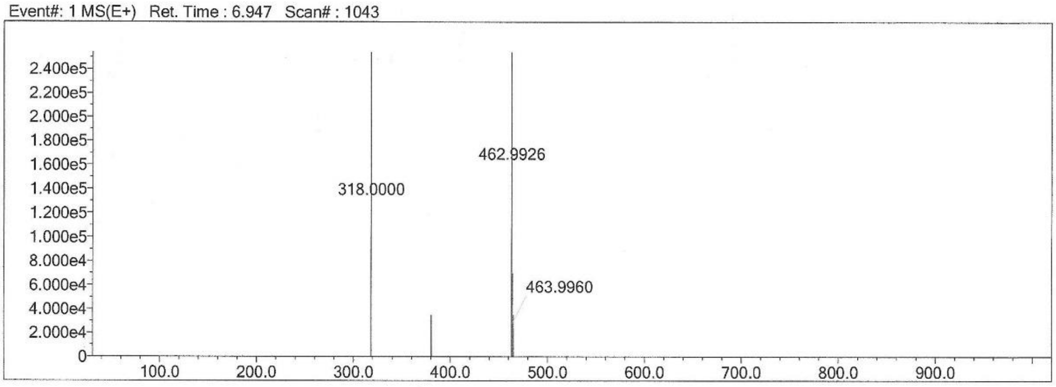

Measured region for 462.9926 m/z

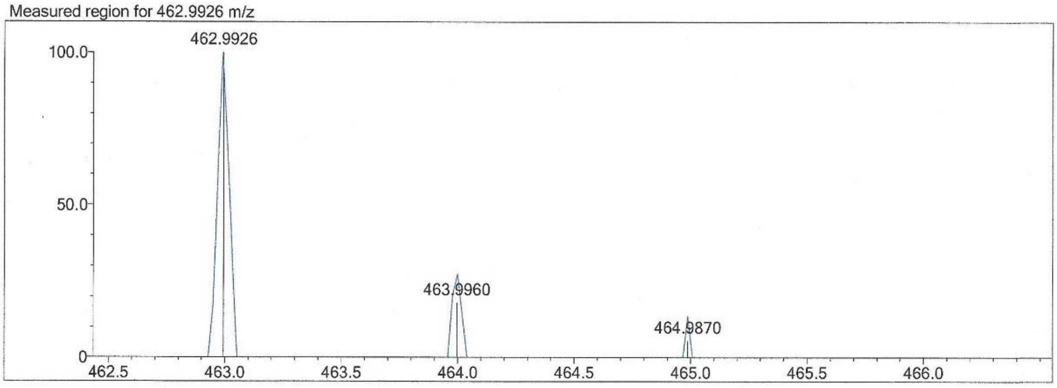

C14 H9 N6 O3 F3 S3 [M+H]+ : Predicted region for 462.9923 m/z

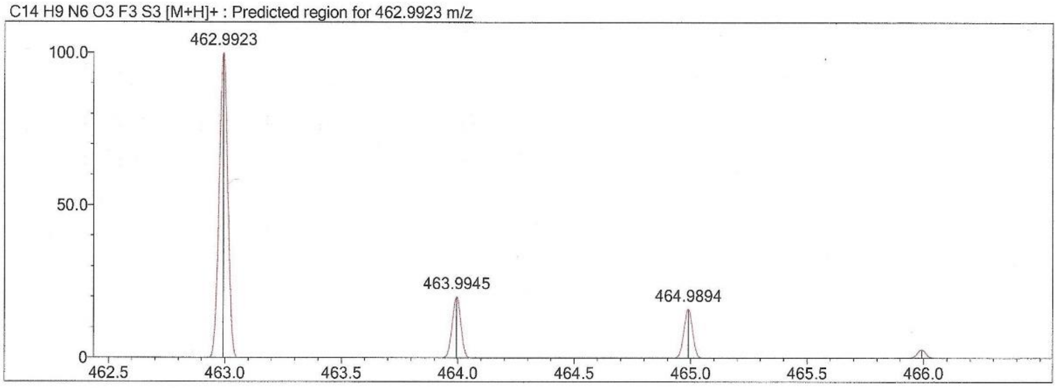

Rank	Score	Formula (M)	Ion	Meas. m/z	Pred. m/z	Df. (mDa)	Df. (ppm)	Iso	DBE
1	76.53	C14 H9 N6 O3 F3 S3	[M+H]+	462.9926	462.9923	0.3	0.65	76.53	12.0

IR Spectrum of Compound **3**

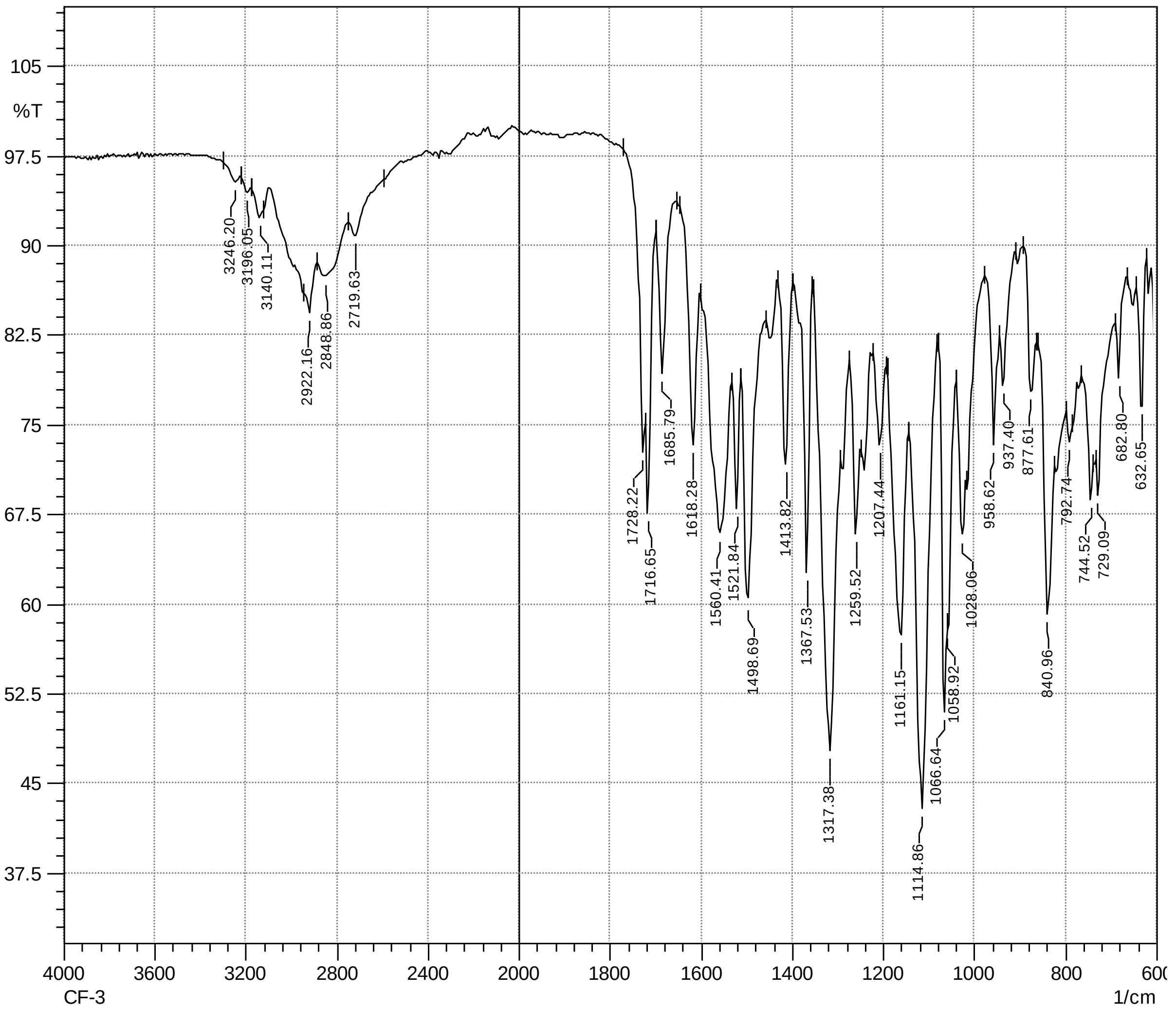

^{1}H NMR Spectrum of Compound **3**

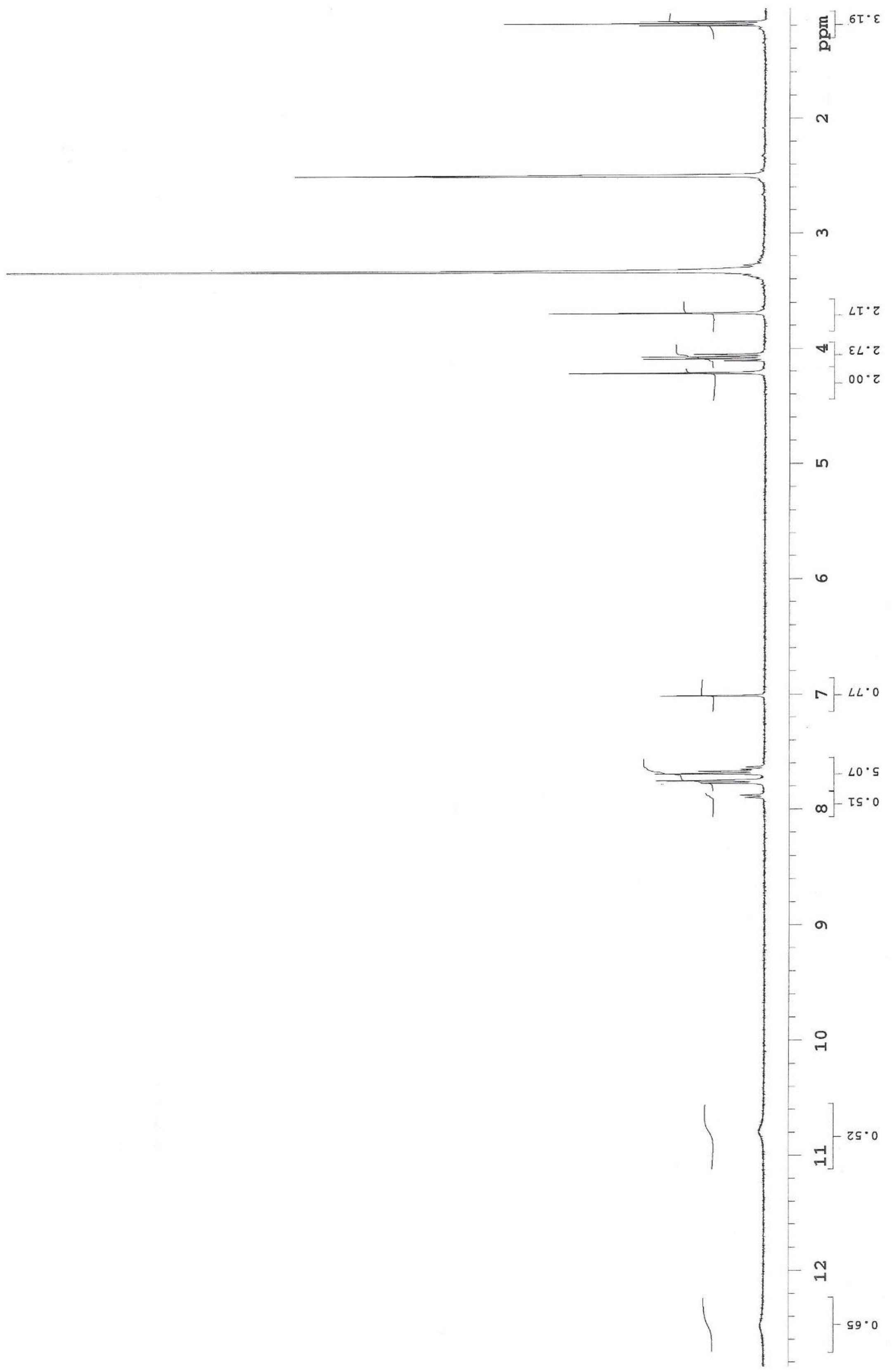

^{13}C NMR Spectrum of Compound **3**

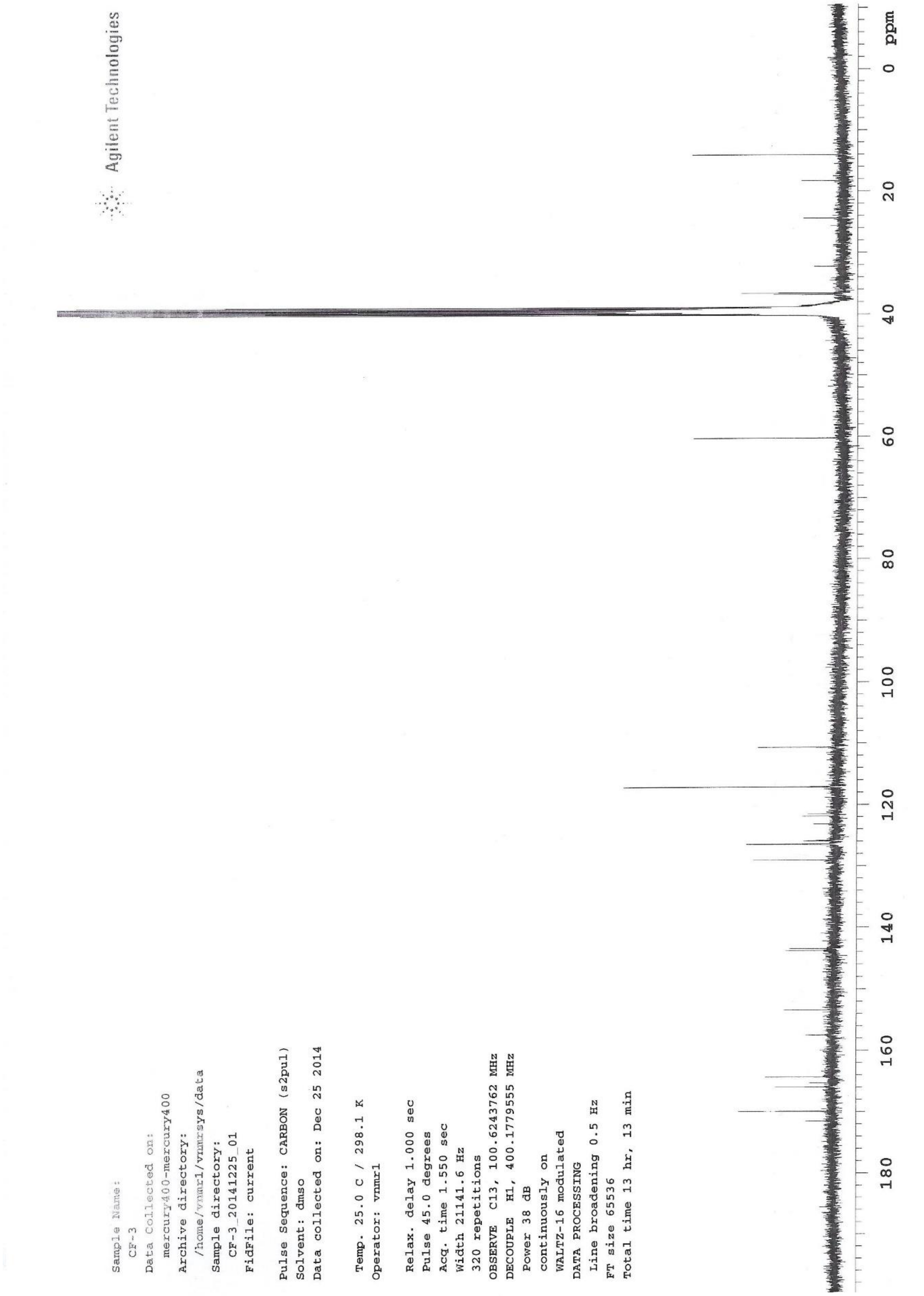

Mass Spectrum of Compound **3**

Formula Predictor Report - CF-3_14.lcd

Data File: C:\LabSolutions\Data\Analiz\mdaltintop\CF-3_14.lcd

Elmt	Val.	Min	Max	Elmt	Val.	Min	Max	Elmt	Val.	Min	Max	Elmt	Val.	Min	Max	Use Adduct
H	1	8	30	O	2	0	3	Cl	1	0	0	I	3	0	0	H
C	4	13	26	F	1	0	3	Br	1	0	0					
N	3	3	6	S	2	1	3	Ru	2	0	0					

Error Margin (ppm):	10	DBE Range:	0.0 - 12.0	Electron Ions:	both
HC Ratio:	unlimited	Apply N Rule:	no	Use MSn Info:	no
Max Isotopes:	3	Isotope RI (%):	1.00	Isotope Res:	10000
MSn Iso RI (%):	10.00	MSn Logic Mode:	AND	Max Results:	500

Event#: 1 MS(E+) Ret. Time : 6.747 Scan# : 1013

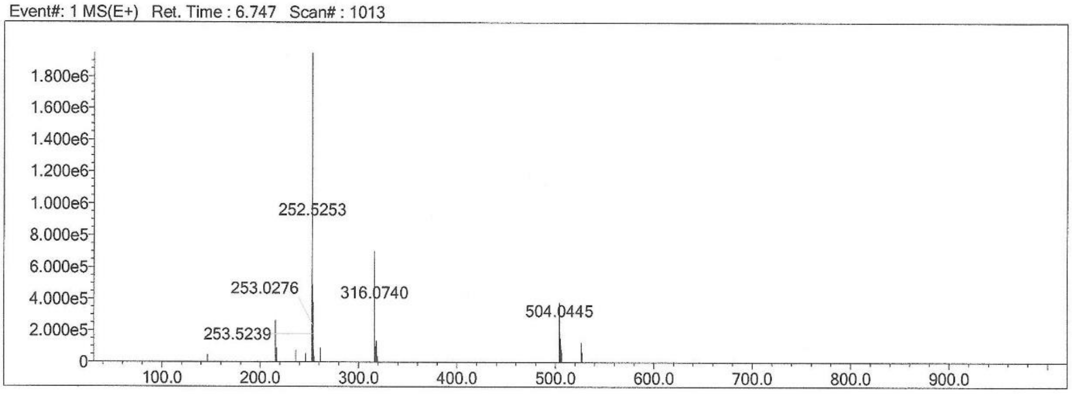

Measured region for 504.0445 m/z

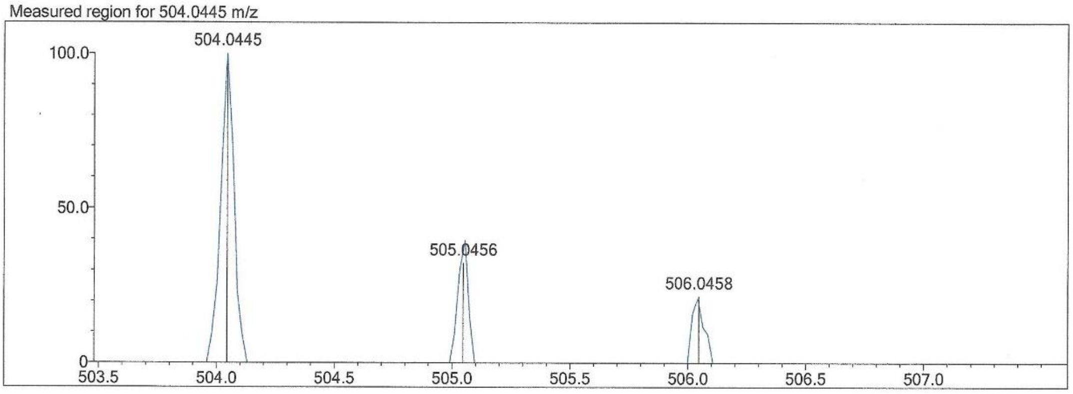

C18 H16 N5 O3 F3 S3 [M+H]+ : Predicted region for 504.0440 m/z

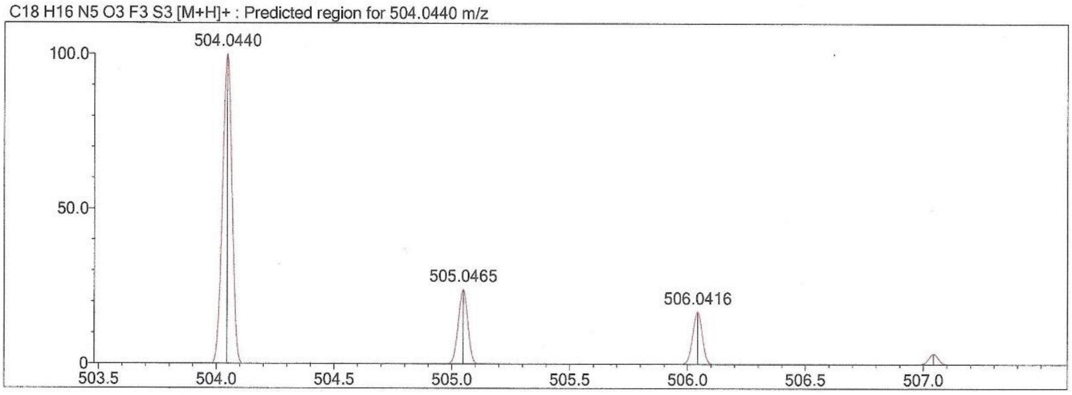

Rank	Score	Formula (M)	Ion	Meas. m/z	Pred. m/z	Df. (mDa)	Df. (ppm)	Iso	DBE
1	58.47	C18 H16 N5 O3 F3 S3	[M+H]+	504.0445	504.0440	0.5	0.99	58.47	12.0

IR Spectrum of Compound **4**

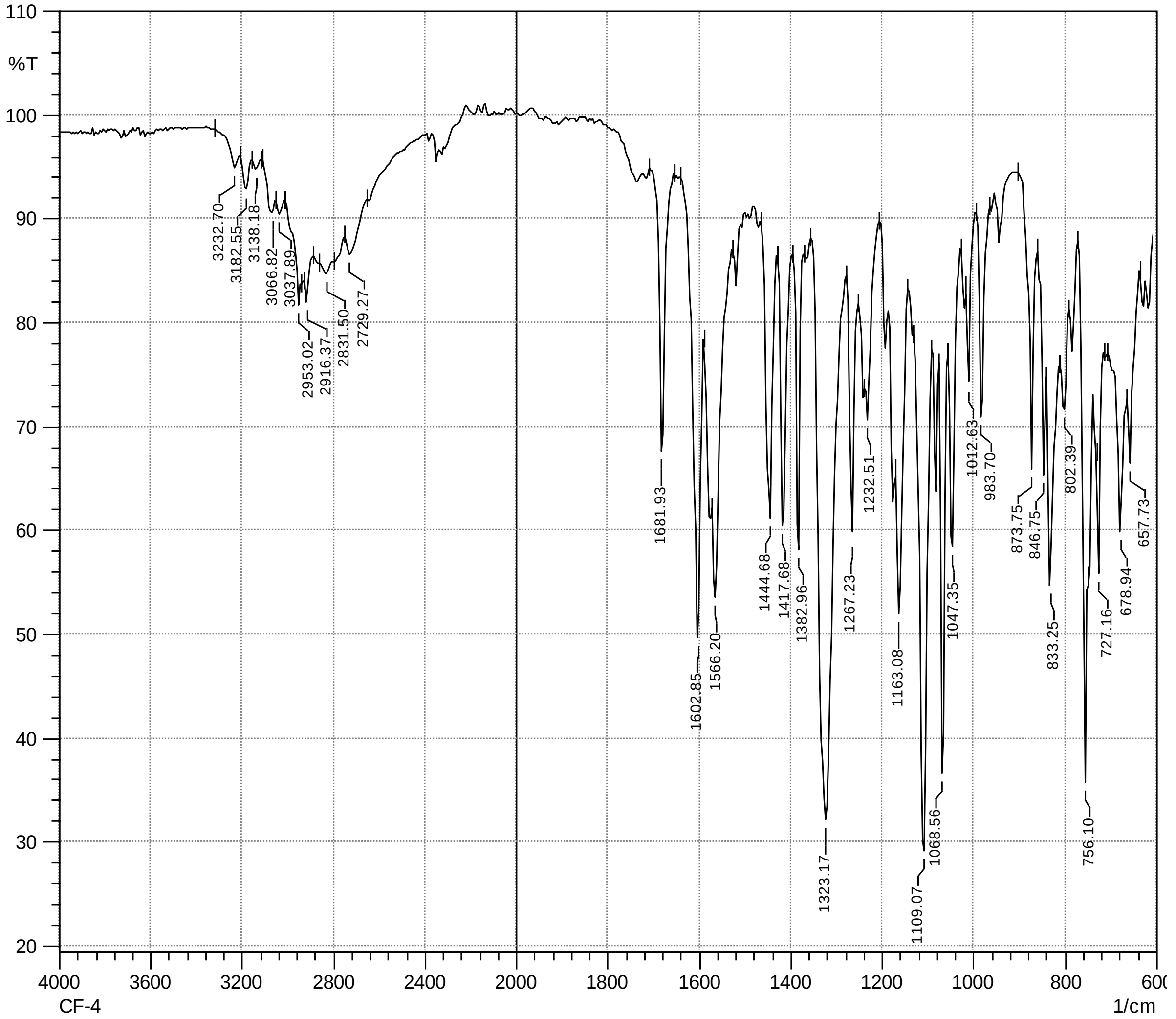

^{1}H NMR Spectrum of Compound **4**

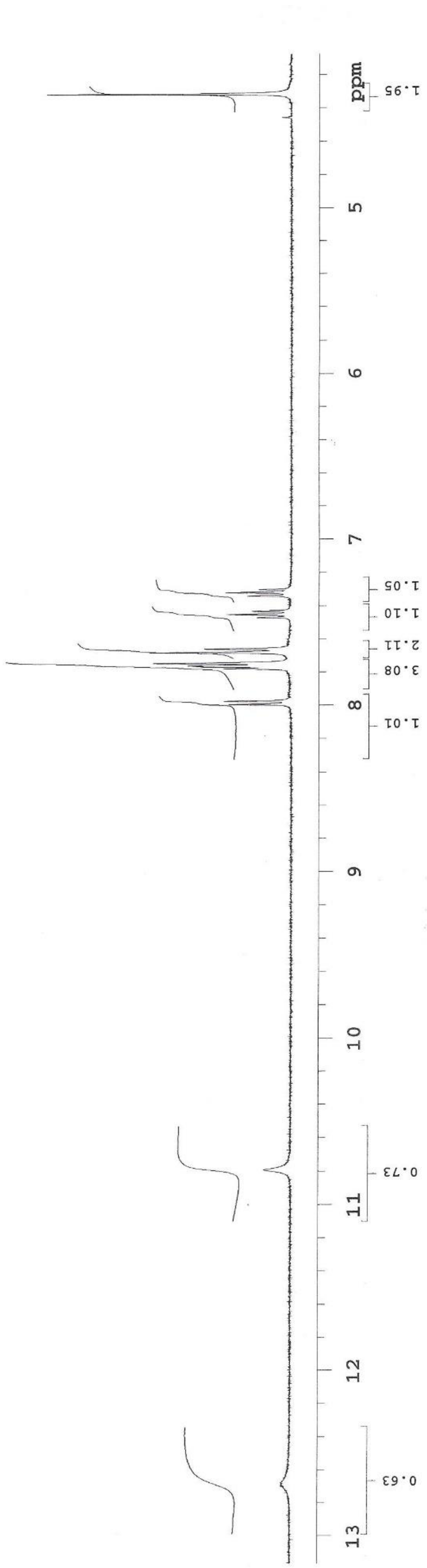

^{13}C NMR Spectrum of Compound **4**

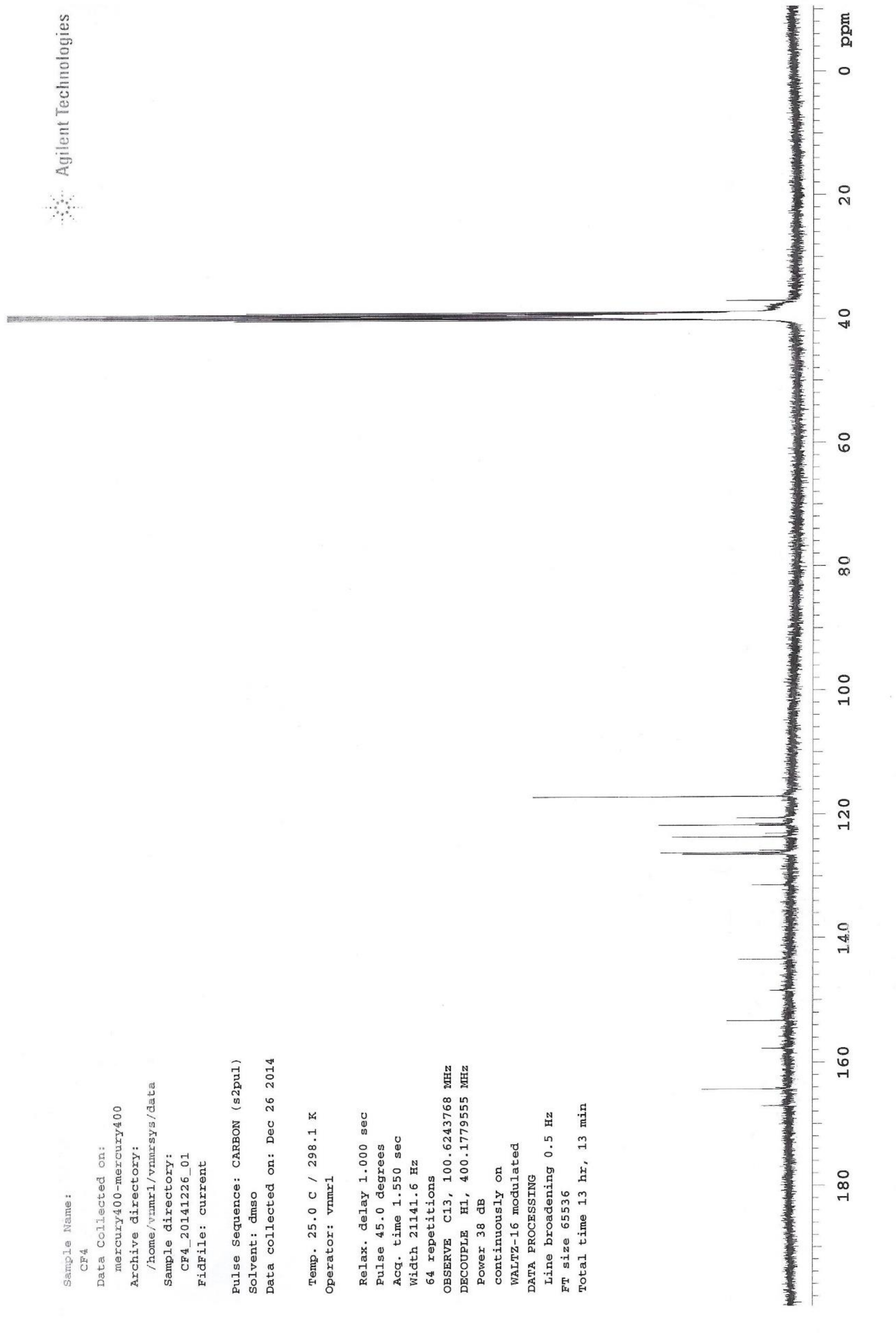

Mass Spectrum of Compound **4**

Formula Predictor Report - CF-4_1.lcd

Data File: C:\LabSolutions\Data\Analiz\mdaltintop\CF-4_1.lcd

Elmt	Val.	Min	Max	Elmt	Val.	Min	Max	Elmt	Val.	Min	Max	Elmt	Val.	Min	Max	Use Adduct
H	1	10	30	O	2	1	3	Cl	1	0	0	I	3	0	0	H
C	4	10	26	F	1	3	3	Br	1	0	0					
N	3	3	5	S	2	2	3	Ru	2	0	0					

Error Margin (ppm):	5	DBE Range:	14.0 - 20.0	Electron Ions:	both
HC Ratio:	unlimited	Apply N Rule:	no	Use MSn Info:	no
Max Isotopes:	3	Isotope RI (%):	1.00	Isotope Res:	10000
MSn Iso RI (%):	10.00	MSn Logic Mode:	AND	Max Results:	500

Event#: 1 MS(E+) Ret. Time : 8.307 Scan# : 1247

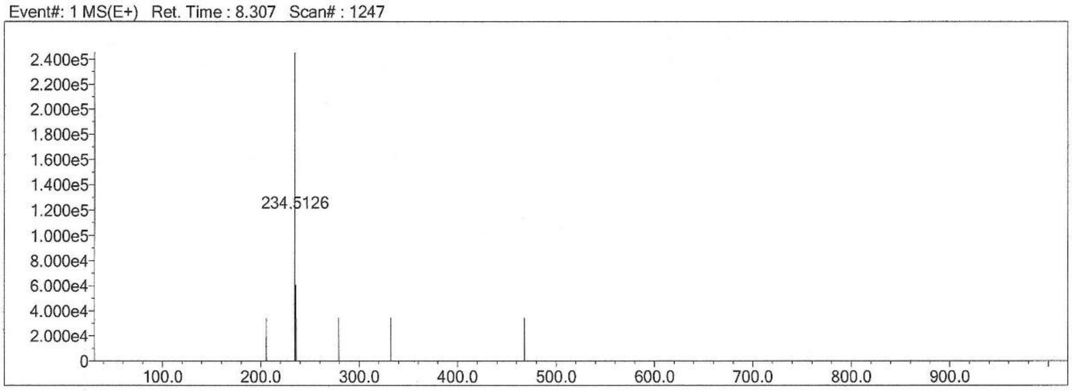

Measured region for 468.0213 m/z

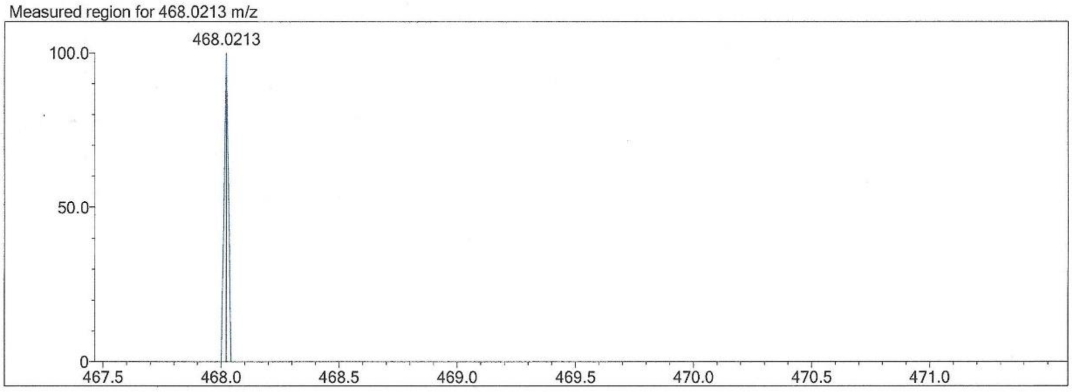

C18 H12 N5 O F3 S3 [M+H]+ : Predicted region for 468.0229 m/z

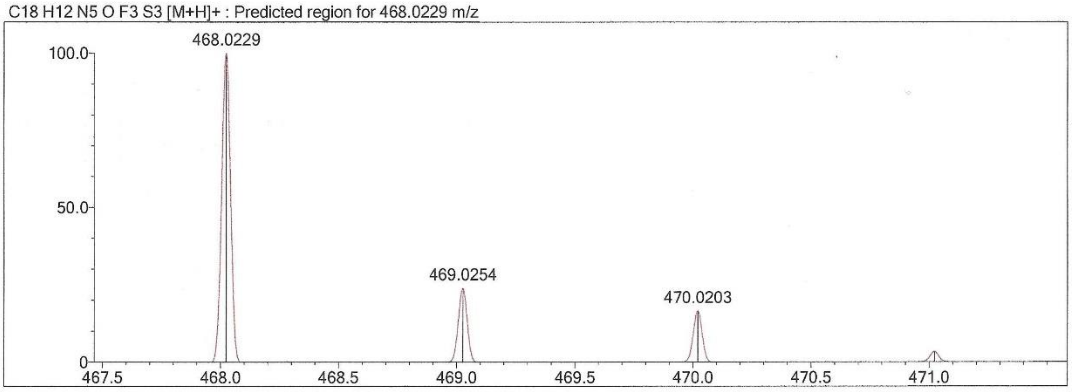

Rank	Score	Formula (M)	Ion	Meas. m/z	Pred. m/z	Df. (mDa)	Df. (ppm)	Iso	DBE
1	0.00	C18 H12 N5 O F3 S3	[M+H]+	468.0213	468.0229	-1.6	-3.42	0.00	14.0

IR Spectrum of Compound **5**

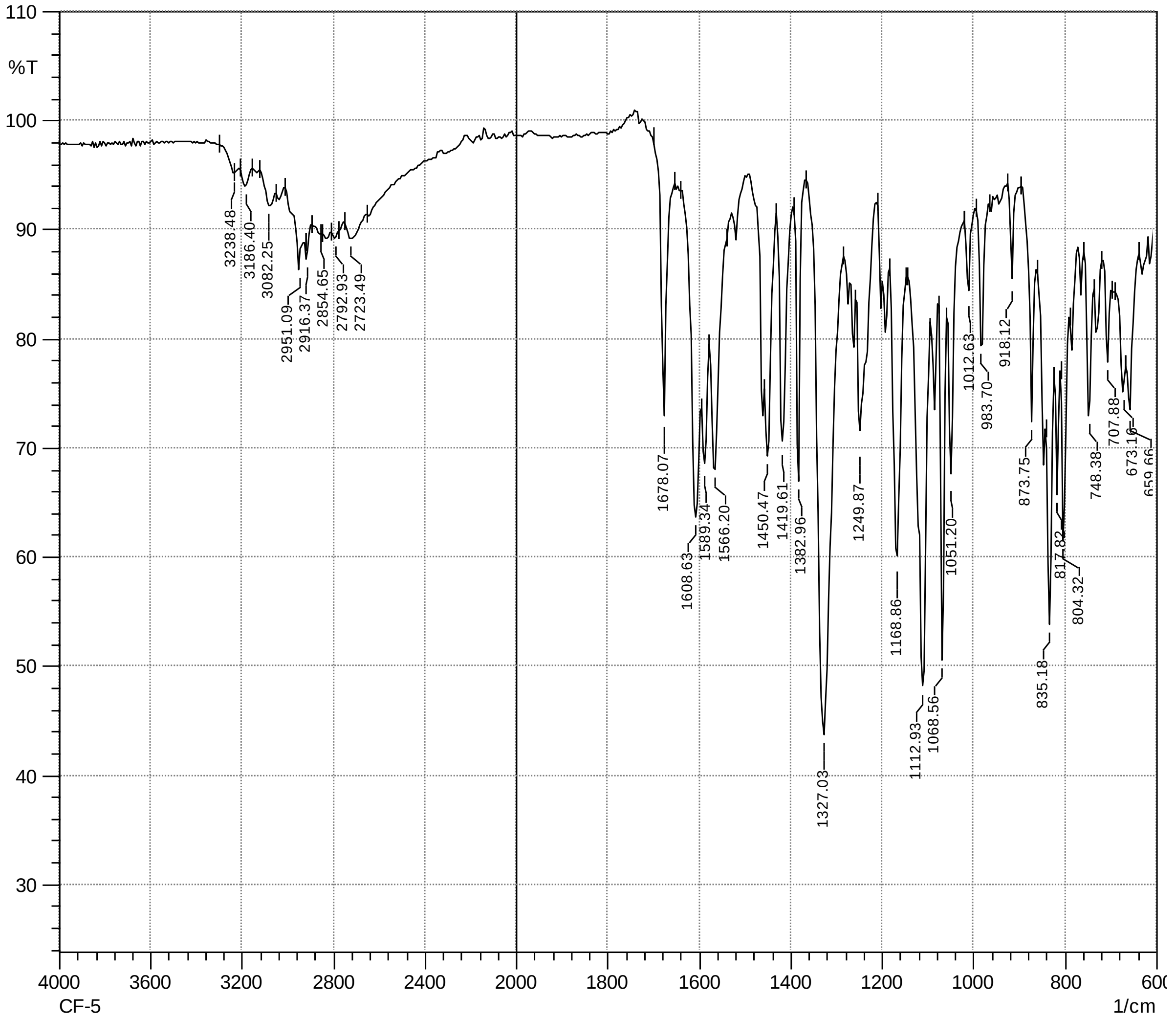

^{1}H NMR Spectrum of Compound **5**

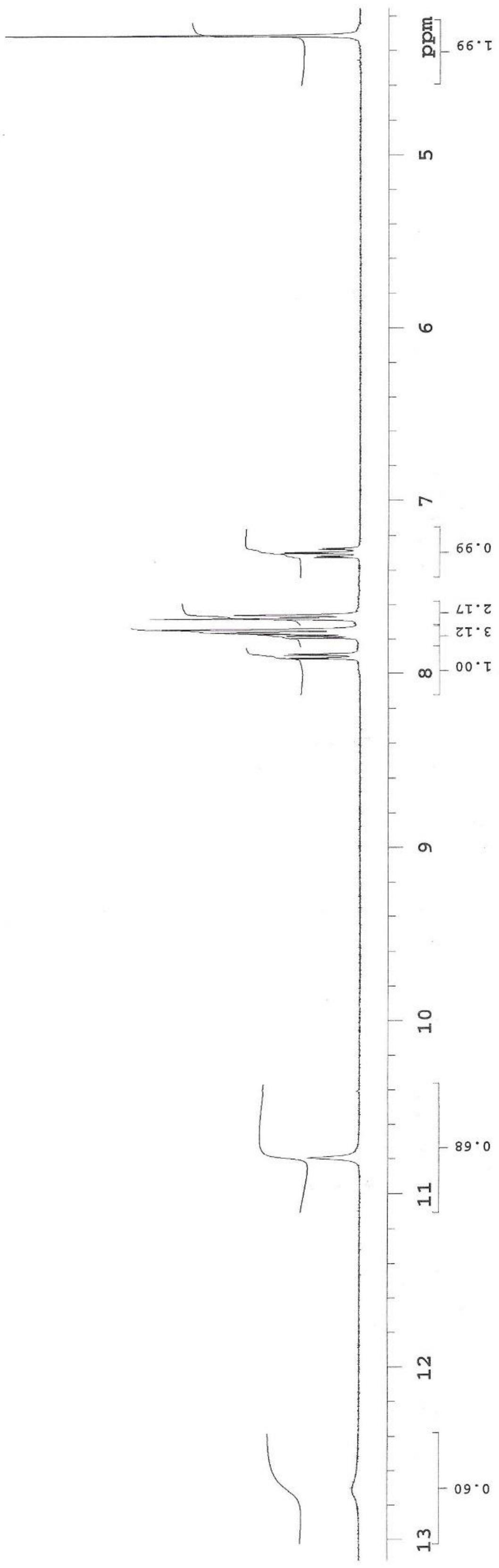

^{13}C NMR Spectrum of Compound **5**

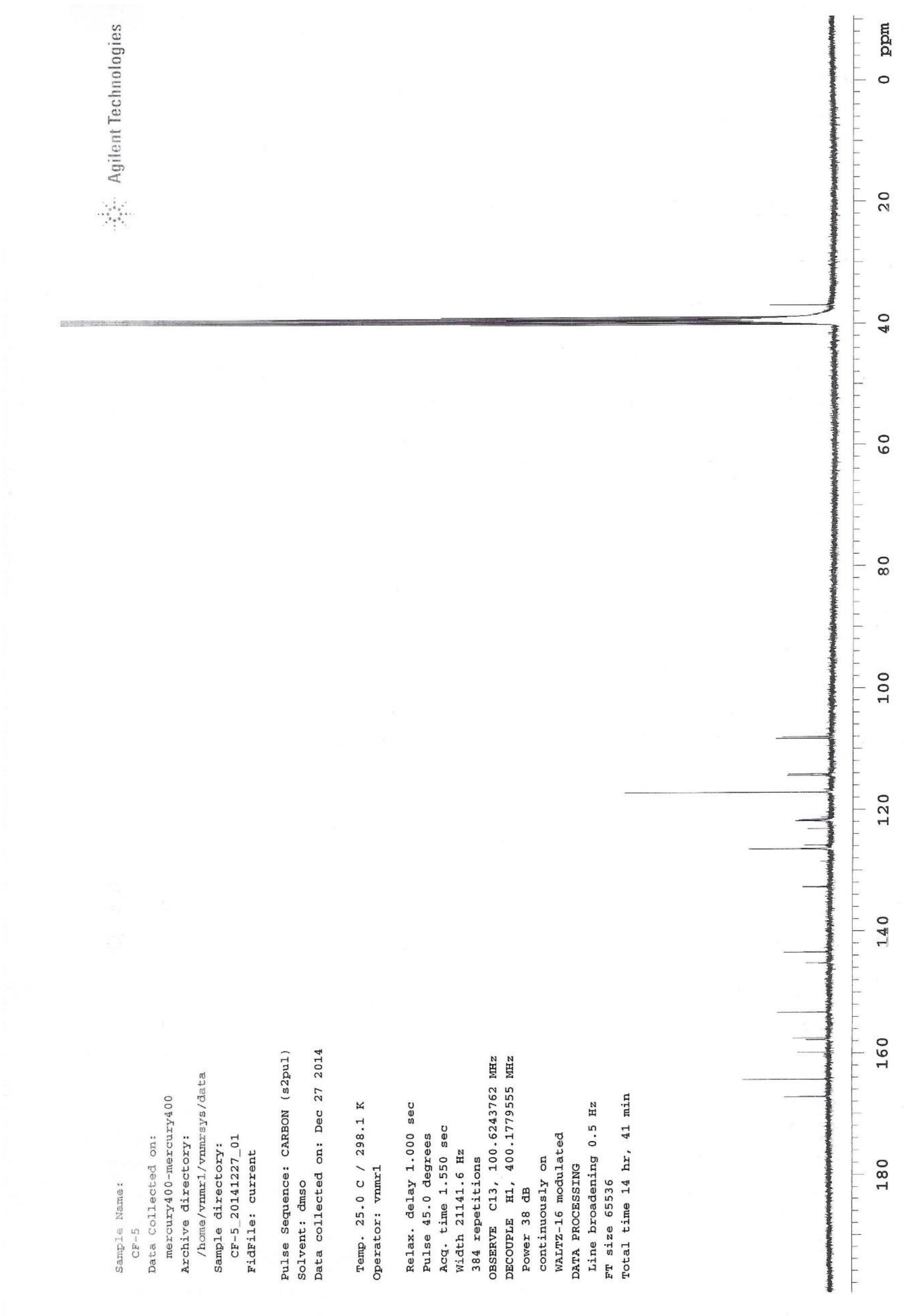

Mass Spectrum of Compound **5**

Formula Predictor Report - CF-5_1.lcd

Data File: C:\LabSolutions\Data\Analiz\mdaltintop\CF-5_1.lcd

Elmt	Val.	Min	Max	Elmt	Val.	Min	Max	Elmt	Val.	Min	Max	Elmt	Val.	Min	Max	Use Adduct
H	1	10	30	O	2	1	3	Cl	1	0	0	I	3	0	0	H
C	4	10	26	F	1	3	4	Br	1	0	0					
N	3	3	5	S	2	2	3	Ru	2	0	0					

Error Margin (ppm): 5
HC Ratio: unlimited
Max Isotopes: 3
MSn Iso RI (%): 10.00

DBE Range: 14.0 - 20.0
Apply N Rule: no
Isotope RI (%): 1.00
MSn Logic Mode: AND

Electron Ions: both
Use MSn Info: no
Isotope Res: 10000
Max Results: 500

Event#: 1 MS(E+) Ret. Time : 7.267 Scan# : 1091

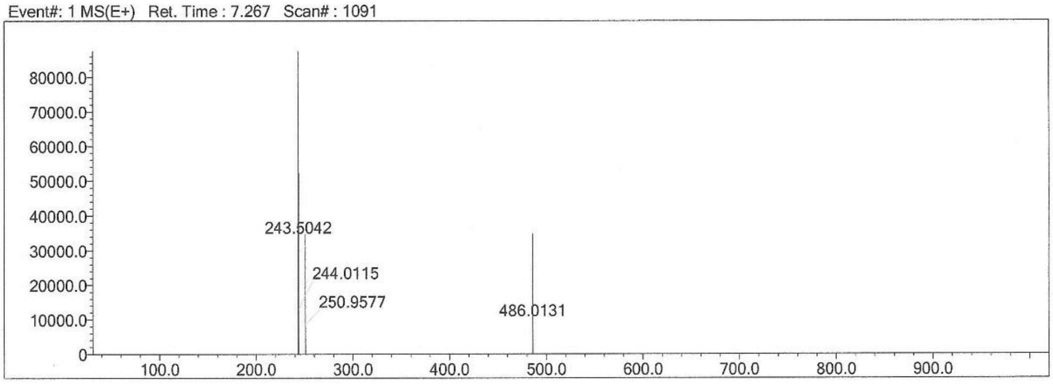

Measured region for 486.0131 m/z

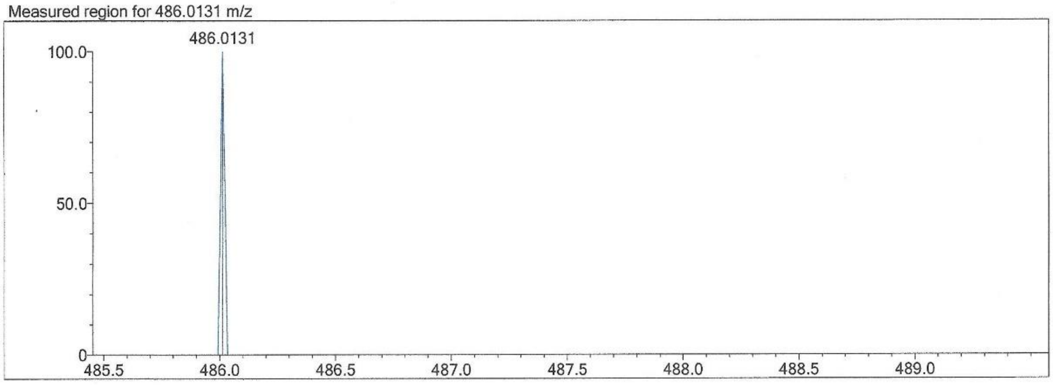

C18 H11 N5 O F4 S3 [M+H]+ : Predicted region for 486.0135 m/z

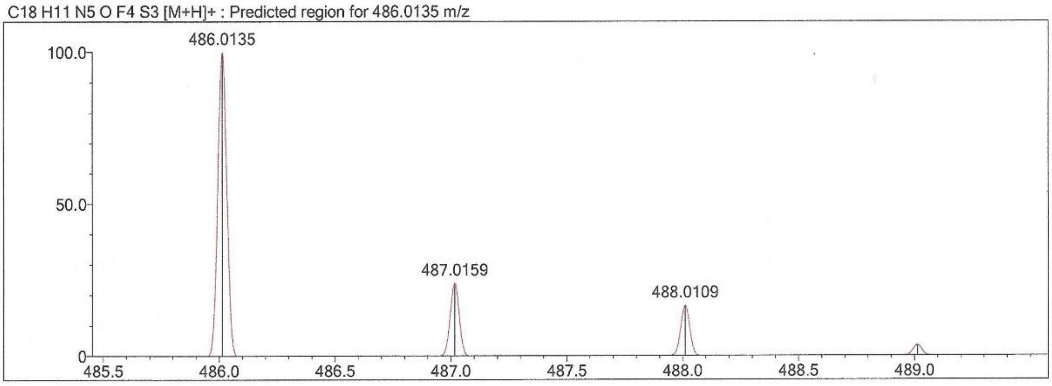

Rank	Score	Formula (M)	Ion	Meas. m/z	Pred. m/z	Df. (mDa)	Df. (ppm)	Iso	DBE
1	0.00	C18 H11 N5 O F4 S3	[M+H]+	486.0131	486.0135	-0.4	-0.82	0.00	14.0

IR Spectrum of Compound **6**

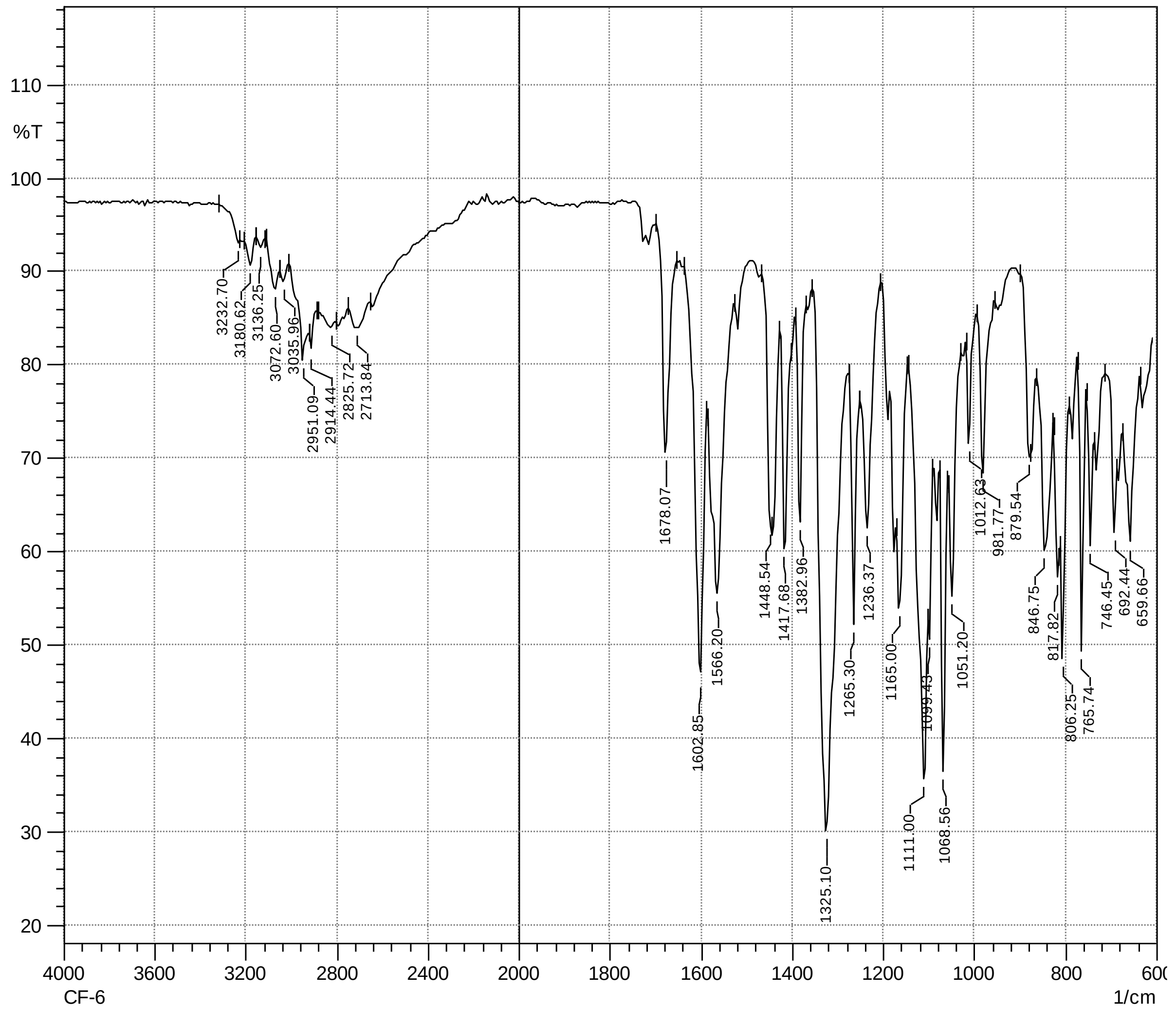

^{1}H NMR Spectrum of Compound **6**

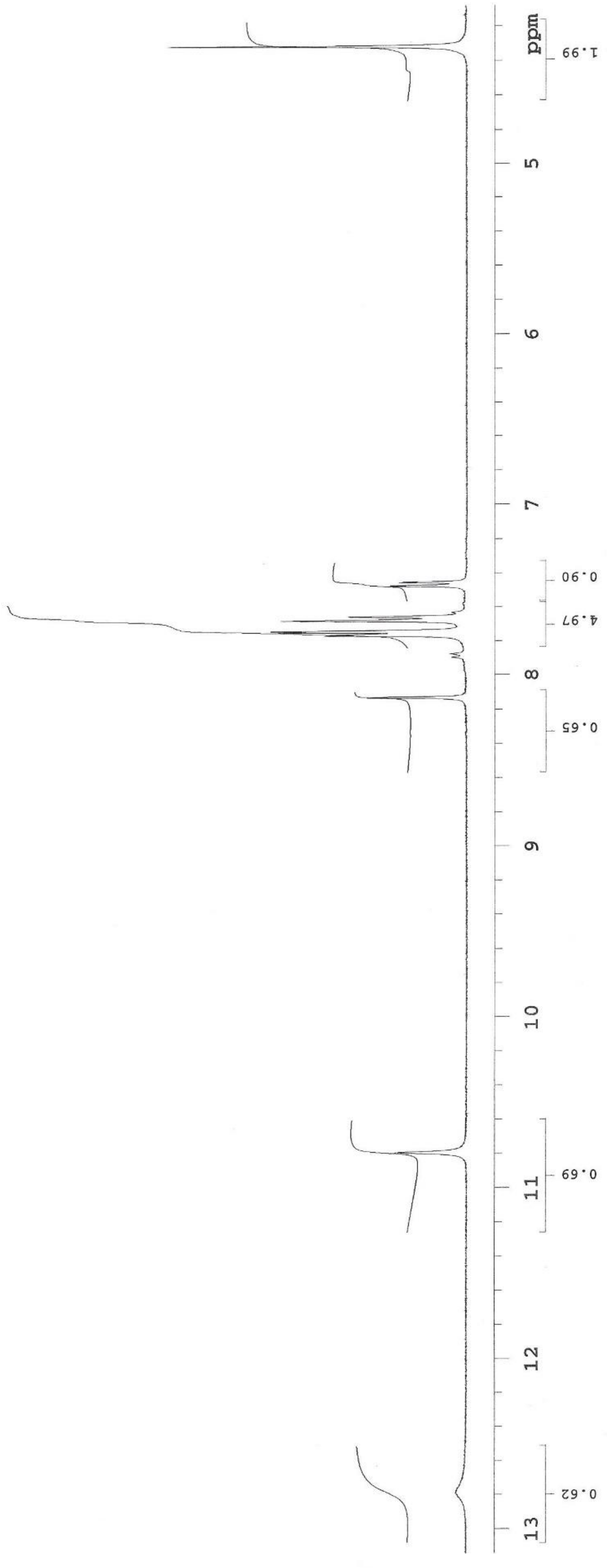

^{13}C NMR Spectrum of Compound **6**

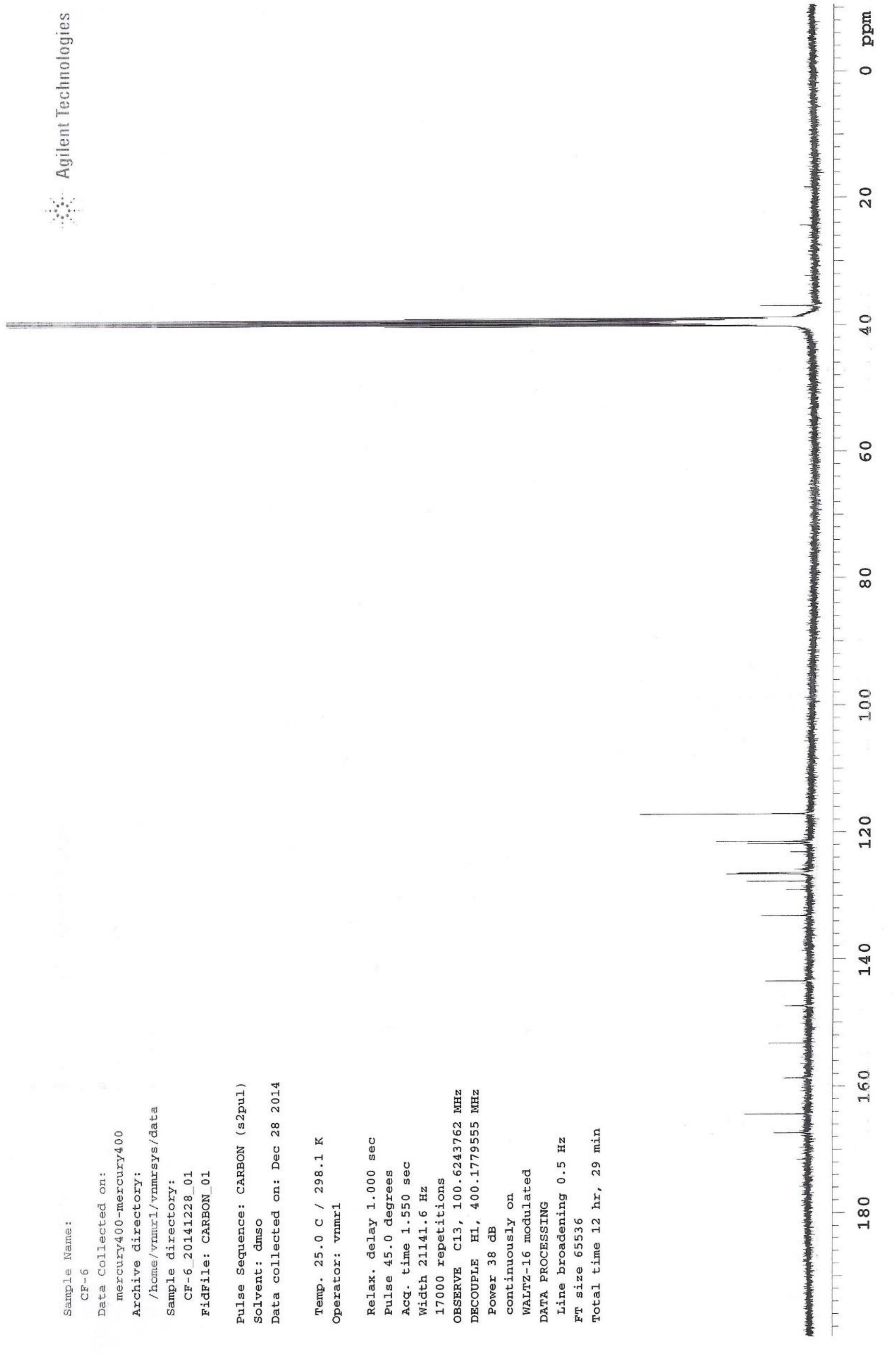

Mass Spectrum of Compound **6**

Formula Predictor Report - CF-6_1.lcd — Page 1 of 1

Data File: C:\LabSolutions\Data\Analiz\mdaltintop\CF-6_1.lcd

Elmt	Val.	Min	Max	Elmt	Val.	Min	Max	Elmt	Val.	Min	Max	Elmt	Val.	Min	Max	Use Adduct
H	1	10	30	O	2	1	3	Cl	1	0	1	I	3	0	0	H
C	4	10	26	F	1	3	4	Br	1	0	0					
N	3	3	5	S	2	2	3	Ru	2	0	0					

Error Margin (ppm):	5	DBE Range:	14.0 - 20.0	Electron Ions:	both
HC Ratio:	unlimited	Apply N Rule:	no	Use MSn Info:	no
Max Isotopes:	3	Isotope RI (%):	1.00	Isotope Res:	10000
MSn Iso RI (%):	10.00	MSn Logic Mode:	AND	Max Results:	500

Event#: 1 MS(E+) Ret. Time : 7.813 Scan# : 1173

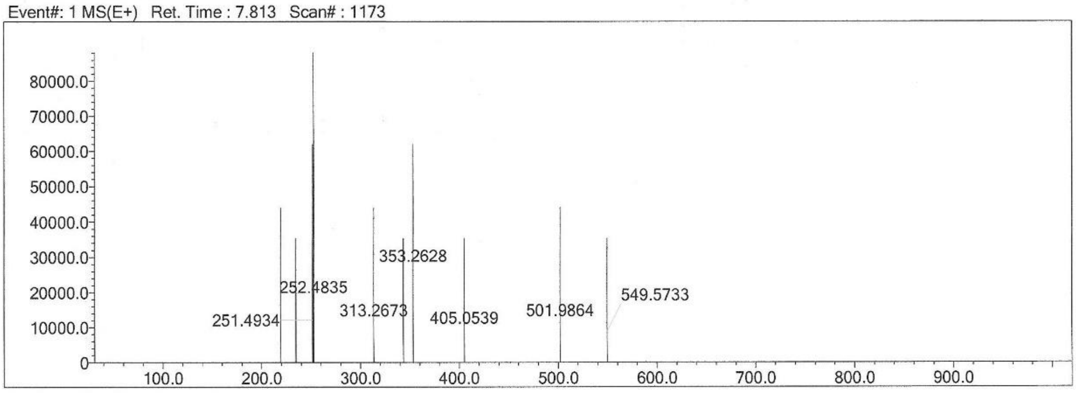

Measured region for 501.9864 m/z

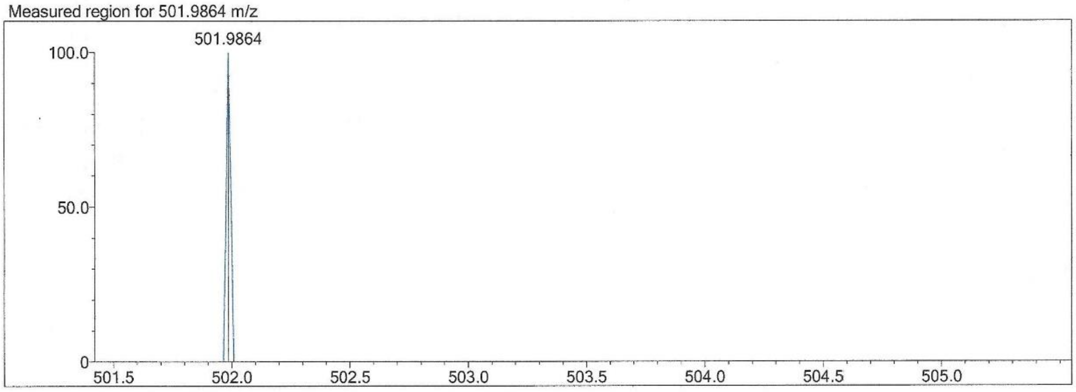

C18 H11 N5 O F3 S3 Cl [M+H]+ : Predicted region for 501.9839 m/z

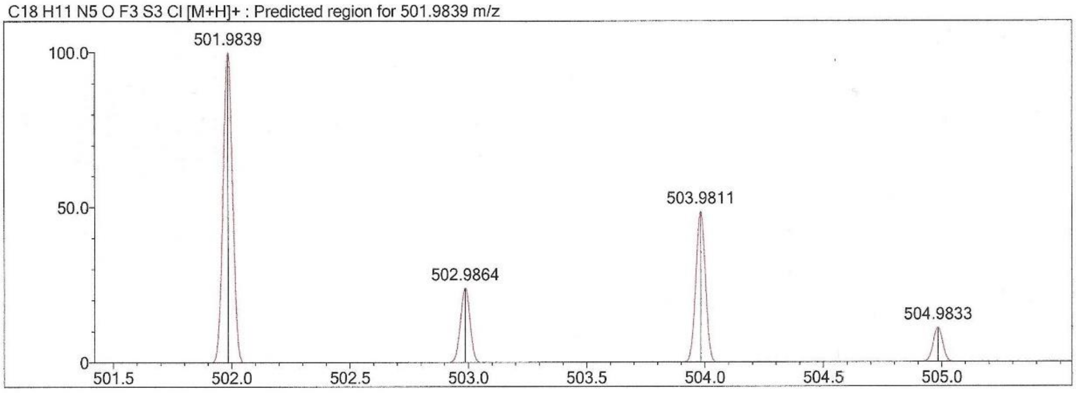

Rank	Score	Formula (M)	Ion	Meas. m/z	Pred. m/z	Df. (mDa)	Df. (ppm)	Iso	DBE
1	0.00	C18 H11 N5 O F3 S3 Cl	[M+H]+	501.9864	501.9839	2.5	4.98	0.00	14.0

IR Spectrum of Compound **7**

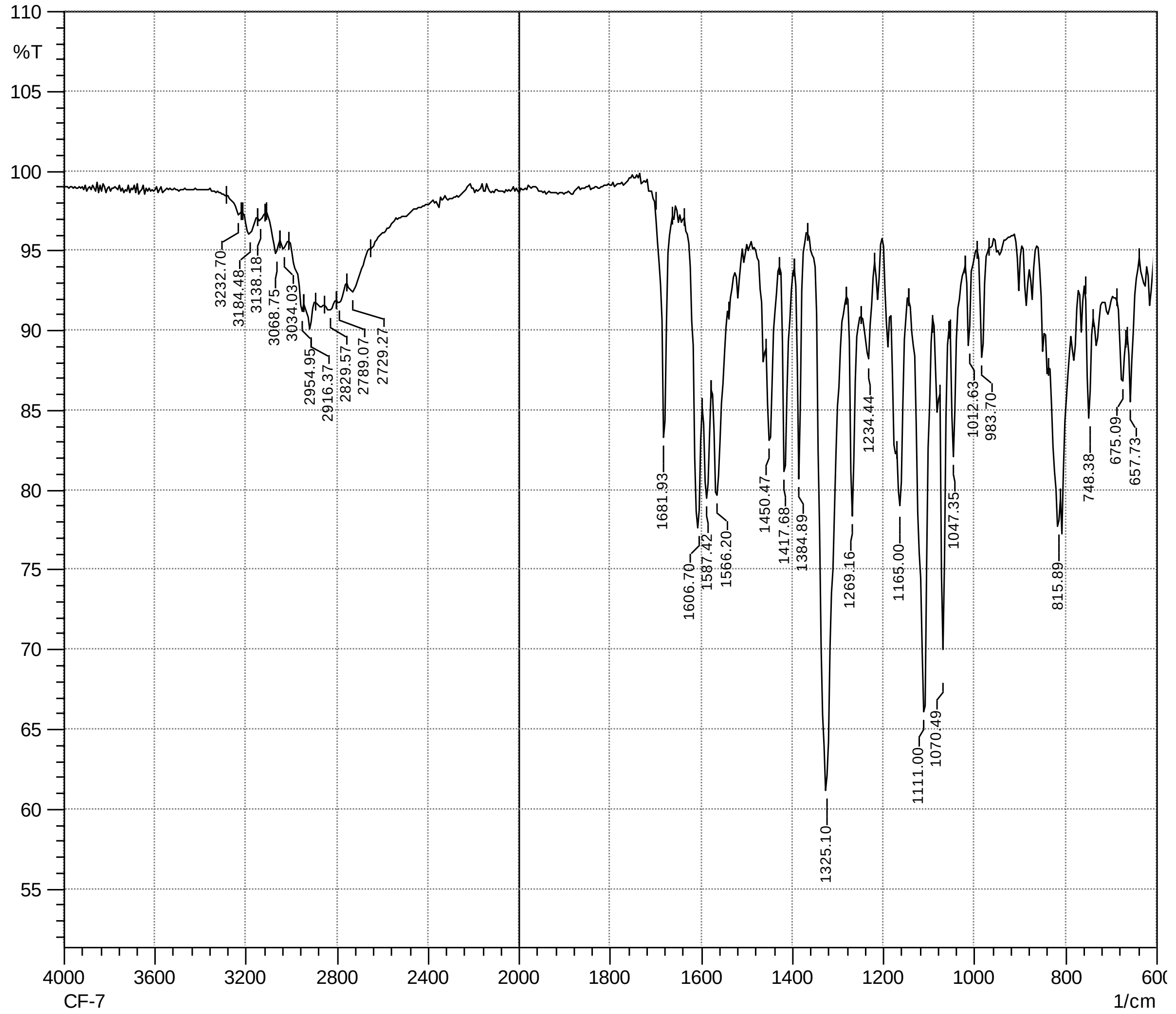

^{1}H NMR Spectrum of Compound **7**

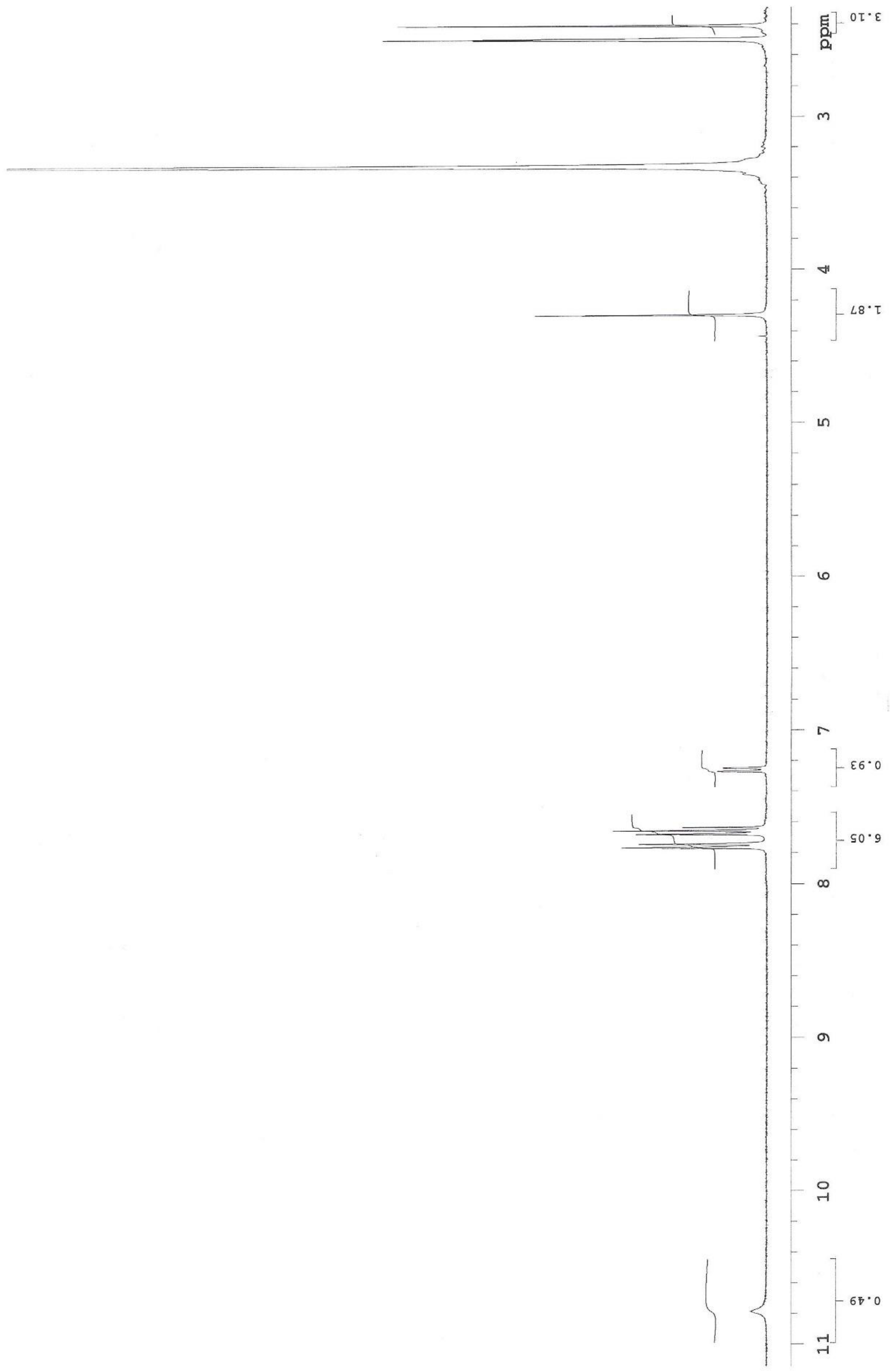

^{13}C NMR Spectrum of Compound **7**

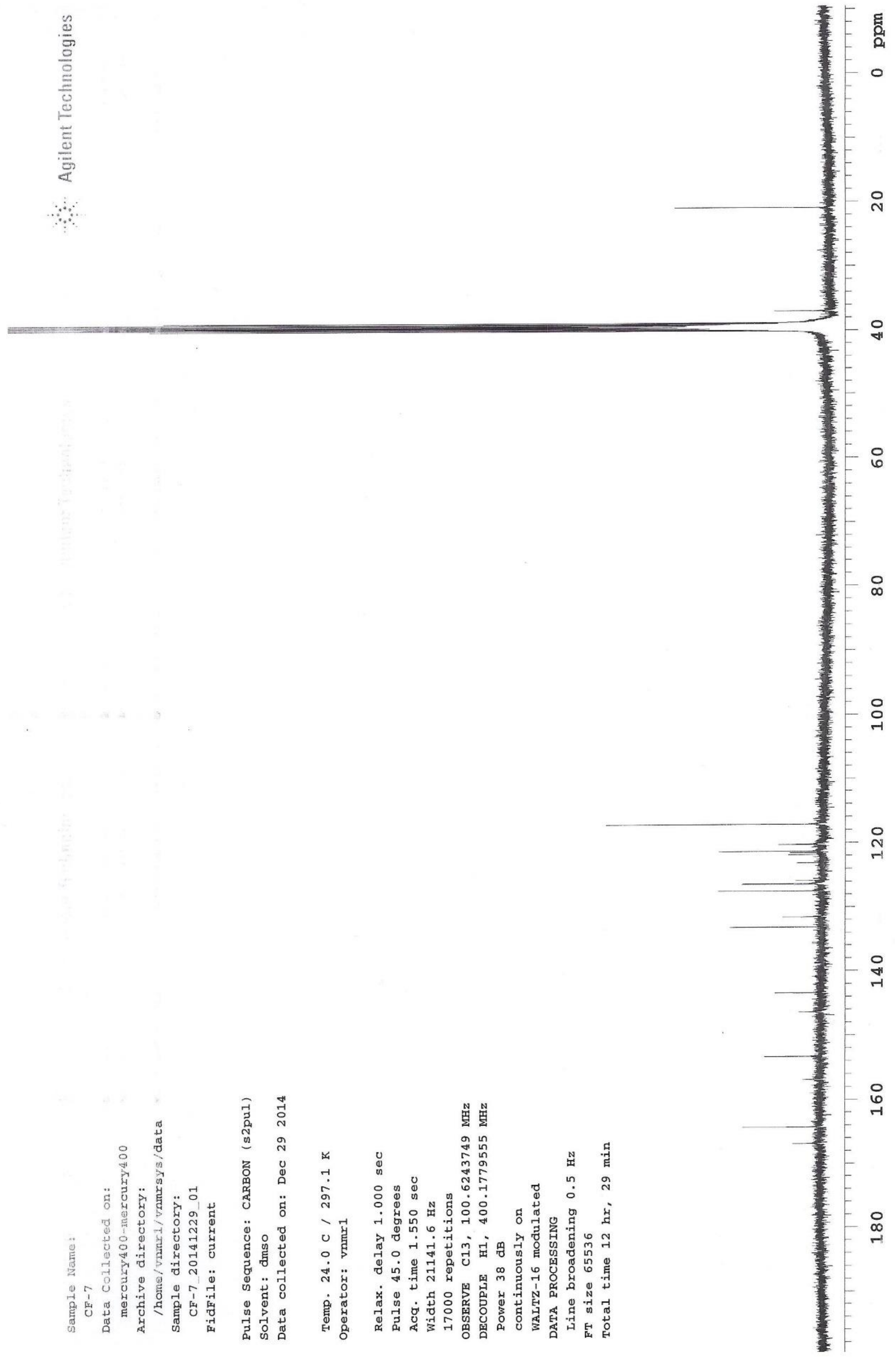

Mass Spectrum of Compound **7**

Formula Predictor Report - CF-7_1.lcd   Page 1 of 1

Data File: C:\LabSolutions\Data\Analiz\mdaltintop\CF-7_1.lcd

Elmt	Val.	Min	Max	Elmt	Val.	Min	Max	Elmt	Val.	Min	Max	Elmt	Val.	Min	Max	Use Adduct
H	1	10	30	O	2	1	1	Cl	1	0	0	I	3	0	0	H
C	4	10	26	F	1	3	4	Br	1	0	0					
N	3	3	5	S	2	3	3	Ru	2	0	0					

Error Margin (ppm):	5	DBE Range:	14.0 - 20.0	Electron Ions:	both
HC Ratio:	unlimited	Apply N Rule:	no	Use MSn Info:	no
Max Isotopes:	3	Isotope RI (%):	1.00	Isotope Res:	10000
MSn Iso RI (%):	10.00	MSn Logic Mode:	AND	Max Results:	500

Event#: 1 MS(E+) Ret. Time : 9.000 -> 9.000 Scan# : 1351 -> 1351

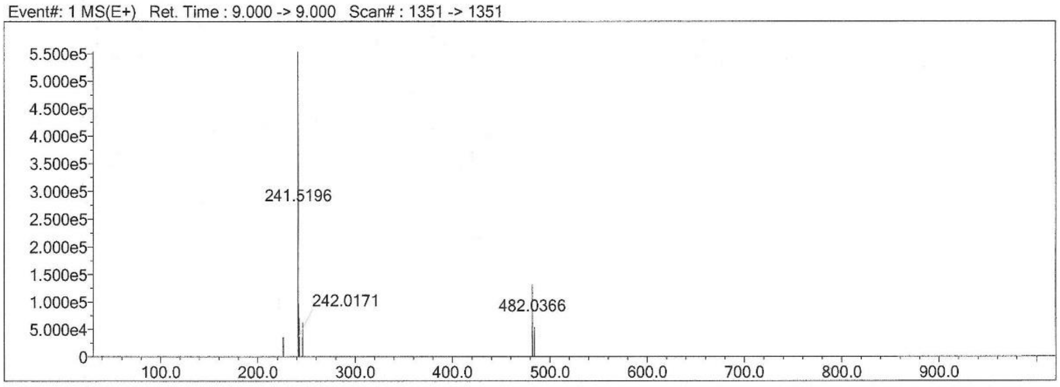

Measured region for 482.0366 m/z

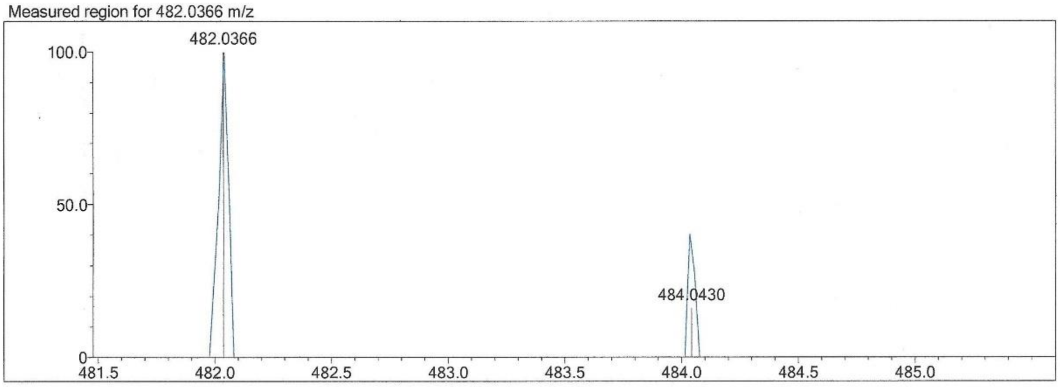

C19 H14 N5 O F3 S3 [M+H]+ : Predicted region for 482.0385 m/z

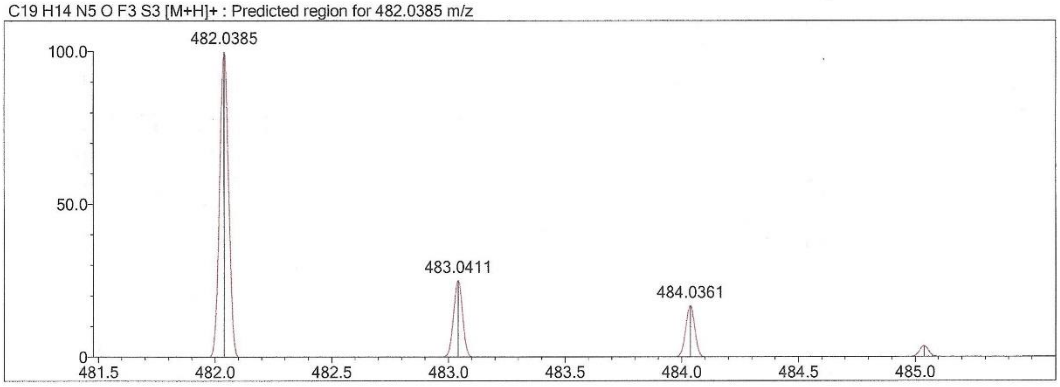

Rank	Score	Formula (M)	Ion	Meas. m/z	Pred. m/z	Df. (mDa)	Df. (ppm)	Iso	DBE
1	29.46	C19 H14 N5 O F3 S3	[M+H]+	482.0366	482.0385	-1.9	-3.94	31.80	14.0

IR Spectrum of Compound **8**

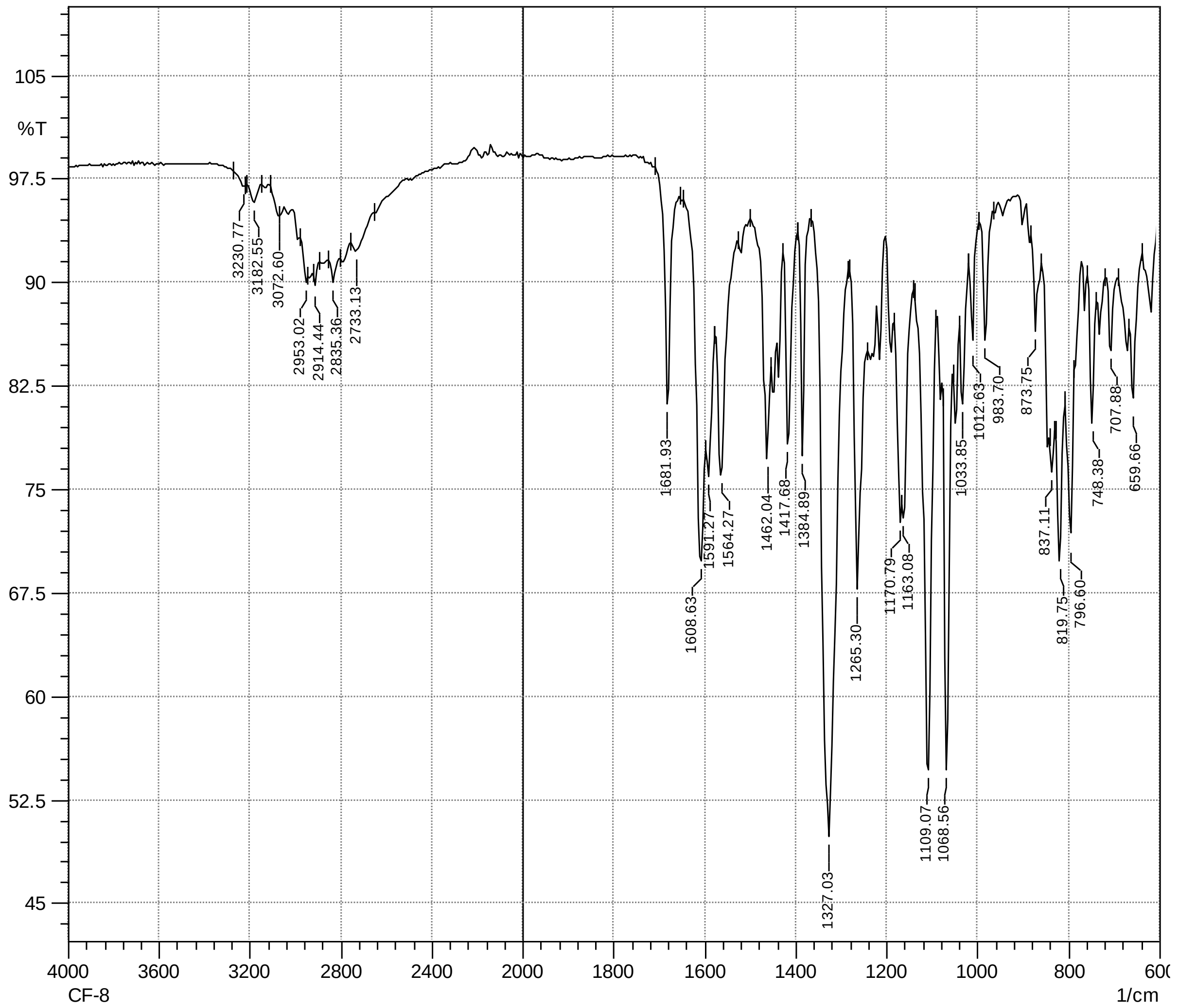

^{1}H NMR Spectrum of Compound **8**

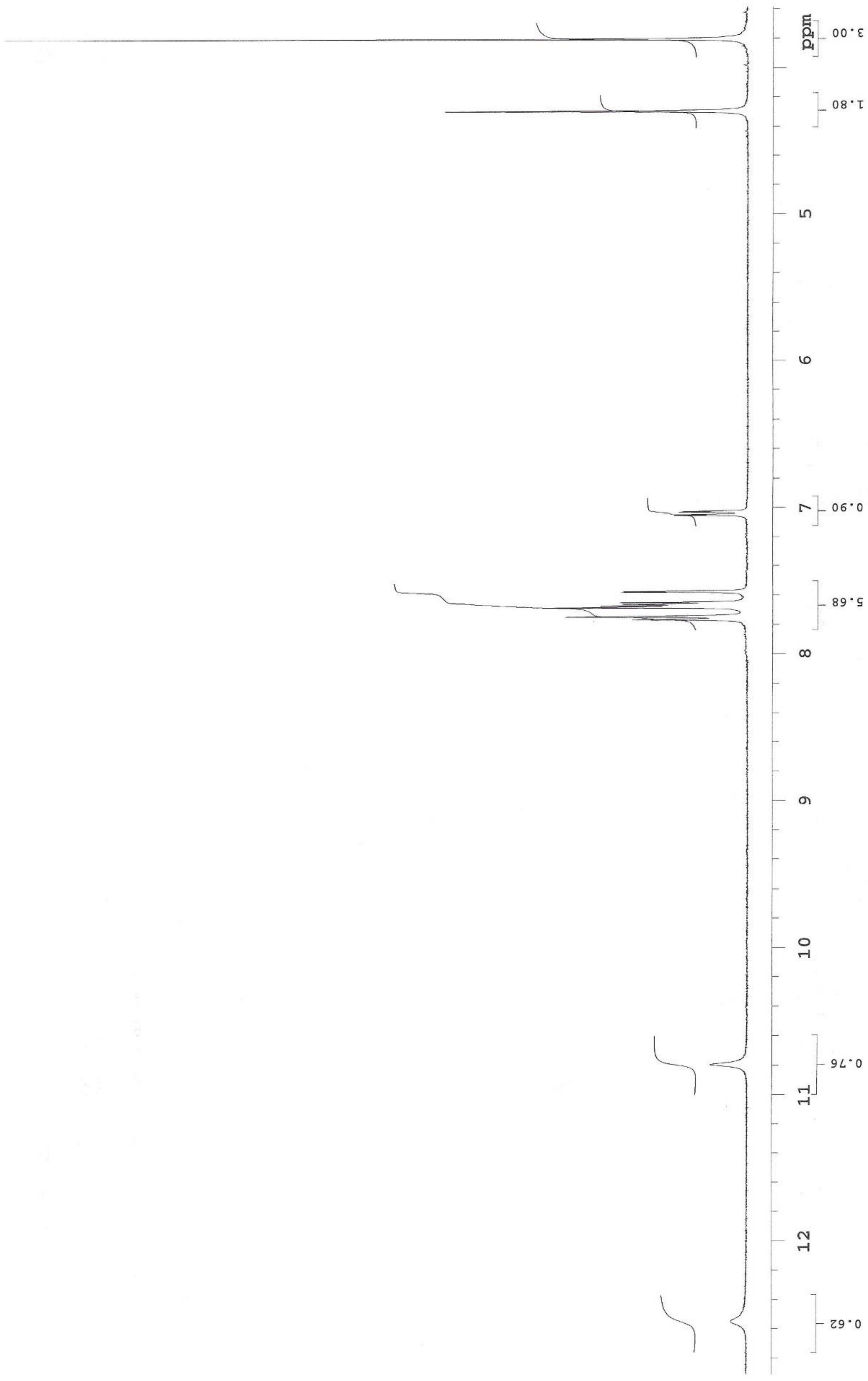

^{13}C NMR Spectrum of Compound **8**

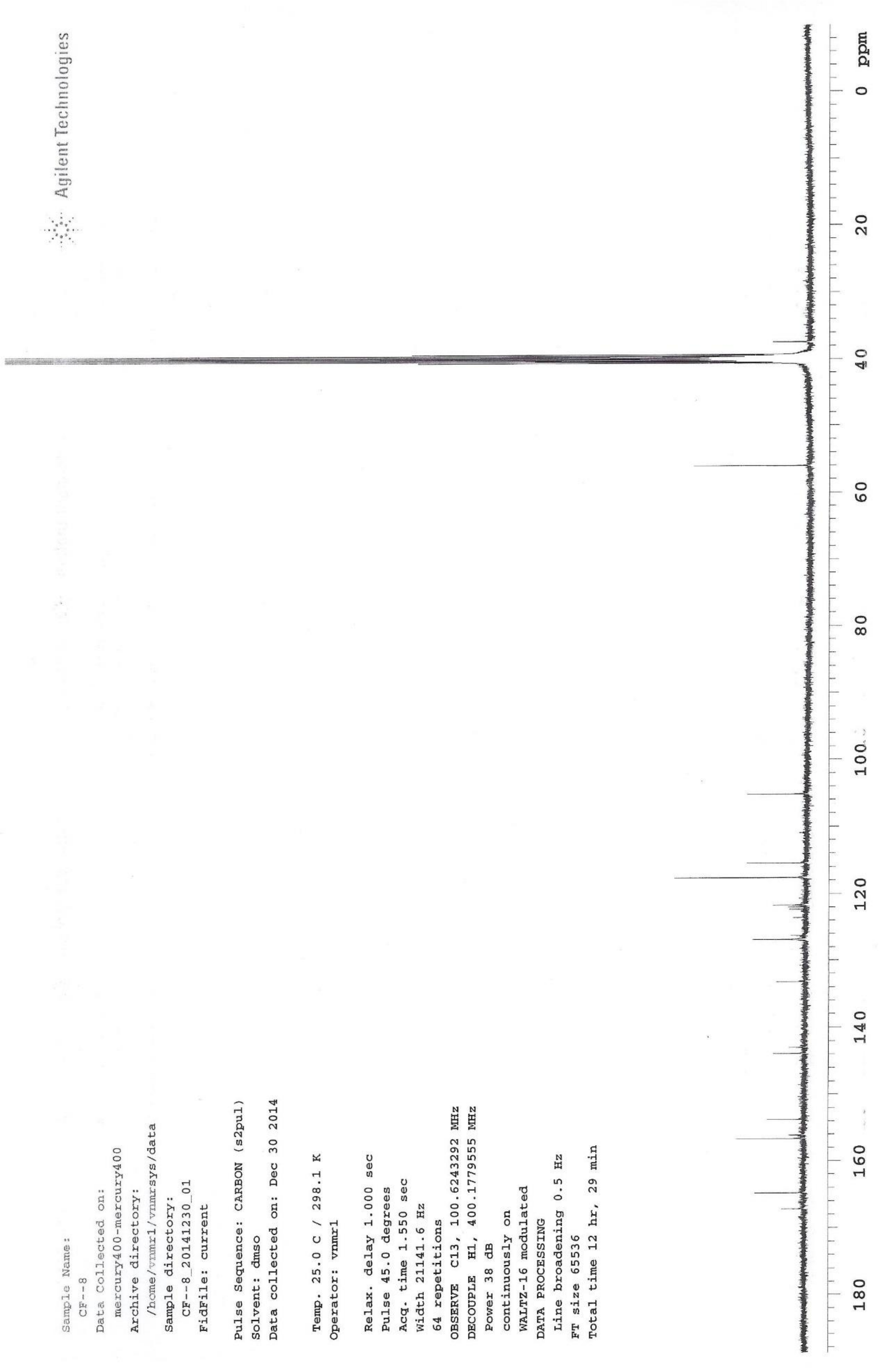

Mass Spectrum of Compound **8**

Formula Predictor Report - CF-8_19.lcd

Data File: C:\LabSolutions\Data\Analiz\mdaltintop\CF-8_19.lcd

Elmt	Val.	Min	Max	Elmt	Val.	Min	Max	Elmt	Val.	Min	Max	Elmt	Val.	Min	Max	Use Adduct
H	1	10	30	O	2	1	3	Cl	1	0	0	I	3	0	0	H
C	4	10	26	F	1	3	3	Br	1	0	0					
N	3	3	5	S	2	2	3	Ru	2	0	0					

Error Margin (ppm):	5	DBE Range:	14.0 - 20.0	Electron Ions:	both
HC Ratio:	unlimited	Apply N Rule:	no	Use MSn Info:	no
Max Isotopes:	3	Isotope RI (%):	1.00	Isotope Res:	10000
MSn Iso RI (%):	10.00	MSn Logic Mode:	AND	Max Results:	500

Event#: 1 MS(E+) Ret. Time : 7.067 Scan# : 1061

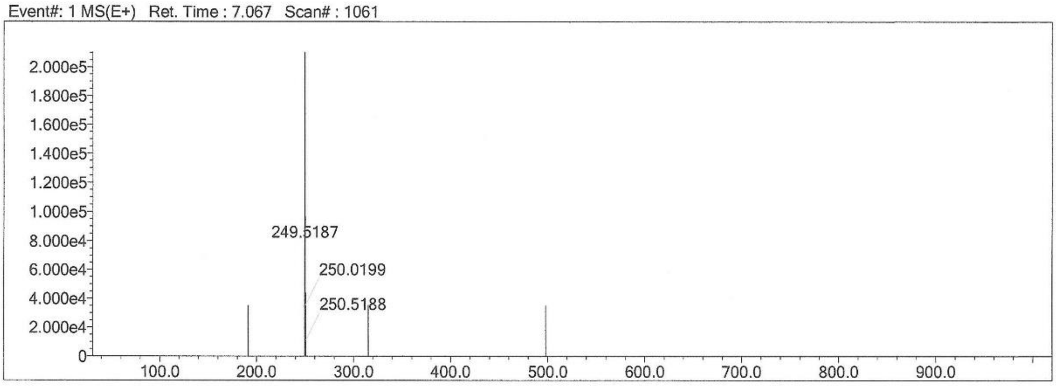

Measured region for 498.0328 m/z

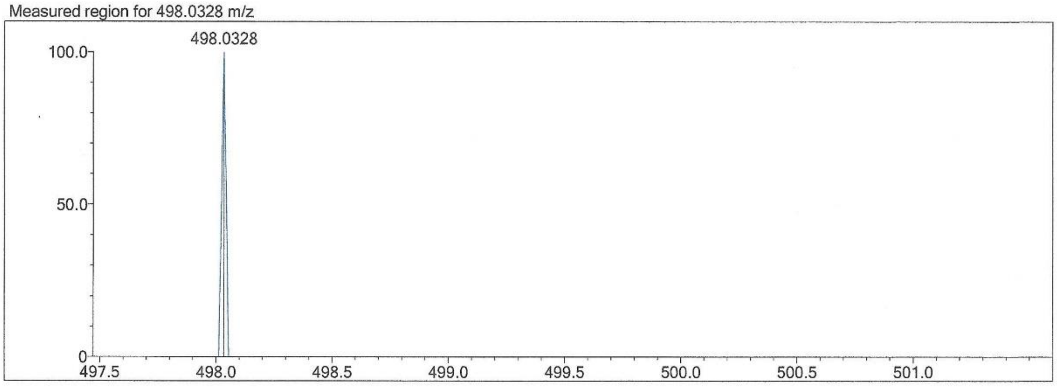

C19 H14 N5 O2 F3 S3 [M+H]+ : Predicted region for 498.0334 m/z

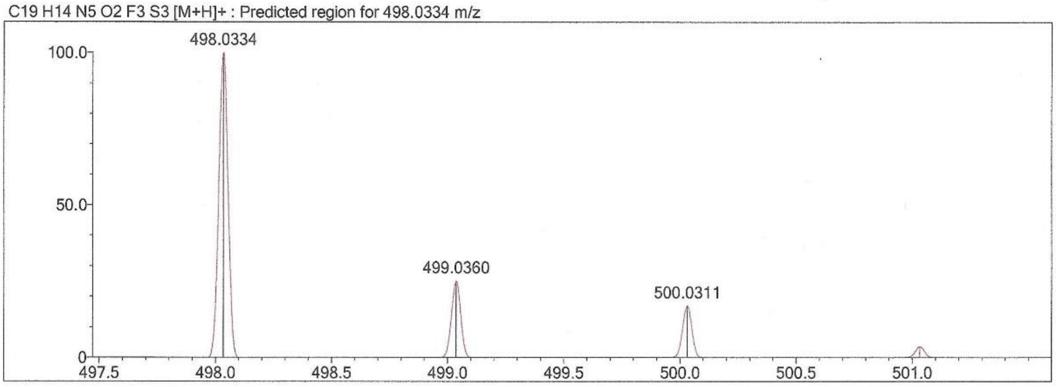

Rank	Score	Formula (M)	Ion	Meas. m/z	Pred. m/z	Df. (mDa)	Df. (ppm)	Iso	DBE
1	0.00	C19 H14 N5 O2 F3 S3	[M+H]+	498.0328	498.0334	-0.6	-1.20	0.00	14.0

IR Spectrum of Compound **9**

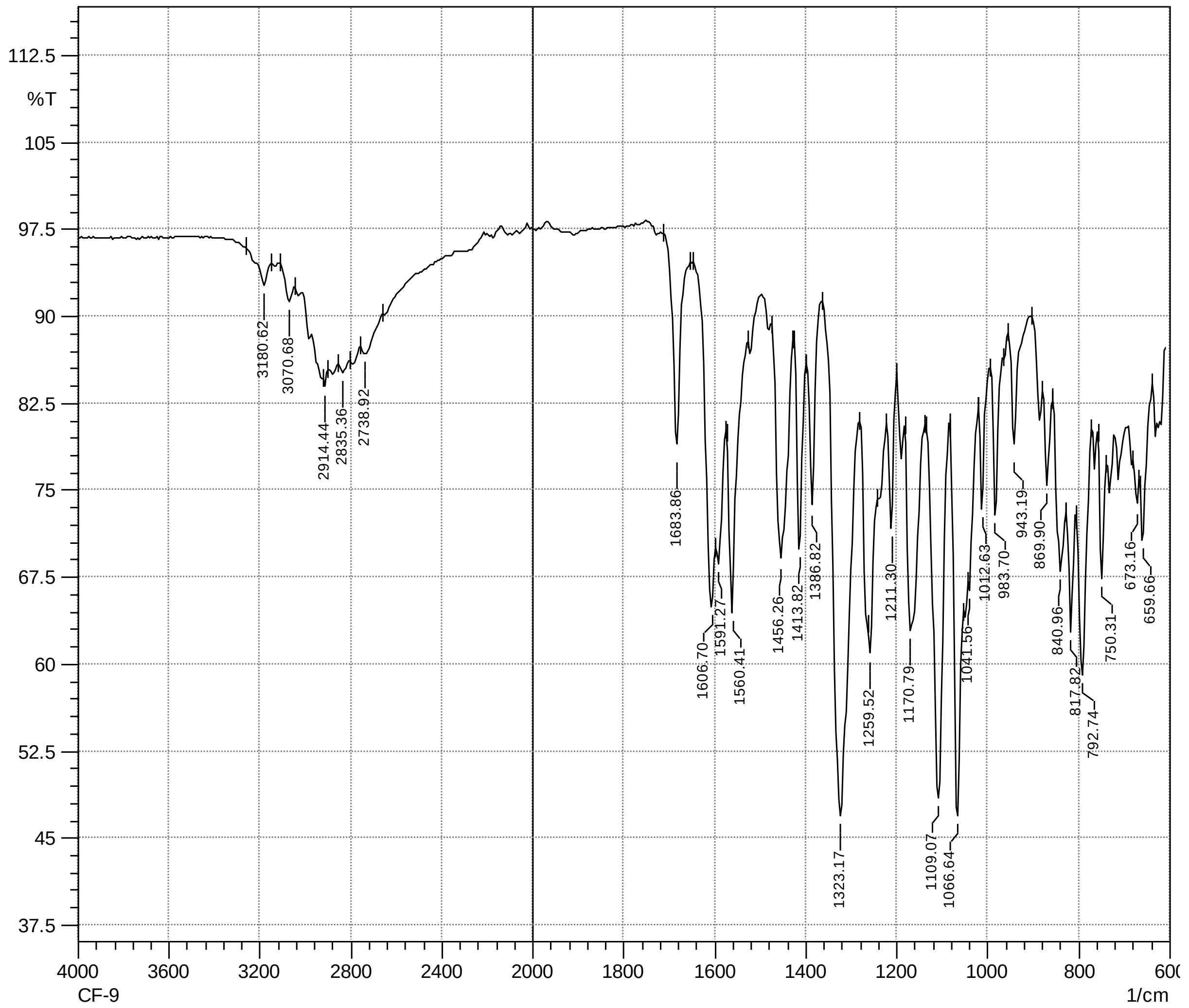

^{1}H NMR Spectrum of Compound **9**

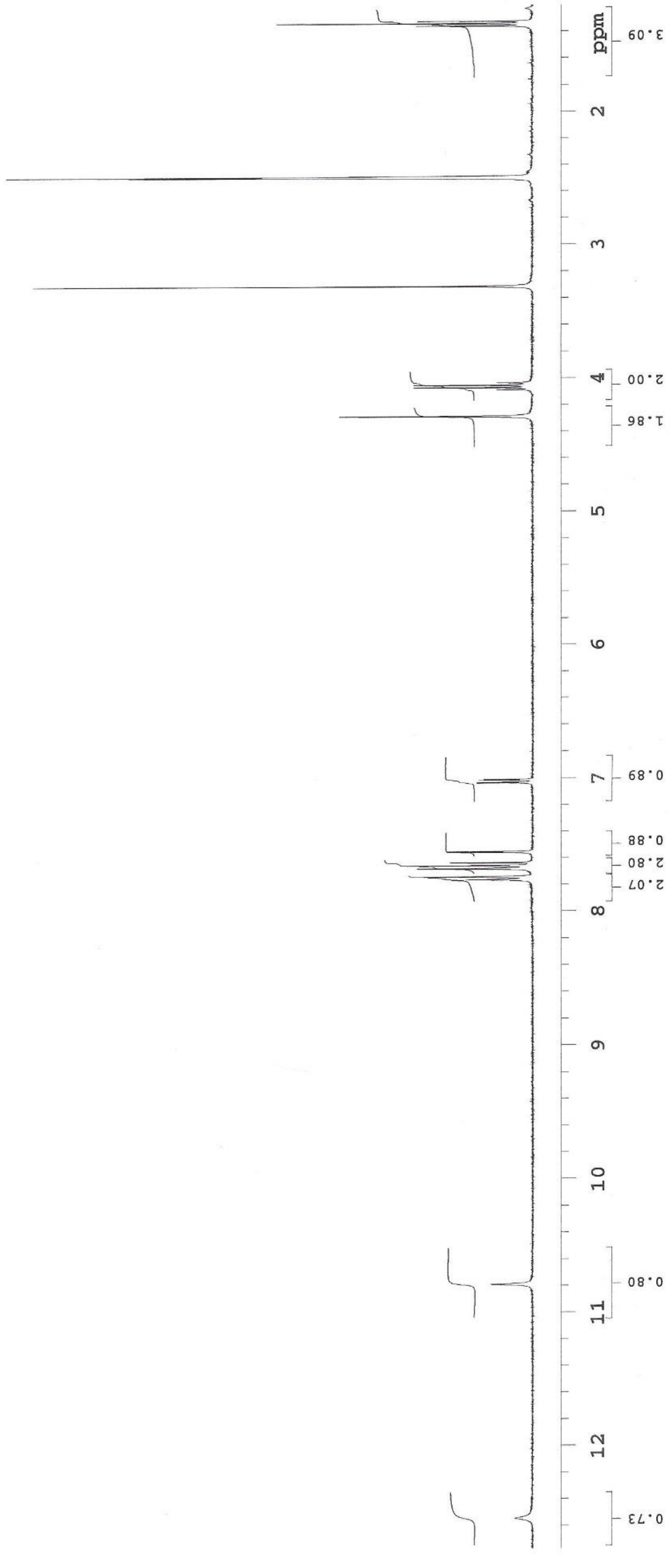

^{13}C NMR Spectrum of Compound **9**

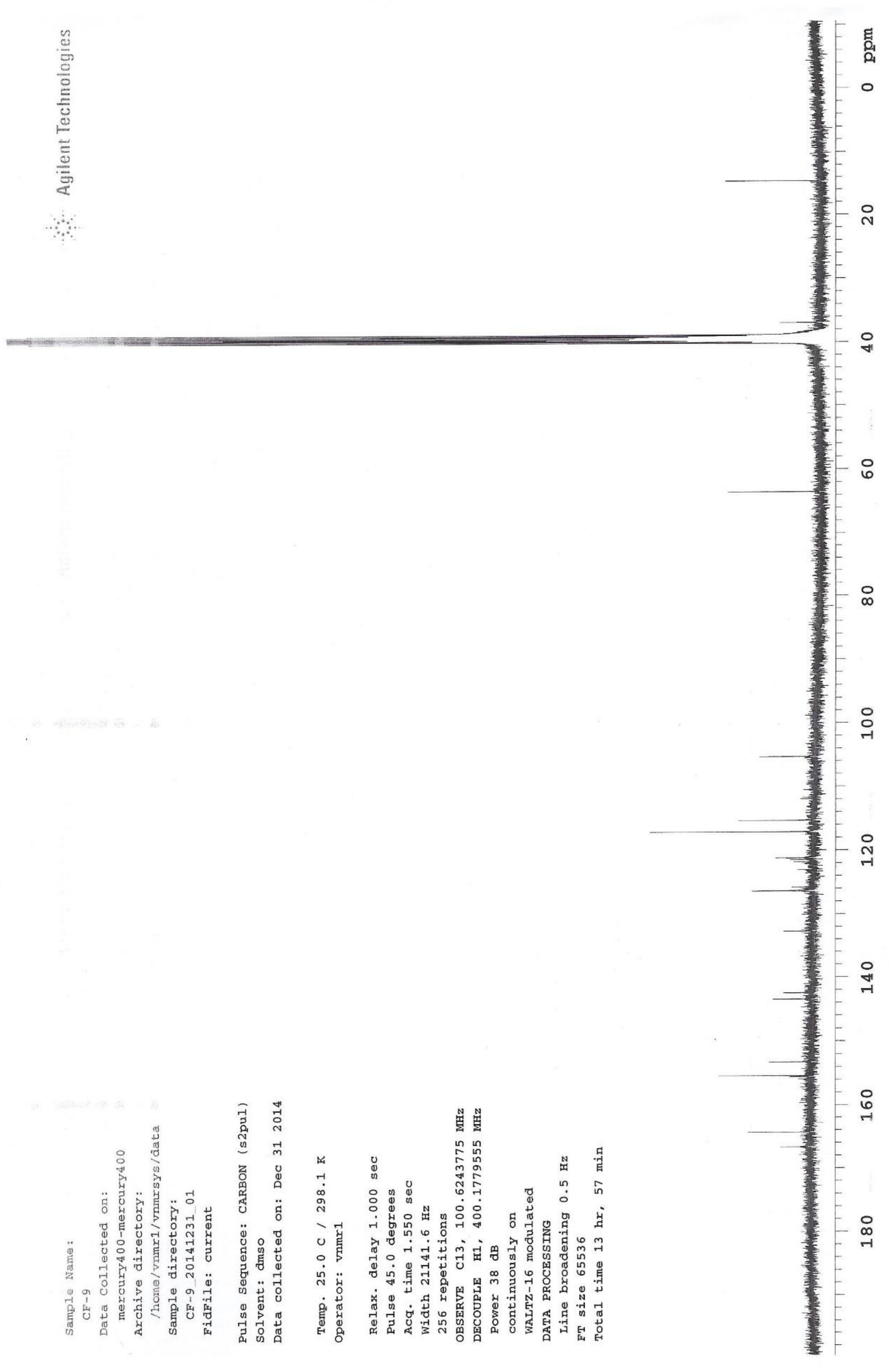

Mass Spectrum of Compound **9**

Formula Predictor Report - CF-9_20.lcd

Data File: C:\LabSolutions\Data\Analiz\mdaltintop\CF-9_20.lcd

Elmt	Val.	Min	Max	Elmt	Val.	Min	Max	Elmt	Val.	Min	Max	Elmt	Val.	Min	Max	Use Adduct
H	1	10	30	O	2	1	3	Cl	1	0	0	I	3	0	0	H
C	4	10	26	F	1	3	3	Br	1	0	0					
N	3	3	5	S	2	2	3	Ru	2	0	0					

Error Margin (ppm): 5
HC Ratio: unlimited
Max Isotopes: 3
MSn Iso RI (%): 10.00

DBE Range: 14.0 - 20.0
Apply N Rule: no
Isotope RI (%): 1.00
MSn Logic Mode: AND

Electron Ions: both
Use MSn Info: no
Isotope Res: 10000
Max Results: 500

Event#: 1 MS(E+) Ret. Time : 7.507 Scan# : 1127

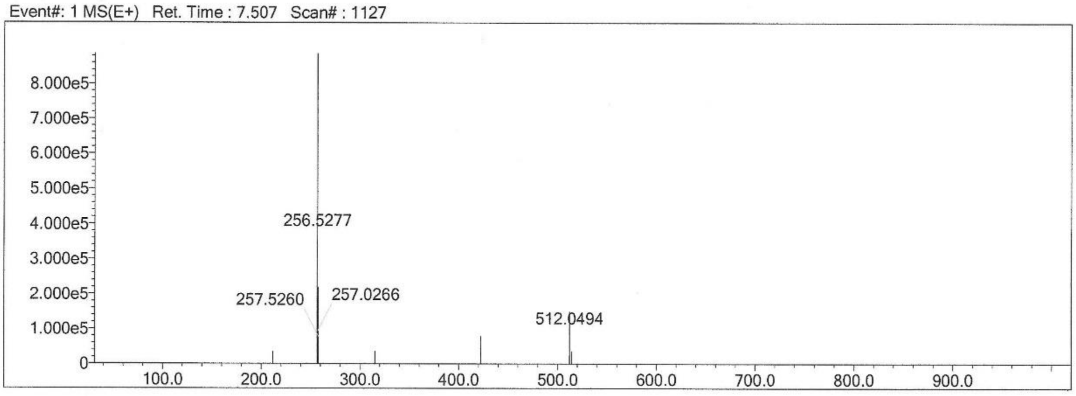

Measured region for 512.0494 m/z

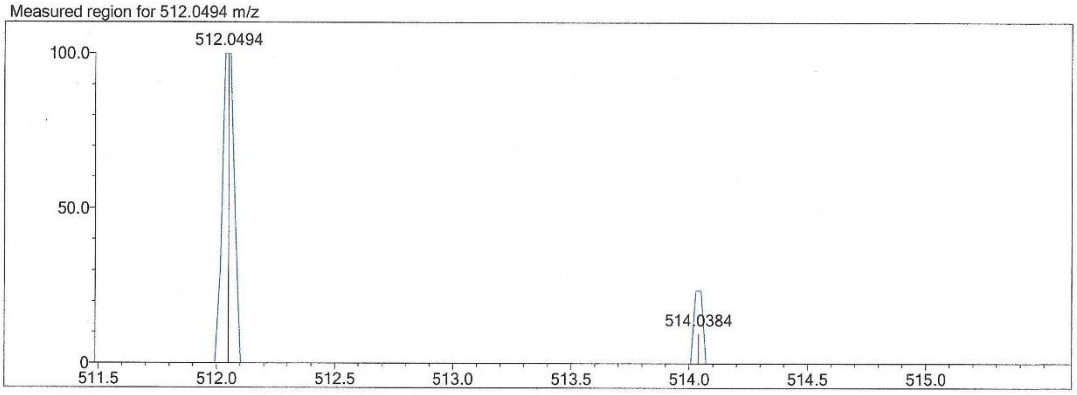

C20 H16 N5 O2 F3 S3 [M+H]+ : Predicted region for 512.0491 m/z

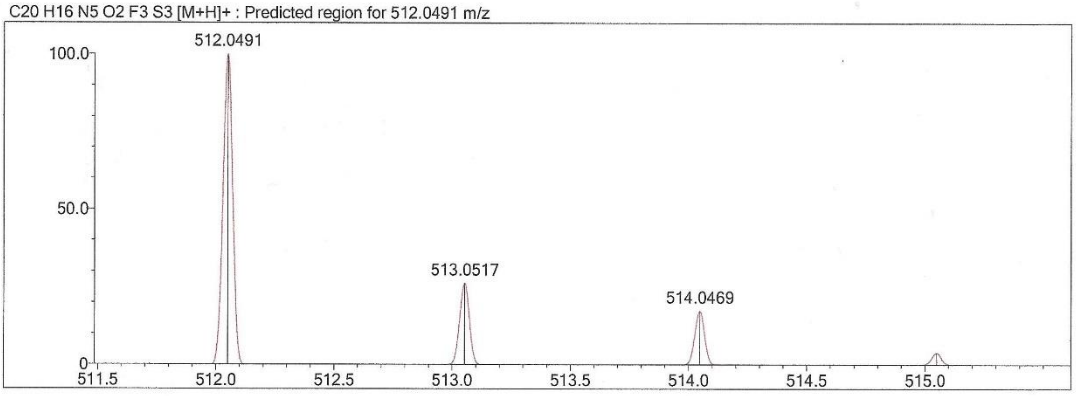

Rank	Score	Formula (M)	Ion	Meas. m/z	Pred. m/z	Df. (mDa)	Df. (ppm)	Iso	DBE
1	57.05	C20 H16 N5 O2 F3 S3	[M+H]+	512.0494	512.0491	0.3	0.59	57.05	14.0

IR Spectrum of Compound **10**

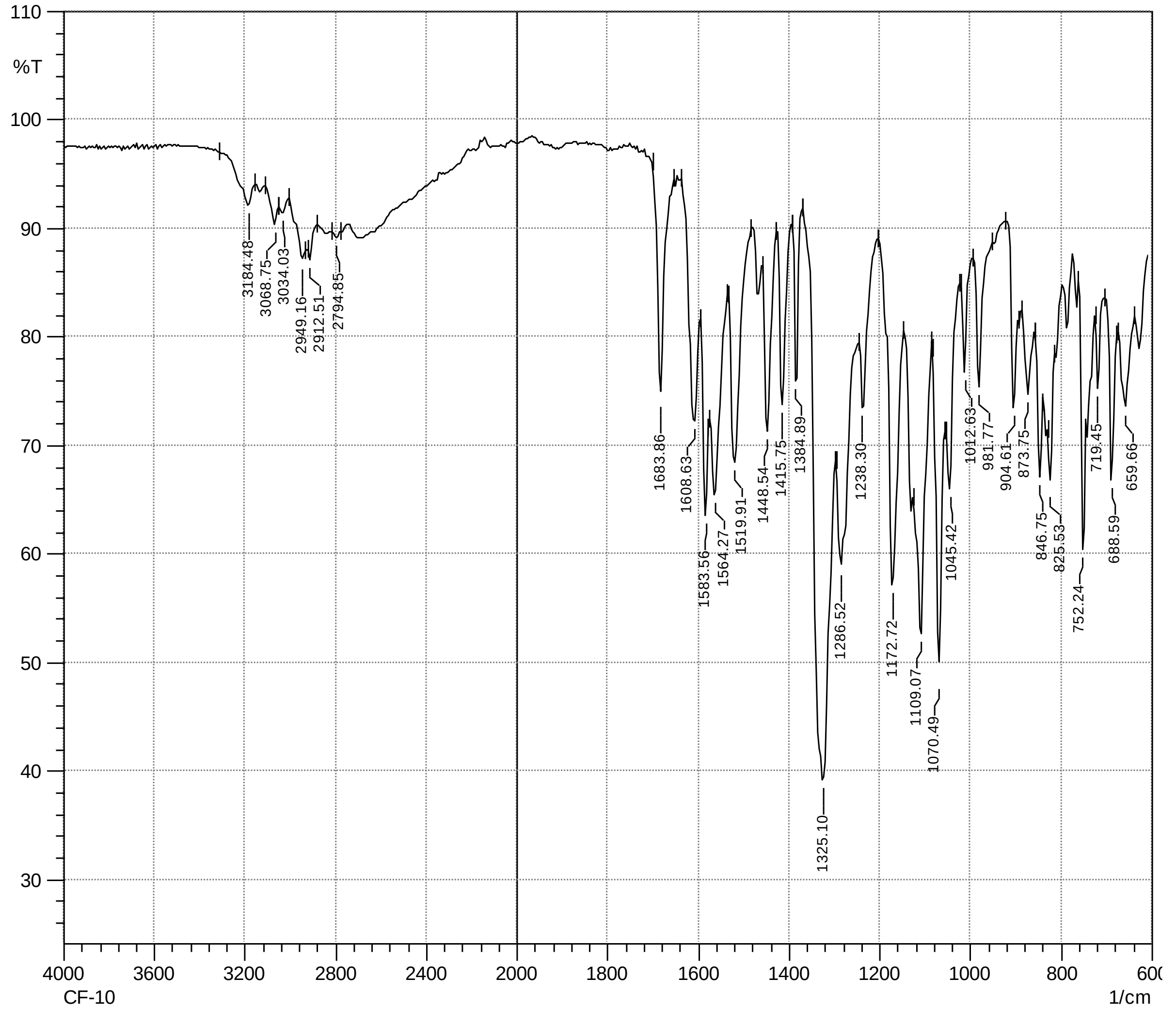

^{1}H NMR Spectrum of Compound **10**

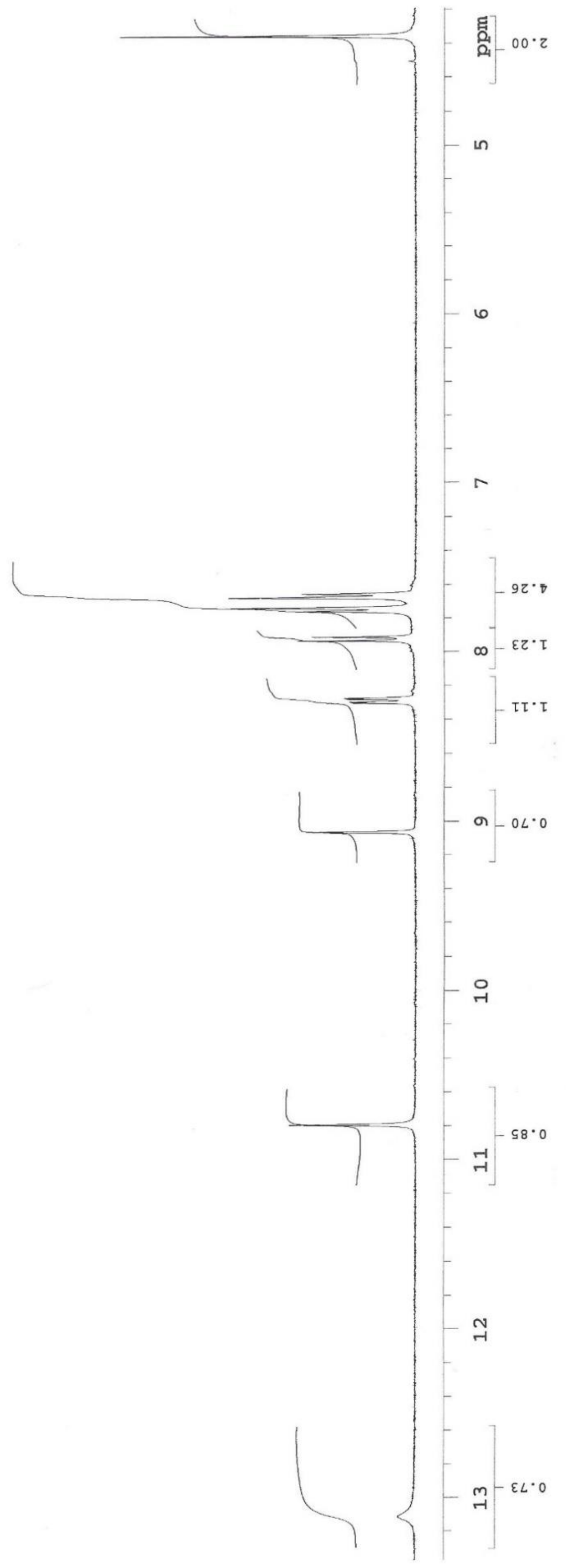

^{13}C NMR Spectrum of Compound **10**

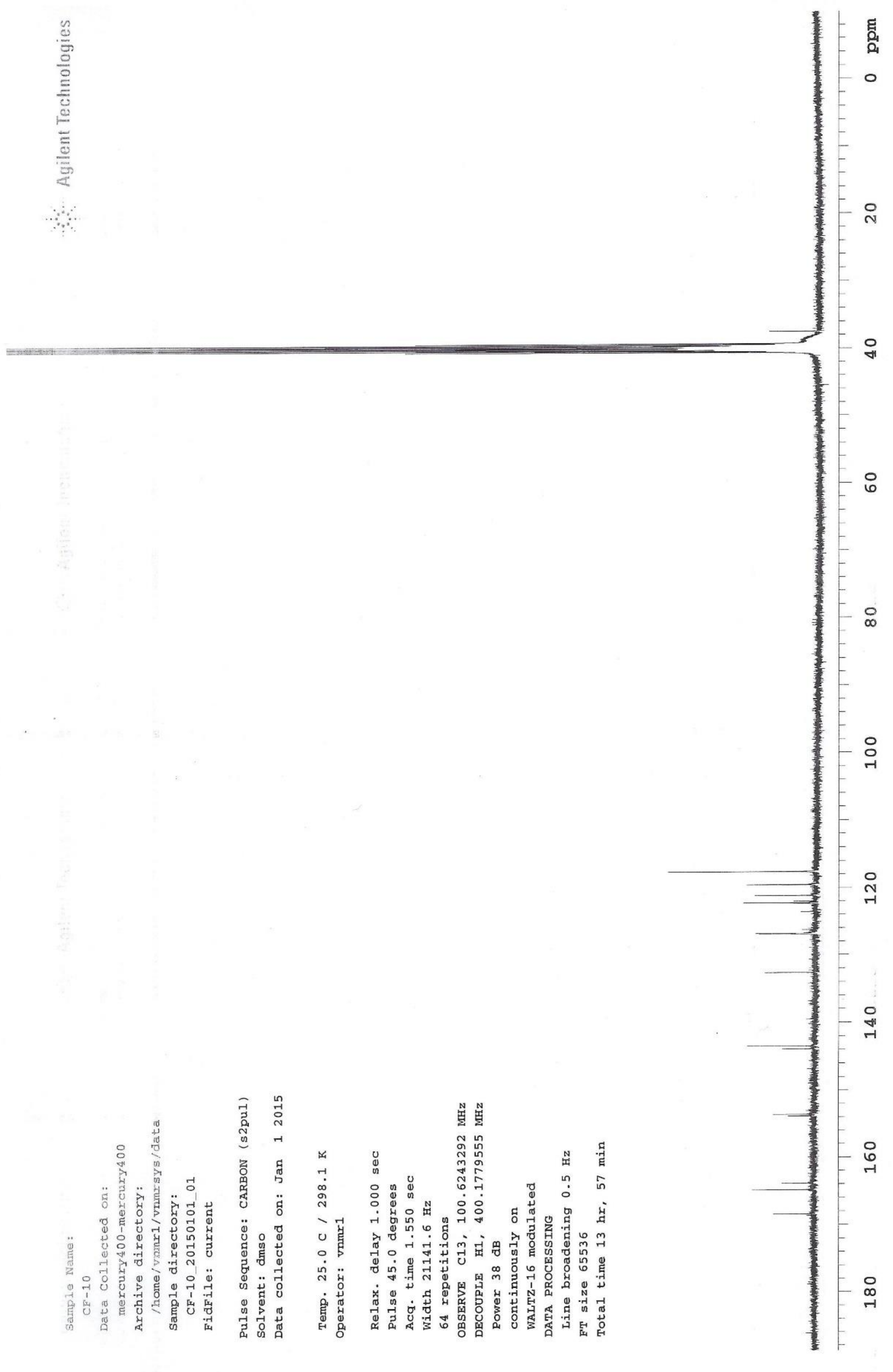

Mass Spectrum of Compound **10**

Formula Predictor Report - CF-10_1.lcd

Data File: C:\LabSolutions\Data\Analiz\mdaltintop\CF-10_1.lcd

Elmt	Val.	Min	Max	Elmt	Val.	Min	Max	Elmt	Val.	Min	Max	Elmt	Val.	Min	Max	Use Adduct
H	1	10	30	O	2	1	3	Cl	1	0	0	I	3	0	0	H
C	4	10	26	F	1	3	4	Br	1	0	0					
N	3	3	6	S	2	3	3	Ru	2	0	0					

Error Margin (ppm): 5
HC Ratio: unlimited
Max Isotopes: 3
MSn Iso RI (%): 10.00

DBE Range: 14.0 - 20.0
Apply N Rule: no
Isotope RI (%): 1.00
MSn Logic Mode: AND

Electron Ions: both
Use MSn Info: no
Isotope Res: 10000
Max Results: 500

Event#: 1 MS(E+) Ret. Time : 7.227 -> 7.373 Scan# : 1085 -> 1107

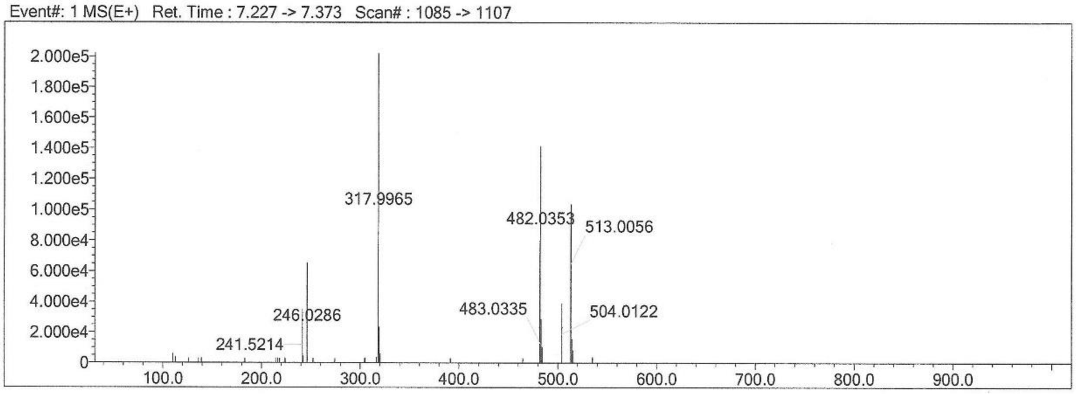

Measured region for 513.0056 m/z

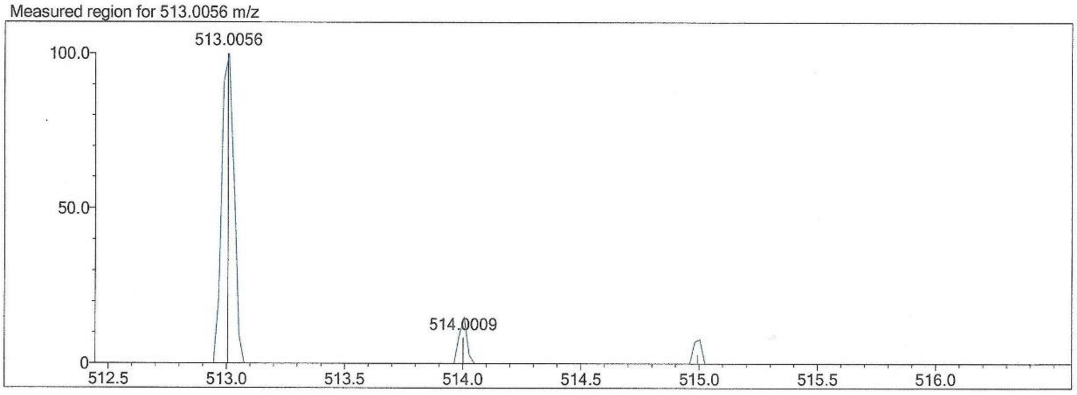

C18 H11 N6 O3 F3 S3 [M+H]+ : Predicted region for 513.0080 m/z

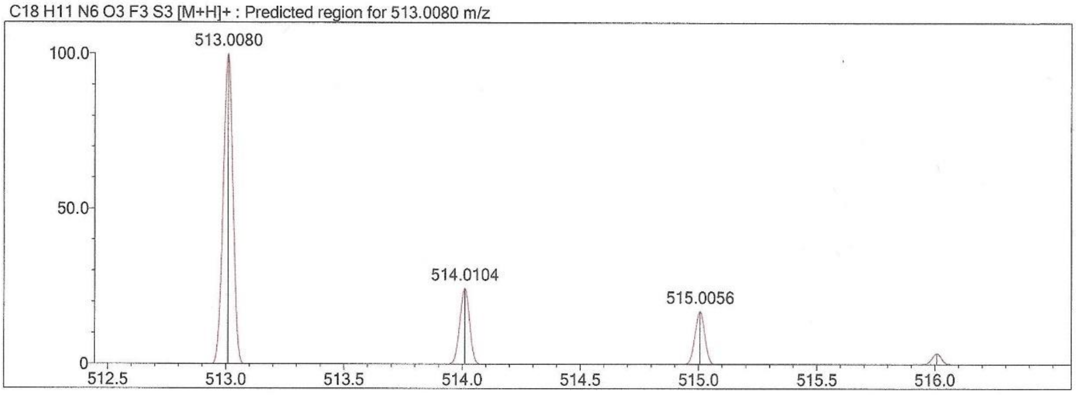

Rank	Score	Formula (M)	Ion	Meas. m/z	Pred. m/z	Df. (mDa)	Df. (ppm)	Iso	DBE
1	30.19	C18 H11 N6 O3 F3 S3	[M+H]+	513.0056	513.0080	-2.4	-4.68	33.25	15.0
